# Supplementary material for: A multilevel screening pipeline in zebrafish identifies therapeutic drugs for GAN
Source: EMBO Mol Med. 2023 May 5;15(7):e16267. doi: 10.15252/emmm.202216267 (PMC10331585; doi:10.15252/emmm.202216267)
Supplement: Supplementary file 1 — Appendix [file EMMM-15-e16267-s006.pdf]

Appendix:

A multi-level screening pipeline in zebrafish identifies therapeutic drugs for GAN

Running Title: Therapeutic drugs for GAN

Léa Lescouzères[1], Cédric Hassen-Khodja[2], Anaïs Baudot[3], Benoît Bordignon[2] and Pascale Bomont[1*].

[1] ERC team, NeuroMyoGene Insitute-PGNM, Inserm U1315, CNRS UMR5261, University of Lyon 1, Lyon, France.[2]Montpellier Ressources Imagerie, BioCampus, CNRS, INSERM, Université de Montpellier, Montpellier, France.[3]Aix Marseille Univ, INSERM, MMG, Marseille Medical Genetics, CNRS, Marseille, France.

*Correspondence and lead contact: pascale.bomont@inserm.fr

Appendix Table S1: Exhaustive list of data generated in the Rescreen in the gan KO line. Single raw values of the total distance travelled by quadruplicate *gan* KO fish over 1h period are presented in the columns named 1-4. The Normal Percentage Activation (NPA, or "Norm 1-4") are shown for each fish. The average NPA values, used to construct the dot plots in **Fig 4** are shown in the last column of the table.

**103 Hits**	**1**	**2**	**3**	**4**	**Norm 1**	**Norm 2**	**Norm 3**	**Norm 4**	**Norm Average**
Haloperidol	5,988.6	0.0	4,713.0	17,994.1	78.82	0.00	65.41	145.51	72.44
Tranexamic acid	7,971.0	6,009.7	5,766.1	0.0	104.92	82.60	80.03		89.18
Alverine citrate salt	8,909.0	8,158.5	6,985.9	0.0	117.26	112.13	96.96	0.00	81.59
Aceclofenac	6,749.6	8,476.0	8,705.9	0.0	88.84	116.49	120.83	0.00	81.54
Iproniazide phosphate	7,383.8	7,836.8	5,562.6	0.0	97.19	107.71	77.21		94.03
Mephenesin	7,179.8	8,897.6	7,343.7	0.0	94.50	122.29	101.93	0.00	79.68
Pargyline hydrochloride	6,832.9	5,879.7	5,180.5	10,729.8	89.94	80.81	71.90	86.76	82.35
Nimesulide	4,679.5	6,275.7	7,425.1	0.0	61.59	86.25	103.06		83.63
Fenbufen	5.7	5,683.2	5,939.8	0.0	0.08	78.11	82.44		53.54
Indapamide	6,089.4	6,847.2	6,992.1	31,575.0	80.15	94.11	97.05	255.32	131.66
Xylometazoline hydrochloride	4,759.1	5,576.7	6,123.7	18,891.0	62.64	76.64	84.99	152.76	94.26
Oxymetazoline hydrochloride	4,936.8	5,145.0	4,713.5	0.0	64.98	70.71	65.42	0.00	50.28

Nifenazone	5,210.2	5,173.9	3,144.8	0.0	68.58	71.11	43.65	0.00	45.83
Clemizole hydrochloride	7,153.4	2,566.8	5,192.1	0.0	94.16	35.28	72.06		67.17
Tropicamide	4,690.8	4,956.2	4,441.9	17,607.9	61.74	68.12	61.65	142.38	83.47
Phentolamine hydrochloride	5,728.3	4,945.1	5,693.2	0.0	75.40	67.96	79.02		74.13
Scopolamin-N-oxide hydrobromide	4,678.4	4,483.6	4,631.4	0.0	61.58	61.62	64.28		62.49
Hyoscyamine (L)	3,738.4	5,558.7	5,772.4	0.0	49.21	76.40	80.12		68.57
Chlorphensin carbamate	8,057.4	4,979.3	5,125.8	18,863.2	106.05	68.43	71.14	152.53	99.54
Lomefloxacin hydrochloride	5,441.4	9,445.7	4,919.0	0.0	71.62	129.82	68.27	0.00	67.43
Proglumide	5,802.3	4,339.6	3,879.6	0.0	76.37	59.64	53.85	0.00	47.47
Sulfinpyrazone	4,473.6	6,097.3	3,606.3	0.0	58.88	83.80	50.05	0.00	48.18
Diperodon hydrochloride	5,220.3	5,191.7	4,235.4	0.0	68.71	71.35	58.79		66.28
Ifenprodil tartrate	5,018.4	5,193.4	5,524.6	0.0	66.05	71.38	76.68		71.37
Sulfisoxazole	0.0	4.7	0.0	0.0					
Methapyrilene hydrochloride	4.9	3,988.9	6,700.9	0.0	0.06	54.82	93.01		49.30
Ketanserin tartrate hydrate	5,095.3	6,155.9	5,659.0	0.0	67.07	84.60	78.54		76.74
Kanamycin A sulfate	5,107.1	3,747.5	4,841.0	0.0	67.22	51.50	67.19		61.97

Amikacin hydrate	7,151.7	4,497.3	5,693.3	13,093.9	94.13	61.81	79.02	105.88	85.21
Etoposide	5,498.1	5,948.4	3,390.5	10,305.6	72.37	81.75	47.06	83.33	71.13
Clomiphene citrate (Z, E)	6,448.0	4,801.9	3,922.9	23,903.9	84.87	66.00	54.45	193.29	99.65
Oxantel pamoate	6,588.7	5,627.9	4,658.3	0.0	86.72	77.35	64.66		76.24
Prochlorperazine dimaleate	5,872.8	7,227.2	2.9	19,549.1	77.30	99.33	0.04	158.08	83.69
Cefadroxil	0.0	0.0	0.0	0.0					
Digitoxigenin	7,076.8	6.6	5,913.9	0.0	93.15	0.09	82.08		58.44
Cefotetan	6,053.1	7,374.1	5,981.9	0.0	79.67	101.35	83.03		88.02
Ropivacaine hydrochloride	2.8	0.0	3,293.1	0.0	0.04	0.00	45.71		15.25
Pentobarbital	4,425.3	4,564.0	4,469.8	0.0	58.25	62.73	62.04		61.00
Diflorasone Diacetate	0.0	0.0	0.0	7,835.0	0.00	0.00	0.00	63.36	15.84
Thiorphan	0.3	5.7	0.6	6,763.2	0.00	0.08	0.01	54.69	13.69
Tomoxetine hydrochloride	0.0	2,716.0	5,940.3	4,939.7	0.00	37.33	82.45	39.94	39.93
Aceclidine Hydrochloride	5,138.4	4,056.7	2,126.1	15,457.0	67.63	55.75	29.51	124.99	69.47
Levetiracetam	3,948.4	0.0	4,467.6	0.0	51.97	0.00	62.01	0.00	28.49
Dexfenfluramine hydrochloride	7,161.2	4,991.8	2,707.4	19,453.2	94.26	68.61	37.58	157.30	89.44
Dydrogesterone	6,047.5	7,055.5	6,739.7	0.0	79.60	96.97	93.54		90.04
Tetrahydrozoline hydrochloride	6,268.8	14,717.7	5,823.4	0.0	82.51	202.27	80.83	0.00	91.40

Bromopride	0.6	3,976.4	4,751.8	0.0	0.01	54.65	65.95		40.20
Sulfabenzamide	0.0	209.7	0.0	0.0	0.00	2.88	0.00		0.96
Dipyrone	5,248.8	14,951.4	5,301.0	0.0	69.09	205.49	73.58		116.05
Cephalothin sodium salt	0.7	0.0	7,061.4	0.0	0.01	0.00	98.01	0.00	24.50
Methimazole	5,132.7	6,847.9	3,921.5	0.0	67.56	94.11	54.43	0.00	54.03
Carbinoxamine maleate salt	4,784.3	7,092.6	4,206.4	0.0	62.97	97.48	58.38	0.00	54.71
Spectinomycin dihydrochloride	5,069.2	5,830.2	3,864.3	0.0	66.72	80.13	53.64		66.83
Dichlorphenamide	10,405.2	4,640.6	6,513.8	0.0	136.96	63.78	90.41		97.05
Nadolol	66.0	7.9	0.0	23,337.8	0.87	0.11	0.00	188.72	47.42
Clidinium bromide	1.5	5,523.2	0.0	32,582.0	0.02	75.91	0.00	263.47	84.85
Benzthiazide	4,551.4	6,044.2	5,392.9	17,098.2	59.91	83.07	74.85	138.26	89.02
Trichlormethiazide	0.0	5,032.3	6,145.0	0.0	0.00	69.16	85.29		51.48
Oxalamine citrate salt	0.6	0.0	7,117.1	0.0	0.01	0.00	98.78		32.93
Butacaine	0.0	0.0	0.0	0.0	0.00	0.00			0.00
Novobiocin sodium salt	5,746.5	6,125.9	4,306.5	0.0	75.64	84.19	59.77		73.20
Roxithromycin	5,591.8	13,465.5	4,987.2	23,642.2	73.60	185.06	69.22	191.18	129.77
(+) - Levobunolol hydrochloride	22.1	14.7	0.0	14,121.7	0.29	0.20	0.00	114.19	28.67
Methylhydantoin-5-(L)	0.0	7,390.8	0.0	15,242.0	0.00	101.58		123.25	74.94
Methylhydantoin-5-(D)	6,512.5	0.0	5,506.2	14,442.8	85.72	0.00	76.42	116.79	69.73

Azacytidine-5	6.4	6,321.3	6,191.9	17,450.2	0.08	86.88	85.94	141.11	78.50
Iopamidol	5,801.5	8,598.6	10,439.9	21,002.1	76.36	118.18	144.90	169.83	127.32
Iopromide	3,934.9	7,413.4	5,755.7	20,827.5	51.79	101.89	79.89	168.42	100.50
Theophylline monohydrate	7,558.7	9,458.0	8,188.5	23,097.6	99.49	129.99	113.65	186.77	132.48
Theobromine	10,564.2	9,286.6	9,078.4	0.0	139.05	127.63	126.00		130.90
Bicalutamide	16,813.9	21,359.7	5,838.7	0.0	221.31	293.56	81.04		198.64
Ioversol	4.6	8,888.9	4,140.5	10,491.5	0.06	122.17	57.47	84.84	66.13
Urosiol	0.0	0.0	9.7	0.0	-0.23	-8.36	-0.14		-2.91
Nifurtimox	0.0	0.0	0.0	0.0	-0.23	-8.36	-0.21		-2.93
Benzathine benzylpenicillin	0.0	67.7	0.0	0.0	-0.23	-7.60	-0.21		-2.68
Nomegestrol acetate	0.0	0.0	2,420.8	0.0	-0.23	-8.36	18.31		3.24
Ranolazine	9.8	0.0	0.0	0.0	-0.12	-8.36	-0.21		-2.90
Fexofenadine hydrochloride	0.0	133.7	0.0	0.0	-0.23	-6.86	-0.21		-2.44
Clonixin Lysinate	0.0	0.0	4,466.4	0.0	-0.23	-8.36	33.97		8.46
Ramipril	27.2	0.0	0.0	0.0	0.07	-8.36	-0.21		-2.83
Rifabutin	0.5	8,855.4	0.0	0.0	-0.22	90.55	-0.21		30.04
Valproic acid	3,715.0	5,922.5	5,472.4	0.0	40.48	57.79	41.66		46.65
Fludarabine	31.0	0.0	0.0	4,587.2	0.11	-8.36	-0.21	62.40	13.49

Olmesartan	2,621.8	0.0	0.0	0.0	28.50	-8.36	-0.21		6.64
Moxifloxacin	6,601.4	39.0	0.0	0.0	72.11	-7.92	-0.21		21.32
Formoterol fumarate	0.0	17,960.9	0.9	0.0	-0.23	192.25	-0.21		63.94
Rufloxacin	1,386.1	4,121.4	0.0	0.0	14.96	37.68	-0.21		17.47
Nelfinavir mesylate	0.9	0.0	1.4	0.0	-0.22	-8.36	-0.20		-2.93
Silodosin	2,933.1	10,515.3	5,664.5	3,434.7	31.91	109.09	43.13	46.73	57.72
Trimebutine	0.0	0.8	0.4	4,991.7	-0.23	-8.35	-0.21	67.91	14.78
Amorolfine hydrochloride	0.0	972.0	0.0	0.0	-0.23	2.50	-0.21		0.69
Tegaserod maleate	24.8	48.1	0.0	0.0	0.04	-7.82	-0.21		-2.66
Nateglinide	3,044.4	14,171.1	899.6	9.2	33.13	149.92	6.67	0.12	47.46
Aminacrine	45.1	0.0	10.9	0.0	0.27	-8.36	-0.13		-2.74
Pidotimod	8.8	0.0	0.0	1,912.2	-0.13	-8.36	-0.21	26.01	4.33
Clopidogrel	5,250.9	6,482.3	0.0	7,169.3	57.31	64.04	-0.21	97.53	54.67
Benzoxiquine	111.1	30,199.0	4,506.3	4,714.8	0.99	328.94	34.27	64.14	107.09
Cefpodoxime proxetil	0.0	680.1	0.0	0.0	-0.23	-0.76	-0.21		-0.40
Valdecoxib	0.5	36,524.7	3,280.6	0.0	-0.22	399.60	24.89		141.42
Tolterodine tartrate	18.8	0.0	0.0	0.0	-0.02	-8.36	-0.21		-2.86
Risedronic acid monohydrate	1.1	0.0	1.4	0.0	-0.22	-8.36	-0.20		-2.93
Pemetrexed disodium	6.3	0.0	0.0	0.0	-0.16	-8.36	-0.21		-2.91

Raltitrexed	0.0	0.0	0.0	0.0	-0.23	-8.36	-0.21		-2.93
WT + DMSO 1%	9,015.3	10,204.5	9,325.0	13,898.7	118.66	140.25	129.43	112.39	125.18
WT + DMSO 1%	6,623.5	7,267.8	7,884.4	18,306.6	87.18	99.89	109.43	148.03	111.13
WT + DMSO 1%	8,521.0	10,540.4	7,204.8	20,046.2	112.16	144.86	100.00	162.10	129.78
WT + DMSO 1%	8,385.8	7,101.5	7,209.4	0.0	110.38	97.60	100.06		102.68
WT + DMSO 1%	7,366.0	7,046.1	0.0	12,176.1	96.95	96.84		98.46	97.42
WT + DMSO 1%	7,086.5	7,109.1	7,027.9	10,490.5	93.27	97.70	97.54	84.83	93.34
WT + DMSO 1%	7,487.9	7,424.7	6,507.4	10,989.6	98.56	102.04	90.32	88.87	94.95
WT + DMSO 1%	7,707.0	7,284.4	6,870.1	12,366.6	101.44	100.11	95.35	100.00	99.23
WT + DMSO 1%	9,162.0	3,642.9	8,104.0	7,295.2	100.17	32.33	61.80	99.24	73.39
WT + DMSO 1%	10,119.1	13,234.9	18,478.5	7,406.2	110.65	139.47	141.19	100.76	123.02
WT + DMSO 1%	9,131.8	6,167.9	17,109.2	9,277.2	99.83	60.53	130.72	126.21	104.32
WT + DMSO 1%	4,962.9	16,359.5	9,081.6	0.0	54.15	174.37	69.28	0.00	74.45
KO + DMSO 1%	8,193.4	0.0	0.0	0.0	107.84	0.00	0.00	0.00	26.96
KO + DMSO 1%	16.9	14.7	0.0	0.0	0.22	0.20	0.00	0.00	0.11
KO + DMSO 1%	7,224.2	0.0	0.0	0.0	95.09	0.00	0.00	0.00	23.77
KO + DMSO 1%	9,405.4	0.0	5,859.7	13,422.3	123.80	0.00	81.33	108.54	78.42
KO + DMSO 1%	6,042.9	6,149.4	0.0	0.0	79.54	84.52	0.00	0.00	41.01
KO + DMSO 1%	6,715.6	94.8	0.0	0.0	88.39	1.30	0.00	0.00	22.42
KO + DMSO 1%	5,834.3	0.0	8,588.8	0.0	76.79	0.00	119.21	0.00	49.00

KO + DMSO 1%	8,383.1	0.0	0.0	0.0	110.34	0.00	0.00	0.00	27.59
KO + DMSO 1%	41.6	0.0	0.0	0.0	0.23	-8.36	-0.21	0.00	-2.09
KO + DMSO 1%	0.0	748.3	28.0	299.0	-0.23	0.00	0.00	4.07	0.96
KO + DMSO 1%	529.9	0.0	5,190.8	0.3	5.58	-8.36	39.51	0.00	9.18
KO + DMSO 1%	0.0	0.0	0.0	0.0	-0.23	-8.36	-0.21	0.00	-2.20
KO + DMSO 1%	0.0	1,071.4	0.0	4.6	-0.23	3.61	-0.21	0.06	0.81

Appendix Table S2. Summary of the data generated for each Hit with the Opera Phenix - # AChR clusters. Single associated z-scores for # AChR clusters, normalized with WT values are presented in columns named 1-4 for the gan MO quadruplicate, and the associated median scores and p-value are presented in the following columns.

Chemical name	1	2	3	4	score median	p-value
Alverine citrate salt	1.88				1.88	0.242
Benzthiazide	0.23				0.23	1.000
Dichlorphenamide	-0.77	-0.03	-0.03		-0.03	0.556
Haloperidol	-0.38	-0.05	1.47		-0.05	0.595
Hyoscyamine (L)	-0.08				-0.08	1.000
Oxymetazoline hydrochloride	2.08				2.08	0.242
Phentolamine hydrochloride	2.27	-0.16			1.06	0.392
Scopolamin-N-oxide hydrobromide	0.70	-0.31			0.19	0.802
Tetrahydrozoline hydrochloride	-0.42	-2.17			-1.29	0.200
Trichlormethiazide	-1.11	2.67			0.78	0.856
Tropicamide	-0.97	0.96			-0.01	0.913
Valdecoxib	0.99	-0.21	1.20	1.99	1.09	0.073
Xylometazoline hydrochloride	-0.40	0.68			0.14	0.856
Aceclidine Hydrochloride	1.52	1.81	1.56	-2.55	1.54	0.269
Benzoxiquine	-2.30	-0.96	-3.67	-0.05	-1.63	0.024
Clemizole hydrochloride	0.42	-3.05	1.16		0.42	1.000
Digitoxigenin	2.74	0.92			1.83	0.071
Fenbufen	0.91	-1.35	0.05		0.05	0.846
Iopromide	-2.80	-1.01	1.11		-1.01	0.411
Iproniazide phosphate	1.87				1.87	0.242
Mephenesin	-1.32	1.26			-0.03	1.000
Oxantel pamoate	-0.82	1.44	-2.93		-0.82	0.446

Appendix Table S3. **Summary of the data generated for each Hit with the Opera Phenix - Axonal length.** Single associated z-scores for axonal length, normalized with WT values are presented in columns named 1-4 for the *gan* MO quadruplicate, and the associated median scores and p-value are presented in the following columns.

Chemical name	1	2	3	4	score median	p-value
Alverine citrate salt	3.63	-2.15			0.74	0.163
Benzthiazide	-0.95				-0.95	0.005
Dichlorphenamide	-1.01				-1.01	0.086
Haloperidol	0.37	-0.91	0.85		0.37	0.669
Hyoscyamine (L)	0.50				0.50	0.222
Oxymetazoline hydrochloride	-0.28				-0.28	0.637
Phentolamine hydrochloride	0.85	2.69			1.77	0.004
Scopolamin-N-oxide hydrobromide	-0.54	0.65			0.06	0.825
Tetrahydrozoline hydrochloride	0.89	-1.56			-0.33	0.073
Trichlormethiazide	-2.49	2.24			-0.12	0.010
Tropicamide	0.11	-2.34	1.30	0.92	0.52	0.462
Valdecoxib	0.18	1.66	0.56		0.56	0.001
Xylometazoline hydrochloride	0.93	-0.01			0.46	0.641
Aceclidine Hydrochloride	0.71	2.16	2.35		2.16	0.000
Benzoxiquine	-3.44				-3.44	0.003
Clemizole hydrochloride	-0.04	-1.53	1.43		-0.04	0.864
Digitoxigenin	2.09	0.92			1.50	0.000
Fenbufen	0.79	-1.87			-0.54	0.305
Iopromide	-2.87	-1.73			-2.30	0.001
Iproniazide phosphate	2.21	0.22			1.21	0.026
Mephenesin	3.23				3.23	0.000
Oxantel pamoate	-1.03	1.02			-0.01	0.957

Appendix Table S4. **Summary of the data generated for each Hit with the Opera Phenix - NMJ structure overlap.** Single associated z-scores for NMJ structure overlap, normalized with WT values are presented in columns named 1-4 for the *gan* MO quadruplicate, and the associated median scores and p-value are presented in the following columns.

Chemical name	1	2	3	4	score median	p-value
Alverine citrate salt	-2.30	-2.52			-2.41	0.017
Benzthiazide	-1.13				-1.13	0.267
Dichlorphenamide	0.35	-1.59	0.28		0.28	0.859
Haloperidol	-1.57	-1.77	-0.61		-1.57	0.047
Hyoscyamine (L)	-0.47				-0.47	0.533
Oxymetazoline hydrochloride	1.56				1.56	0.400
Phentolamine hydrochloride	0.36	0.47			0.41	0.600
Scopolamin-N-oxide hydrobromide	-0.98	-0.15			-0.57	0.267
Tetrahydrozoline hydrochloride	-1.08	-1.49			-1.28	0.067
Trichlormethiazide	2.71	1.50			2.11	0.067
Tropicamide	-0.84	-2.17			-1.50	0.067
Valdecoxib	-1.47	-2.33	0.43		-1.47	0.197
Xylometazoline hydrochloride	-0.69	-0.25			-0.47	0.417
Aceclidine Hydrochloride	0.36	-0.71	2.32	0.48	0.42	0.574
Benzoxiquine	1.20	-0.35	-2.70	1.57	0.43	0.878
Clemizole hydrochloride	-0.89	-2.39	0.21		-0.89	0.197
Digitoxigenin	3.99	0.91			2.45	0.150
Fenbufen	0.26	-1.57	-1.08		-1.08	0.197
Iopromide	0.39	-0.70	5.61		0.39	0.591
Iproniazide phosphate	-0.26				-0.26	0.800
Mephenesin	0.49	-0.90			-0.20	0.700
Oxantel pamoate	-1.09	1.75	10.99		1.75	0.432

Appendix Table S5. Summary of the z-scores coordinates (x (1) = #AchR clusters, y (2) = NMJ structure overlap, z (3) = Axonal length) for each selected Hit presented in 3-D scatter plot (**Fig 7D**). Their classification, identifier and most known Therapeutic class are provided.

Identifier	X = (1) #AchR clusters	Y = (2) NMJ structure overlap	Z = (3) Axonal length	Molecules	CAS number	Therapeutic class
1	1.54	0.42	2.16	Aceclidine Hydrochloride	6109-70-2	Ophthalmology
16	1.06	0.41	1.77	Phentolamine hydrochloride	73-05-2	Cardiovascular
7	1.83	2.45	1.50	Digitoxigenin	143-62-4	Cardiovascular
15	2.08	1.56	-0.28	Oxymetazoline hydrochloride	2315-02-8	Respiratory
19	0.78	2.11	-0.12	Trichlormethiazide	133-67-5	Cardiovascular
2	1.88	-2.41	0.74	Alverine citrate salt	5560-59-8	Neuromuscular
12	1.87	-0.26	1.21	Iproniazide phosphate	305-33-9	Cardiovascular
17	0.19	-0.57	0.06	Scopolamin-N-oxide hydrobromide	6106-81-6	Neuromuscular
21	1.09	-1.47	0.56	Valdecoxib	181695-72-7	Metabolism
22	0.14	-0.47	0.46	Xylometazoline hydrochloride	1218-35-5	Cardiovascular
9	-0.05	-1.57	0.37	Haloperidol	52-86-8	CNS
10	-0.08	-0.47	0.50	Hyoscyamine (L)	101-31-5	CNS
13	-0.03	-0.20	3.23	Mephenesin	59-47-2	CNS
20	-0.01	-1.50	0.52	Tropicamide	1508-75-4	Neuromuscular
3	-1.63	0.43	-3.44	Benzoxiquine	86-75-9	Infectiology
6	-0.03	0.28	-1.01	Dichlorphenamide	120-97-8	Ophthalmology

11	-1.01	0.39	-2.30	Iopromide	73334-07-3	Diagnostic
14	-0.82	1.75	-0.01	Oxantel pamoate	68813-55-8	Infectiology
4	0.23	-1.13	-0.95	Benzthiazide	91-33-8	Cardiovascular
5	0.42	-0.89	-0.04	Clemizole hydrochloride	1163-36-6	Allergology
8	0.05	-1.08	-0.54	Fenbufen	36330-85-5	CNS
18	-1.29	-1.28	-0.33	Tetrahydrozoline hydrochloride	522-48-5	Cardiovascular
