# Supplementary material for: A multilevel screening pipeline in zebrafish identifies therapeutic drugs for GAN
Source: EMBO Mol Med. 2023 May 5;15(7):e16267. doi: 10.15252/emmm.202216267 (PMC10331585; doi:10.15252/emmm.202216267)
Supplement: Supplementary file 2 — Expanded View Figures PDF [file EMMM-15-e16267-s003.pdf]

Expanded View Figures

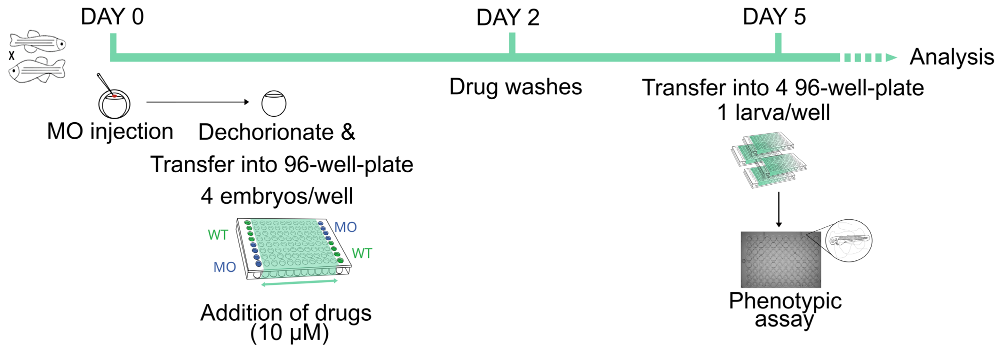

Figure EV1. Schematic representation of the timeline and workflow of the drug screening in the *gan* zebrafish model.

At 0 hpf, WT eggs are injected with *gan* morpholino (MO injection). At 6 hpf, noninjected WT and MO-injected eggs are dechorionated and distributed in 96-well plates (4 eggs/well) and plates are incubated at 8 hpf in fish water with/without drugs at 10 μM concentration. At 2 dpf, drugs are washed. At 5 dpf, larvae are transferred in quadruplicate plates (1 larva/well) and processed to the motility assay.

Figure EV2. Exhaustive representation of the z-scores obtained in the Screen for the 1,280 compounds.

For each drug, individual z-scores of quadruplicate fish are plotted and assessed for the total distance traveled at 5 dpf by *gan* MO-injected larvae for 1 h following treatment with compound. Dotted lines show the mean z-score for untreated *gan* MO-injected larvae (blue) and non-injected WT larvae (red). Toxic drugs are represented in gray (without associated z-score), non-Hits in red, B Hits in orange and A Hits in green.

© 2023 The Authors

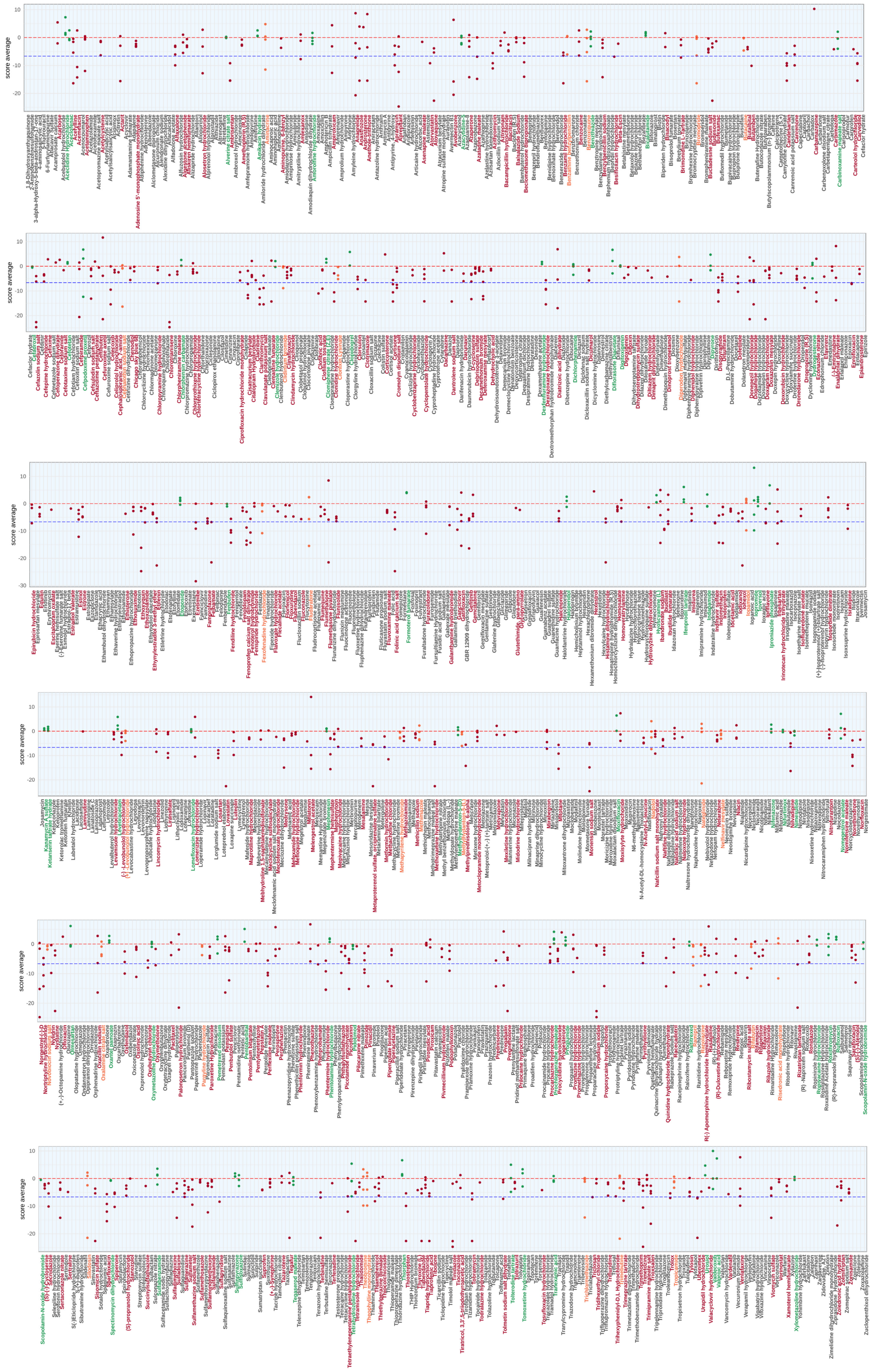

Figure EV2.

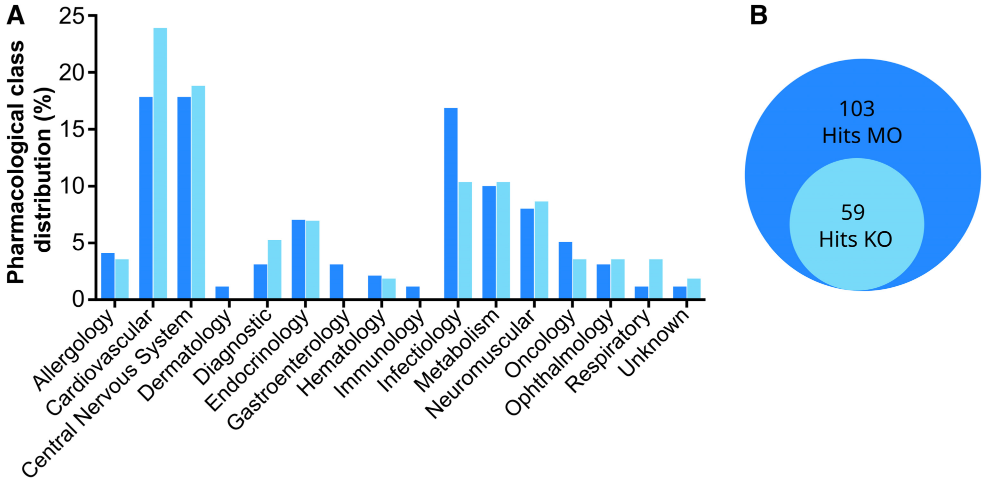

Figure EV3. Pharmacological class of the Hits.

A, B Bar plot (A) showing the distribution of the therapeutic classes (%) of the 59 Hits common to the *gan* morphants and the *gan* KO line (B), according to the Prestwick Chemical Library annotations.

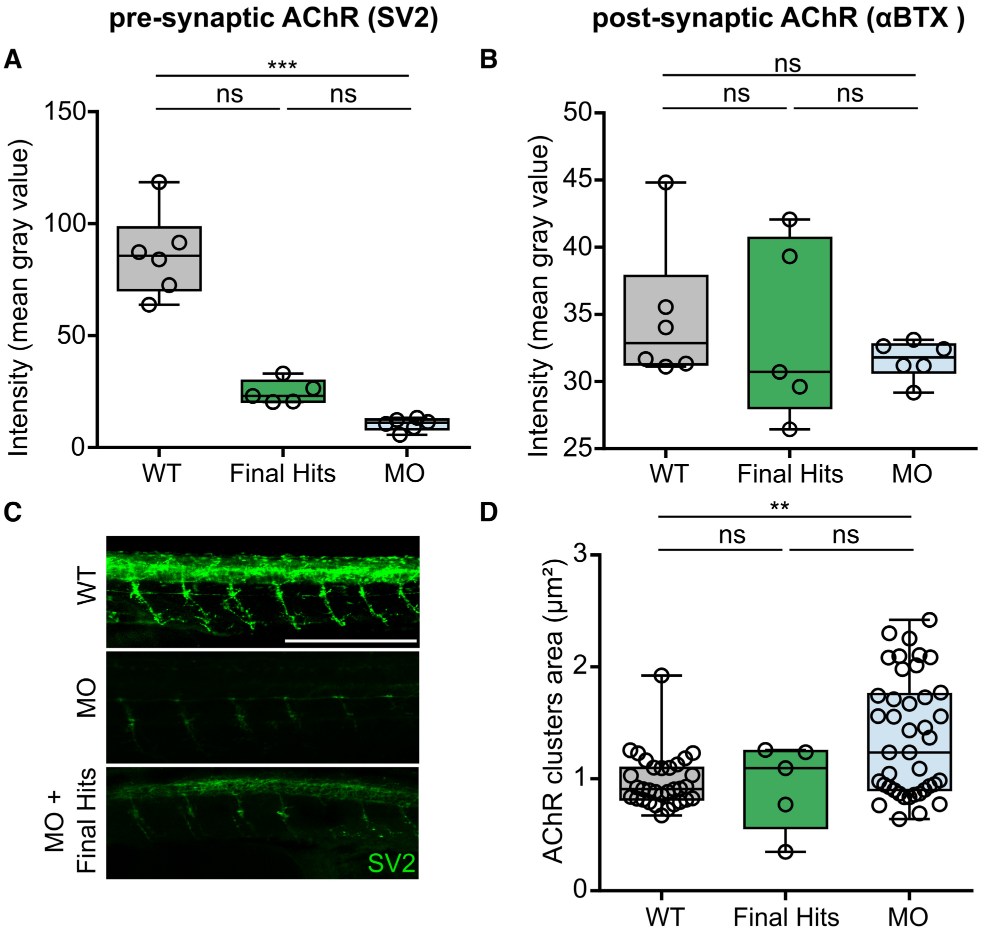

Figure EV4.

 © 2023 The Authors

Figure EV4. Additional AChR-related phenotypes in the *gan* zebrafish, which are not rescued by the five Final Hits.

A–D Boxplots showing individual and mean values of the labeling intensity of presynaptic (stained with Synaptic Vesicle glycoprotein 2 (SV2)) (A) and postsynaptic (stained with α-bungarotoxin (αBTX)) (B) AChR clusters and area of post-synaptic AChR (D) for three groups: noninjected WT (black), MO-injected embryos (blue), MO-injected embryos treated with the five Final Hits (dark green), as identified in Fig 7. (C) Representative images of SV2 intensity labeling for noninjected WT embryos (WT), *gan* MO-injected embryos (MO) and *gan* MO-injected embryos treated with Phentolamine Hydrochloride. Analysis performed at 48 hpf. Scale bar represents a length of 500 μm. Each dot represents individual values for WT ($n = 6$ (A, B), $n = 34$ (D)) and MO ($n = 6$ (A, B), $n = 43$ (D)), and mean values of quadruplicate treated larvae ($n = 5$ (A, B, D)) with single Hits (A, B, D). The central bands of the boxplots represent the median, the boxes of the boxplots represent the interquartile range (between the first and third quartile), and the whiskers represent the minimum and maximum values.

In the absence of normality of distribution of the data, a nonparametric Kruskal–Wallis test is applied; medians with range are represented; $^{**}P \leq 0.01$, $^{***}P \leq 0.001$.

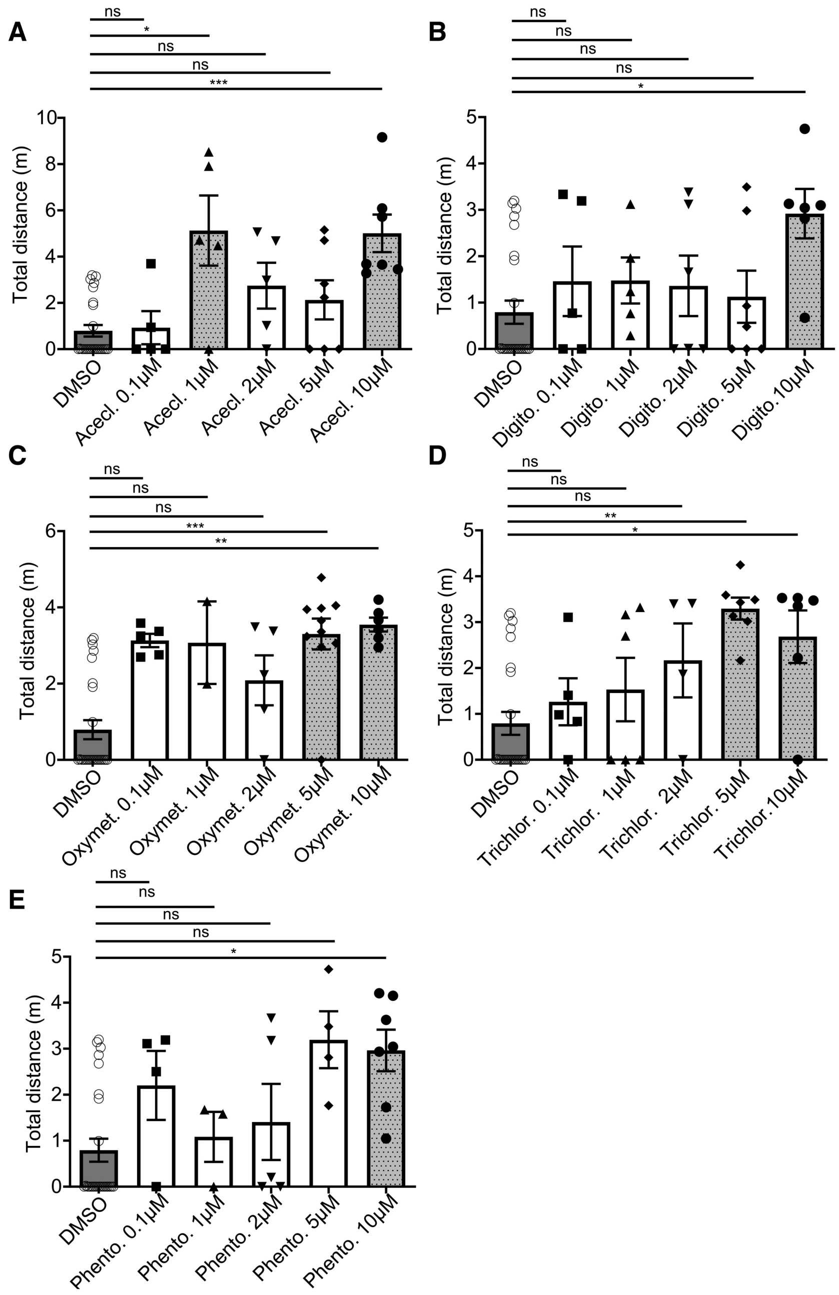

Figure EV5. Dose–response effect of the five favorite Hits on the restoration of motility.

A–E Treatment with Aceclidine Hydrochloride (Acecl.) (A), Digitoxigenin (Digito.) (B), Oxymetazoline Hydrochloride (Oxymet.) (C), Trichlormethiazide (Trichlor.) (D) and Phentolamine Hydrochloride (Phento.) (E) was performed from 0.1 to 50 μM.

Data information: Data are not provided for the higher doses (30 and 50 μM) due to toxicity. Results show the distance traveled at 5 dpf, during 1 h by *gan* MO-injected larvae, treated with either DMSO or Hits from 0.1 to 10 μM.

Each dot represents individual values, (A) $n = 25$ (MO + DMSO), $n = 5$ (MO + Acecl. 0.1 μM), $n = 5$ (MO + Acecl. 1 μM), $n = 5$ (MO + Acecl. 2 μM), $n = 7$ (MO + Acecl. 5 μM), $n = 7$ (MO + Acecl. 10 μM); (B) $n = 25$ (MO + DMSO), $n = 5$ (MO + Digito. 0.1 μM), $n = 5$ (MO + Digito. 1 μM), $n = 6$ (MO + Digito. 2 μM), $n = 7$ (MO + Digito. 5 μM), $n = 5$ (MO + Digito. 10 μM); (C) $n = 25$ (MO + DMSO), $n = 5$ (MO + Oxymet. 0.1 μM), $n = 2$ (MO + Oxymet. 1 μM), $n = 5$ (MO + Oxymet. 2 μM), $n = 10$ (MO + Oxymet. 5 μM), $n = 6$ (MO + Oxymet. 10 μM); (D) $n = 25$ (MO + DMSO), $n = 5$ (MO + Trichlor. 0.1 μM), $n = 6$ (MO + Trichlor. 1 μM), $n = 4$ (MO + Trichlor. 2 μM), $n = 6$ (MO + Trichlor. 5 μM), $n = 6$ (MO + Trichlor. 10 μM); (E) $n = 25$ (MO + DMSO), $n = 4$ (MO + Phento. 0.1 μM), $n = 3$ (MO + Phento. 1 μM), $n = 5$ (MO + Phento. 2 μM), $n = 4$ (MO + Phento. 5 μM), $n = 7$ (MO + Phento. 10 μM).

In the absence of normality of distribution of the data, a nonparametric Kruskal–Wallis test is applied; means ± SEM are represented. $^{*}P \leq 0.05$; $^{**}P \leq 0.01$, $^{***}P \leq 0.001$ and $^{****}P \leq 0.0001$.

© 2023 The Authors

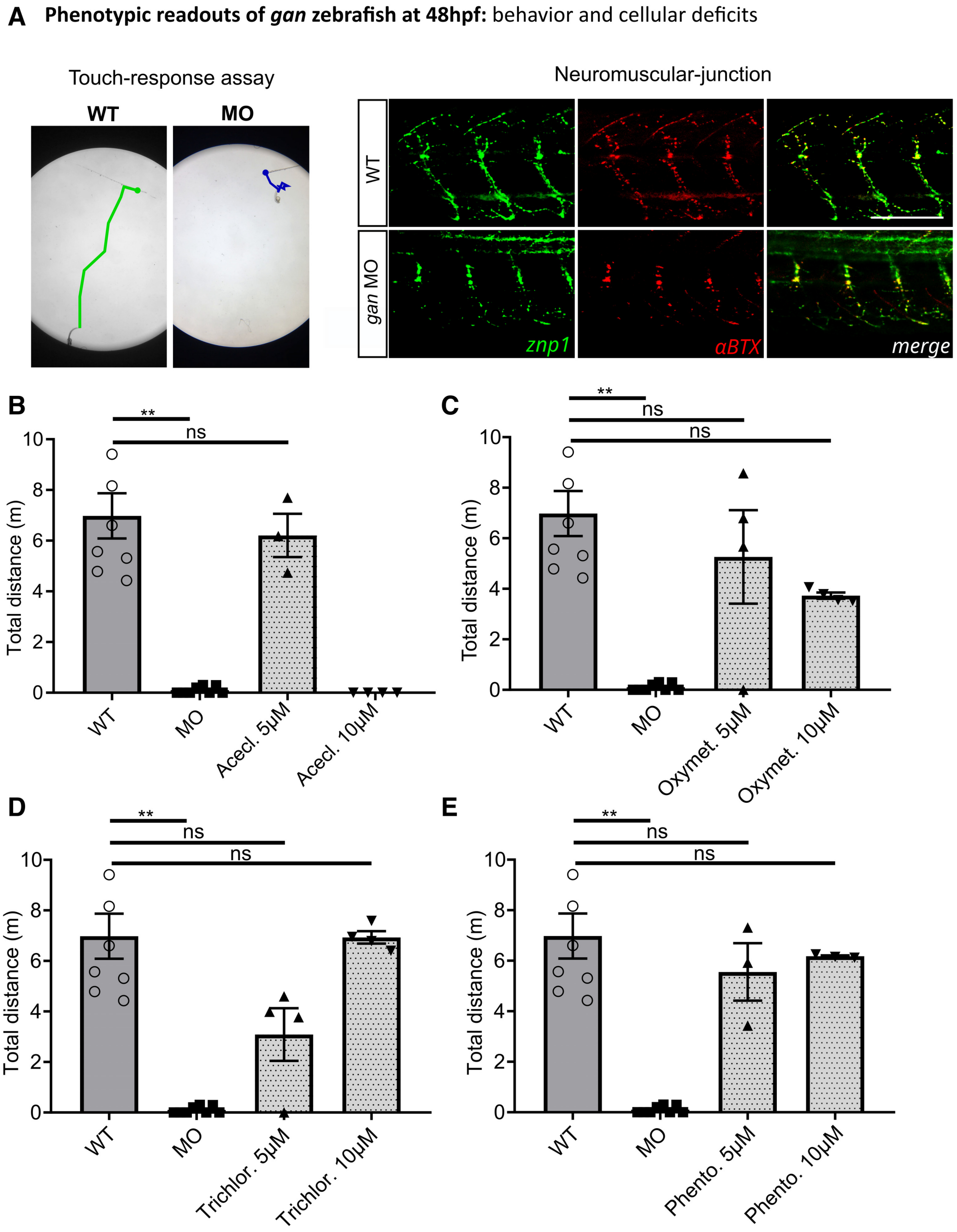

Figure EV6. Beneficial effect of the Hits upon treatment at symptomatic stage.

A Phenotype of *gan* MO-injected embryos at 48 hpf, with deficits in touch-responsiveness and impairment of axons and neuromuscular junctions (znp1: green; αBTX: α-bungarotoxin: red). Scale bar represents a length of 100 µm.

B–E Restoration of motility of 5-day-old *gan* larvae, when treated from 48 hpf with Aceclidine Hydrochloride (Acecl.) (B), Oxymetazoline Hydrochloride (Oxymet.) (C), Trichlormethiazide (Trichlor.) (D) and Phentolamine Hydrochloride (Phento.) (E). Hits were applied from 5 to 30 µM concentrations, with daily bath changes; data are not provided for the higher doses (20 and 30 µM) due to toxicity. Results show the distance traveled during 1 h by *gan* MO-injected larvae, treated with either DMSO or Hits at 5 and 10 µM. In the absence of normality of distribution of the data, a nonparametric Kruskal–Wallis test is applied; means ± SEM are represented. $^{**}P \leq 0.01$.

 © 2023 The Authors
