# Supplementary material for: A multilevel screening pipeline in zebrafish identifies therapeutic drugs for GAN
Source: EMBO Mol Med. 2023 May 5;15(7):e16267. doi: 10.15252/emmm.202216267 (PMC10331585; doi:10.15252/emmm.202216267)
Supplement: Supplementary file 3 — Movie EV1 [file EMMM-15-e16267-s004.zip › Movie_EV1_legend.pdf]

Movie_EV1 Legend: Representative recording of the motility of 5day-old larvae in 96 well-plate, showing an impairment in the spontaneous locomotion of *gan* zebrafish models (MO-injected and deletion line), in comparison to non-injected WT animals.
