# Supplementary material for: A multilevel screening pipeline in zebrafish identifies therapeutic drugs for GAN
Source: EMBO Mol Med. 2023 May 5;15(7):e16267. doi: 10.15252/emmm.202216267 (PMC10331585; doi:10.15252/emmm.202216267)
Supplement: Supplementary file 4 — Movie EV2 [file EMMM-15-e16267-s001.zip › Movie_EV2_legend.pdf]

Movie_EV2 Legend: Representative movie of the touch-response assay performed at 48hpf. The *gan*-MO embryo does not respond to the mechanosensory stimulation, while WT embryo rapidly swims away to escape from the stimuli.
