# Supplementary material for: A multilevel screening pipeline in zebrafish identifies therapeutic drugs for GAN
Source: EMBO Mol Med. 2023 May 5;15(7):e16267. doi: 10.15252/emmm.202216267 (PMC10331585; doi:10.15252/emmm.202216267)
Supplement: Supplementary file 5 — PDF+ [file EMMM-15-e16267-s002.pdf]

Article

TRANSPARENT PROCESS OPEN ACCESS

EMBO Molecular Medicine

A multilevel screening pipeline in zebrafish identifies therapeutic drugs for GAN

Léa Lescouzères[1], Cédric Hassen-Khodja[2], Anaïs Baudot[3], Benoît Bordignon[2] & Pascale Bomont[1,*]

Abstract

Giant axonal neuropathy (GAN) is a fatal neurodegenerative disorder for which there is currently no treatment. Affecting the nervous system, GAN starts in infancy with motor deficits that rapidly evolve toward total loss of ambulation. Using the *gan* zebrafish model that reproduces the loss of motility as seen in patients, we conducted the first pharmacological screening for the GAN pathology. Here, we established a multilevel pipeline to identify small molecules restoring both the physiological and the cellular deficits in GAN. We combined behavioral, *in silico*, and high-content imaging analyses to refine our Hits to five drugs restoring locomotion, axonal outgrowth, and stabilizing neuromuscular junctions in the *gan* zebrafish. The postsynaptic nature of the drug's cellular targets provides direct evidence for the pivotal role the neuromuscular junction holds in the restoration of motility. Our results identify the first drug candidates that can now be integrated in a repositioning approach to fasten therapy for the GAN disease. Moreover, we anticipate both our methodological development and the identified hits to be of benefit to other neuromuscular diseases.

Keywords giant axonal neuropathy; neuromuscular junction; pharmacological screening; therapy; zebrafish model
Subject Category Neuroscience
DOI 10.15252/emmm.202216267 | Received 11 May 2022 | Revised 30 March 2023 | Accepted 5 April 2023 | Published online 5 May 2023
EMBO Mol Med (2023) 15: e16267

Introduction

Neuromuscular disorders (NMDs) encompass a large spectrum of diseases with more than 150 distinct types described. The most common feature of NMDs is muscle weakness, caused by injury, dysfunction of peripheral nerves, or muscles. NMD classification is based on which neuromuscular unit is primarily affected, that is, the motor/sensory neuron, the peripheral nerves, the skeletal muscle, or the neuromuscular junction (NMJ; Morrison & Griffin, 2009; Michel & Collins, 2020). Within the group of peripheral neuropathies, our group studies giant axonal neuropathy (GAN, OMIM#256850; Asbury *et al*, 1972; Berg *et al*, 1972; Kuhlenbäumer *et al*, 2020), a rare disease that shares clinical and histopathological features with some forms of the most prevalent condition, namely Charcot–Marie–Tooth (CMT) diseases (Juneja *et al*, 2019; Laurá *et al*, 2019). Extremely severe in its classical form, GAN is detected during infancy and is fatal in young adults. The course of the disease starts with difficulty in walking, decrease in deep tendon reflexes, areflexia and amyotrophy, and evolves toward a total loss of deep and superficial sensitivity and ambulation in teens. While some rare milder forms of GAN do not spread to the central nervous system, the classical forms cause a myriad of symptoms in young adults, encompassing nystagmus, dysarthria, intellectual disability, ataxia, and epileptic seizures.

Our group identified the genetic locus of the recessive disease GAN (Cavalier *et al*, 2000) and subsequently the *GAN* gene (Bomont *et al*, 2000), which encodes for gigaxonin, an adaptor of a Cul3-E3 ubiquitin ligase complex (Lescouzères & Bomont, 2020). Developing two diagnostic tools for GAN, that is, genetic mutation (Bomont *et al*, 2000) and abundance of gigaxonin (Boizot *et al*, 2014), we permitted the identification by our and other laboratories of 75 distinct mutations scattered along the entire *GAN* gene. These encompass a wide range of mutation types (60% missense, 17.4% nonsense mutations, 16% deletions/insertions, and 6.6% splice mutations), for which we demonstrated a generalized instability of the mutated-gigaxonin (Boizot *et al*, 2014; Lescouzères & Bomont, 2020).

In agreement with the broad spreading of symptoms across the nervous system and the wide aggregation of Intermediate Filaments (IFs) throughout GAN patients' body, the gigaxonin-E3 ligase was found to play pivotal roles in neuronal and cytoskeleton homeostasis (Lescouzères & Bomont, 2020). Indeed, combining the use of primary fibroblasts from patients (Bomont & Koenig, 2003; Cleveland *et al*, 2009; Mahammad *et al*, 2013), and GAN knockout mice (GAN KO$^{del\text{-}ex1}$ and GAN KO$^{del\text{-}ex3\text{-}5}$; Dequen *et al*, 2008; Ganay *et al*, 2011), we and others provided crucial insights into gigaxonin's role in controlling the cytoskeletal IF family turnover (Mahammad *et al*, 2013; Bomont, 2016) and in regulating the autophagy pathway, through the ubiquitin-dependent degradation of the ATG16L1

1 ERC Team, NeuroMyoGene Inesitute – Now PGNM, Inserm U1315, CNRS UMR5261, University of Lyon 1, Lyon, France
2 Montpellier Ressources Imagerie, BioCampus, University of Montpellier, CNRS, INSERM, Montpellier, France
3 Aix Marseille Univ, INSERM, MMG, Marseille Medical Genetics, CNRS, Marseille, France
*Corresponding author. Tel: +33 4 26 68 82 54; E-mail: pascale.bomont@inserm.fr

© 2023 The Authors. Published under the terms of the CC BY 4.0 license.

protein (Bomont, 2019; Scrivo *et al*, 2019). In addition, the recent development of *gan* zebrafish models by our laboratory permitted to unveil gigaxonin physiological functions. Using both transient and stable knockout approaches, we revealed that gigaxonin is required to sustain motility in zebrafish, by controlling motor neuron specification and axonal outgrowth (Arribat *et al*, 2019). At the molecular level, we demonstrated that gigaxonin controls the turnover of the Ptch receptor, to positively modulate Sonic Hedgehog (Shh) pathway activity, one of the key developmental machinery sustaining neuron and muscle fate in vertebrates (Jessell, 2000; Te Kronnie & Reggiani, 2002). In the absence of gigaxonin in zebrafish, Shh activity is reduced, leading to an impairment of motor neuron stability and somitogenesis, and the abolishment of neuromuscular junction formation and denervation.

In the field of NMDs, most therapeutic efforts have focused on gene therapy (Juneja *et al*, 2019; Ravi *et al*, 2019), and this approach is being considered for GAN with an ongoing phase I clinical trial using intrathecal administration of an AAV9-GAN product (ClinicalTrials.gov Id: NCT02362438, 2015). Alternatively, the development of nonmammalian models promoted the rise of high-throughput screenings of pharmacological compounds in the last 10 years (Giacomotto & Ségalat, 2010; Patton *et al*, 2021). In particular, the zebrafish (*Danio rerio*) model provides a strong therapeutic potential for neurologic diseases and has been proposed as the best alternative to mammalian screening for phenotype-based *in vivo* drug discovery (MacRae & Peterson, 2015). The reasons for this include the high conservation of genes and protein similarity between zebrafish and human, the external fertilization and rapid development through well-defined stages, facilitating the observation and experimental manipulation of embryos. Moreover, the statistical power of the screens is ensured by the high fecundity of progenitors. Zebrafish embryos' small size allows them to be placed in 96-well plates and to be easily treated by balneation. Finally, the transparency of the embryos facilitates investigations at the physiological level within tissues and is well-documented for neuronal, muscle, and neuromuscular systems (Pappalardo *et al*, 2013). To date, more than 65 small-molecule screens in zebrafish have been reported in the literature (MacRae & Peterson, 2015). Overall, this approach led to the identification of about 10 drug candidates, which are currently tested in clinical trials in various fields of biomedical science (Patton *et al*, 2021). Among them, the neuroleptic compound pimozide, which is under Phase 2 clinical trial (ClinicalTrials.gov Id: NCT03272503, 2017), was first identified from a high-throughput screen in a zebrafish model for ALS (Patten *et al*, 2017), hence confirming the great value of zebrafish in clinical translation.

While we and others have revealed the poor value of the GAN KO mouse models for preclinical studies, presumably because of genetic compensations due to the activation of the nonsense-induced transcriptional compensation (NITC) pathway (El-Brolosy *et al*, 2019; Ma *et al*, 2019), we generated robust *gan* zebrafish models with high relevance, penetrance, and robustness in regard to the human pathology (Arribat *et al*, 2019). Indeed, we showed that 80% of gigaxonin-depleted animals exhibit a total loss of motility, with the remaining moving with decreased speed and over shorter distances. We further demonstrated the molecular pathway by which gigaxonin depletion impairs motor neuron stability, abolishes neuromuscular junction formation, resulting in denervation. Overall, the *gan* zebrafish model is the first to reproduce the loss of ambulation and the severity of symptoms seen in patients, hence providing the first hope to design effective therapeutic strategies for the fatal GAN disease.

In this study, we took advantage of our robust *gan* zebrafish model to perform a drug screening, in a strategy of repurposing of small molecules. Toward this aim, we developed an *in vivo* behavioral-based screening strategy, by miniaturizing an assay to quantify and score the locomotor deficit in zebrafish. The screening of the Prestwick Chemical Library®, containing 1,280 small molecules, evidenced 59 common molecules between the two different *gan* zebrafish models. Then, we conducted an *in silico* system biology approach to identify the different pharmacological families of Hits and pinpointed recurrence, allowing us to further reduce our candidate to 16 drugs. We subsequently developed a novel imaging-based filtering method (Lescouzères *et al*, 2022) to identify Hits rescuing the NMJ and axonal deficits in the *gan* zebrafish. Overall, we established a stepwise methodology to pinpoint two specific pharmacological classes (muscarinic antagonist and α-adrenergic agonist), whose actions converge in the stabilization of the NMJ units. Finally, we show that the effect of the small molecules on NMJ maintenance is directly mediated via their actions on cholinergic and adrenergic receptors. Our results suggest that α-adrenergic and muscarinic receptor impact NMJ synaptic organization and their therapeutic benefit in GAN may be through morphological restoration of the NMJ to compensate neurogenesis defects in the disease.

Results

Miniaturization of the loss of motility in *gan* zebrafish and scoring of its robustness for drug screening

To evaluate the potential of the *gan* zebrafish model as a suitable biological source for pharmacological screening, we focused on the robust and meaningful physiological readout test, that is, the loss of locomotion at the behavioral level. As described in our previous study, depletion of gigaxonin using a transient approach (by injecting morpholino antisense oligonucleotides, MO) recapitulates the severe locomotor symptoms seen in patients (Arribat *et al*, 2019; Fig 1). To note, the transient model was preferred over the stable KO line because it was obtained first and because it shows better penetrance and overall severity, presumably due to the activation of the NITC pathway in the genetic line. Thus, repression of gigaxonin in zebrafish induces several defects (Fig 1A), with a decreased production of motor neurons, a shortening of axons (Fig 1B and C), and a decreased formation of neuromuscular junctions (Fig 1C), which induce a severe locomotion deficit (Fig 1D and E). To miniaturize this behavioral readout test, we monitored the spontaneous locomotion of 5 dpf larvae in a 96-well-plate format. We recapitulated our previous study, with a complete loss of locomotion in 79.2% of *gan* morpholino-injected (MO) animals, the remaining presenting a dramatic decrease (85%) in the total distance traveled within an hour (Fig 1D and E, and associated Movie EV1). By far, the most common format to conduct chemical screen using live zebrafish is the 96-well plate (Peterson *et al*, 2000; Best & Alderton, 2008; Rennekamp & Peterson, 2015), simply by adding small amounts of compounds

© 2023 The Authors

directly to the fish water. To validate our phenotypic *in vivo* read-out for extensive drug screening, we assessed the robustness of the assay through assignment of Z'-factor quality metrics. Z'-factor is a dimensionless number that discriminates the positive (i.e., noninjected wild-type (WT)) and negative (i.e., WT injected with MO) controls, used to quantify the range of motility measurements within the test. Z'-factor analysis of our data was satisfactory (Z' = 0.57), within the range of other similar zebrafish screens (from −7.1 to 0.78; Walker *et al*, 2012), ensuring maximized throughput and reproducibility. Subsequently, we established experimental conditions, including: (i) an evaluation of potential plate effects (timing of analysis and position on the plate); (ii) different timing and methods of dechorionation; and (iii) the number of fish tested per drugs. This preliminary work allowed us to define an experimental scheme, which is described in Fig EV1. Thus, we designed the first GAN disease-relevant phenotypic assay for a drug screening with the aim to screen and identify small molecules able to rescue the loss of locomotion in 5 dpf treated *gan* morphants, compared with noninjected WT larvae.

Pharmacological rescue of locomotion defects in the *gan* morphants

To identify small molecules restoring locomotion in the *gan* zebrafish model, we screened 1,280 drugs from the Prestwick Chemical Library®. This library comprises 95% of FDA-approved drug, in agreement with a repositioning strategy. At first intention, drugs were tested at a single concentration of 10 µM, which corresponds to the compromised concentration in the field between activity and toxicity for many compounds in zebrafish (Rennekamp & Peterson, 2015). To quantify both the potency of individual drugs in restoring locomotion and the penetrance of this effect, we assigned a behavioral scoring relying on a z-score mean value of four biological replicates, which measures the total distance traveled over an hour (Fig 2A). We set up

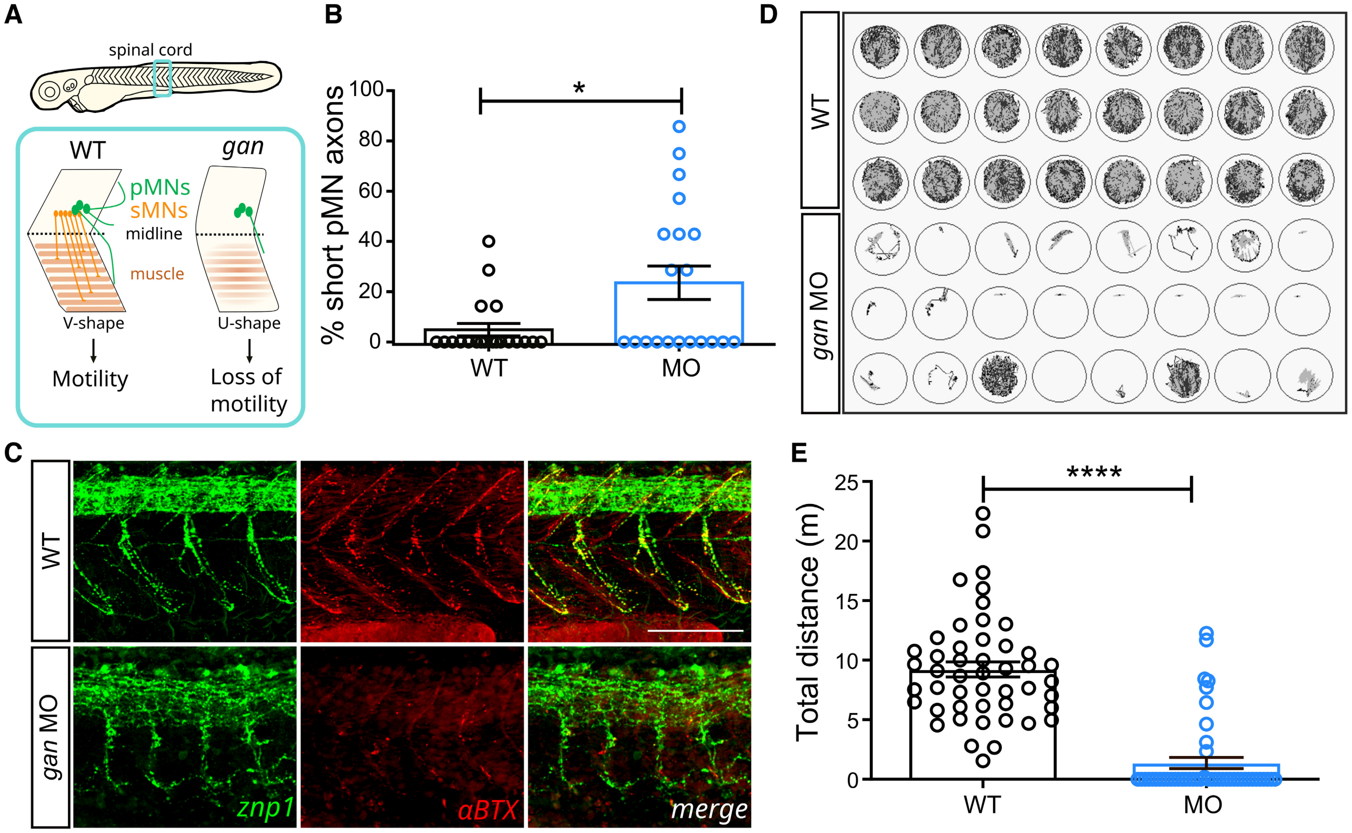

Figure 1. The *gan* zebrafish mimics the loss of motility described in GAN patients.

A Schematic of the behavioral and cellular defects described in the *gan* zebrafish model. Underlying the loss of motility in the *gan* zebrafish, the architecture of the spinal cord is remodeled with shortening and/or absence of MN axons (in green, primary MNs; in red, secondary MNs) and loss of neuromuscular junctions (NMJ). The neuromuscular phenotype is accompanied by a change in the shape of myofibers, which adapt a "U-shape" instead of a normal "V-shape."

B The percentage of shorter pMN axons (< 70 µm) per fish is significantly higher in *gan* morphants ($n = 20$) than in noninjected WT ($n = 20$) at 48 hpf.

C Representative images for the neuromuscular junctions (znp1: green; αBTX: α-bungarotoxin: red) in WT and *gan* morphants at 48 hpf. Note the shorter pMN axons and sparse AChR clusters in *gan* morphants.

D Representation of the cumulative tracking of the spontaneous locomotion of 5-day-old larvae for 1 h, in noninjected and MO-injected animals.

E Quantitative measures of the traveled distance (m: meter) show total loss of locomotion in 79.2% of *gan* morphant; $n = 48$ (WT), $n = 48$ (MO).

Data information: (B, E) Each dot represents individual larvae; $^*P < 0.05$, $^{****}P < 0.0001$. In the absence of normality of distribution of the data, a nonparametric Mann–Whitney *U* test was applied. Data are represented as means ± SEM. (C) Scale bar represents a length of 100 µm.

© 2023 The Authors

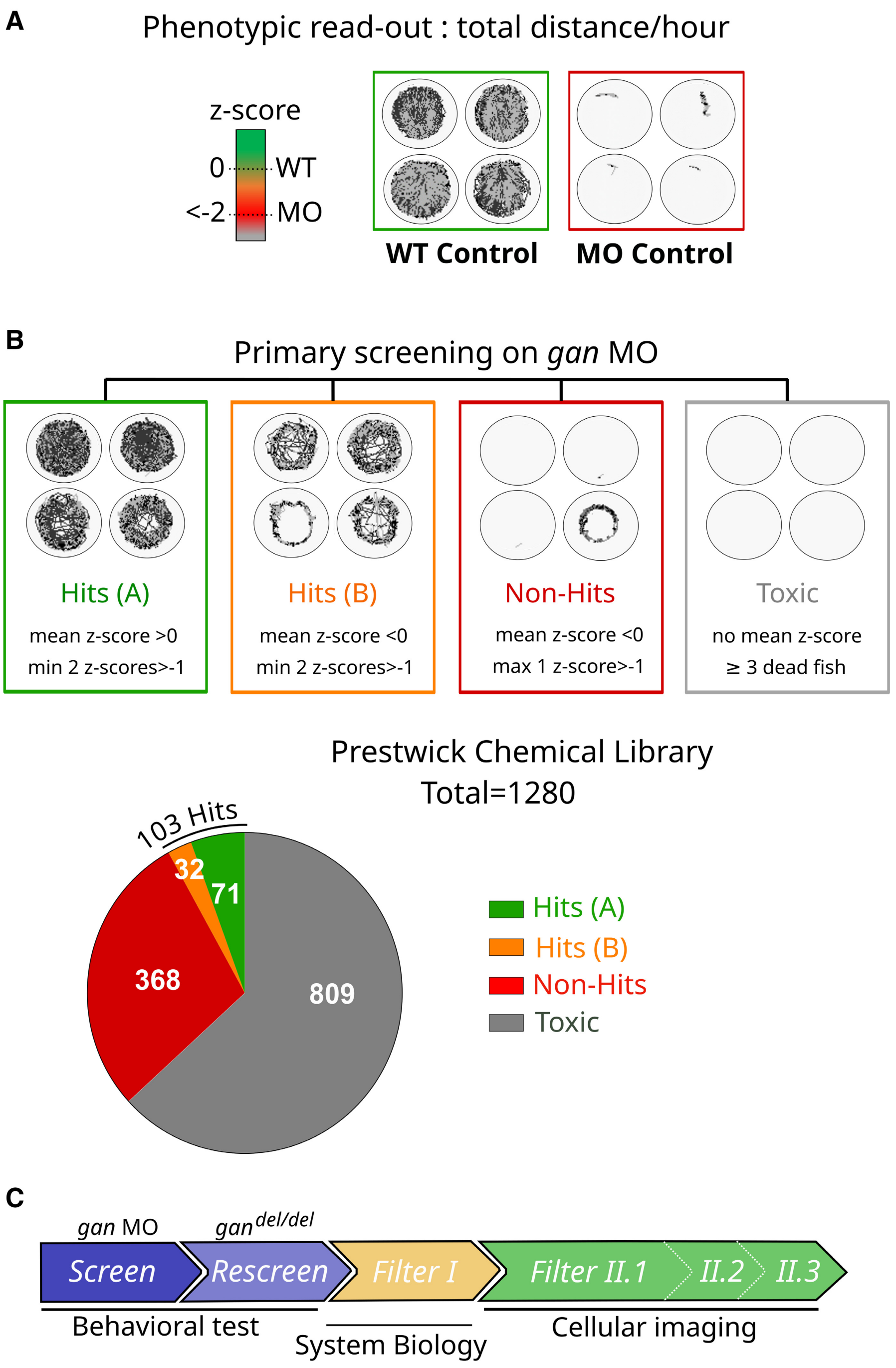

Figure 2. Development of a screening strategy in zebrafish to score, categorize, and refine candidates to 103 hits.

A Efficacy of Hits is determined by the establishment of a z-score scale, ranging from 0 (noninjected "WT Control," green) to negative values < -2 (*gan* morpholino-injected "MO Control," red) after normalization with WT values. Representative pictures of video tracking show the total distance traveled over 1 h by quadruplicate WT and MO fish at 5 dpf.

B Primary screening on *gan* morphants identifies four chemicals classes depending on their mean z-scores and individual values. Hits restore motility in > 2/4 animals (min 2 individual z-scores > -1), while non-Hits may have an effect only on 1/4 animals (max 1 z-score > -1) and toxic compounds induce death in ≥ 3 fish. Hits are further categorized in two groups A and B, accordingly to the penetrance of the phenotype: B Hits exhibit a negative mean z-scores due to 1–2 fish with low values, while A Hits are penetrant with positive mean z-scores. Screening of the 1,280 Prestwick Chemical compounds identifies 809 Toxic and 368 Non-Hits molecules, and a total of 103 Hits with 71 A Hits and 32 B Hits.

C Summary of the screening strategy workflow for the GAN disease, encompassing behavioral tests in the two *gan* zebrafish models (Screen and Rescreen), computational approach using System Biology (Filter I) and a novel imaging screening methodology at the cellular level in zebrafish (Filters II 1–3).

© 2023 The Authors

a locomotion score scale ranging from null values (Z-score = 0, noninjected WT animals) to negative values (Z-score < −2) indicative of a lack of locomotion, characteristic of untreated larvae injected with the *gan* MO. Then, the locomotion behavior of larvae treated with the 1,280 drugs was scored (full set of data presented in Fig EV2). We then categorized the responses in four different classes of compounds (Fig 2B). Toxic compounds have no associated mean z-scores because ≥ 3/4 of the fish larvae die after treatment; non-Hits compounds are not efficient on at least 3/4 of larvae injected with *gan* MO. Finally, positive Hits can be classified as (A) compounds associated with positive mean z-scores highly penetrant (≥ 2/4 animals injected with *gan* MO with individual z-scores strictly above −1) and (B) compounds associated with strong individual z-scores (≥ 2/4 animals injected with *gan* MO with individual z-scores strictly above −1) but presenting a negative mean z-score.

The dot-plot representation of individual and mean z-scores presents the Hits in the most statistically powerful category (Hits A; $n = 71$, Figs 2B and 3A) with mean z-scores close to noninjected WT values and the second subtype (Hits B; $n = 32$, Figs 2B and 3B) less penetrant with at least one fish per compound associated with a high negative z-score. Overall, our behavioral readout test identified 103 Hits (8.04% of the library) able to restore motility in most of the treated *gan* morphants (Fig 2B).

To increase the relevance of our approach, we defined a strategy (Fig 2C) combining a behavioral rescreening on the other *gan* zebrafish model ("Rescreen"), a system biology approach ("Filter I") and "high-content" (e.g., confocal imaging-based) whole-organism cellular analysis ("Filter II") to evaluate the restoration of the specific NMJ-associated phenotypes, previously identified in the *gan* model.

Reduction in candidates using the genetic *gan*$^{del/del}$ CRISPR model

To ensure the reproducibility of our results across different gigaxonin-depleted zebrafish models, the same strategy was adopted to rescreen the 103 Hits compounds in the *gan* knockout zebrafish line ("Rescreen," detailed in Appendix Table S1). As previously described, the *gan*$^{del/del}$ zebrafish also presents severe defects in spontaneous locomotion, with a significant reduction in the total distance traveled within an hour in 80% of the mutants (Arribat *et al*, 2019; see Movie EV1).

The purpose of the "Rescreen" was to take advantage of the genetic *gan*$^{del/del}$ CRISPR model to reinforce the robustness of the hits identified in the drug screening, and possibly eliminate false positives. Here, we could not apply the same analytic method used for the screening, due to the overrepresentation of positive events within the experiment. Indeed, all molecules were selected to have a beneficial effect on motility, hence unbalancing the negative values represented within the plate. Therefore, we analyzed the Normal Percentage Activation (NPA, detailed in Materials and Methods section), which was previously described as the most appropriate way to score sample values with positive effect and high variability. Thus, we normalized the total distance traveled by treated *gan* KO versus WT larvae and established a scale from 0 to 20% (*gan* KO) to 100% (WT) Normal Percentage Activation, to provide the most appropriate scoring method in identifying subpopulation of compounds among efficient Hit sets. With this methodology, we validated 59 Hit compounds able to rescue in average at least 50% of the locomotion in the treated *gan* KO zebrafish model, and significantly different from untreated *gan* deletion mutants (0–20%; Fig 4). Thus, the Rescreen permitted to reduce the number of Hits compounds common to both *gan* zebrafish models, to 59 (Appendix Table S1 for details).

System biology approach identifies three functional groups and recurrence within Hits

To better refine our Hits, we performed an additional selection step, using System Biology (Filter I). The screened Prestwick Chemical Library® has a high chemical and pharmacological diversity. We first classified the 59 common Hits compounds according to their pharmacological class annotations, as described in the library database (Fig EV3). This analysis evidenced an enrichment of the common Hits in the Cardiovascular system (from 17.6; 4% to 23.73%), in CNS (from 17.65 to 18.64%), and to a lesser extent the Neuromuscular system. Conversely, this approach ruled out several pharmacological categories, including Gastroenterology and Infectiology.

At this decision-making stage, a common limit for such phenotype-based screenings is the difficulty to further determine the mechanisms of action of the Hits compounds (Schenone *et al*, 2013), as the approach is meant to identify disease-modifying drugs regardless of the knowledge of their molecular targets. Therefore, to enrich our strategy, we conducted an *in silico* system biology approach (Filter I) to identify the targets of our Hits and predict potential mechanisms of action (Fig 5).

At first, a Drug-Target list was constructed with known Drug-Target Interaction data extracted from DrugBank for the 59 common Hits. This approach identified 95 poly-pharmacological targets for 44 common Hits. However, the database does not report any known targets for 15 of the 59 common Hits. The latter were classified as Unknown Hits and kept for additional filtering stages (Filter II).

Next, using the STRING v.11 database, we retrieved the interactions between the targets to extract functional associations, that is, links between targeted proteins that participate jointly in specific biological functions. This analysis revealed a network composed of 89 nodes and 427 edges. The Protein–Protein Interaction enrichment means that targeted proteins have more interactions than expected for a random set of proteins. We selected target protein interactions with high confidence (0.7) to construct our network with Cytoscape, hence decreasing the number of edges to 92 (Fig 5). The analysis of the obtained network revealed two important characteristics. First, we identified strong enriched interactions (P-value $< 1.0e^{-16}$), as a result of a functional link between targets against which several Hits are directed. The network is organized in three main clusters targeting cholinergic agents, adrenergic agents, and carbonic anhydrase (CA) agents. Second, beyond protein interaction, the analysis of the drug-target networks revealed overrepresented targets, that is, targets that are recognized by multiple Hits. We defined a cutoff of three recurrences to identify the recurrent targets (Fig 5). Interestingly, the 17 recurrent targets are mostly G-coupled receptors (11/17). Overall, our *in silico* analyses pinpointed the clustering of Hits into three functional groups and 16 Hits sharing targets with high recurrence (Fig 5). These 16 hits, named Favorite Hits, were selected for a cellular imaging-based filtering in the *gan* zebrafish.

© 2023 The Authors

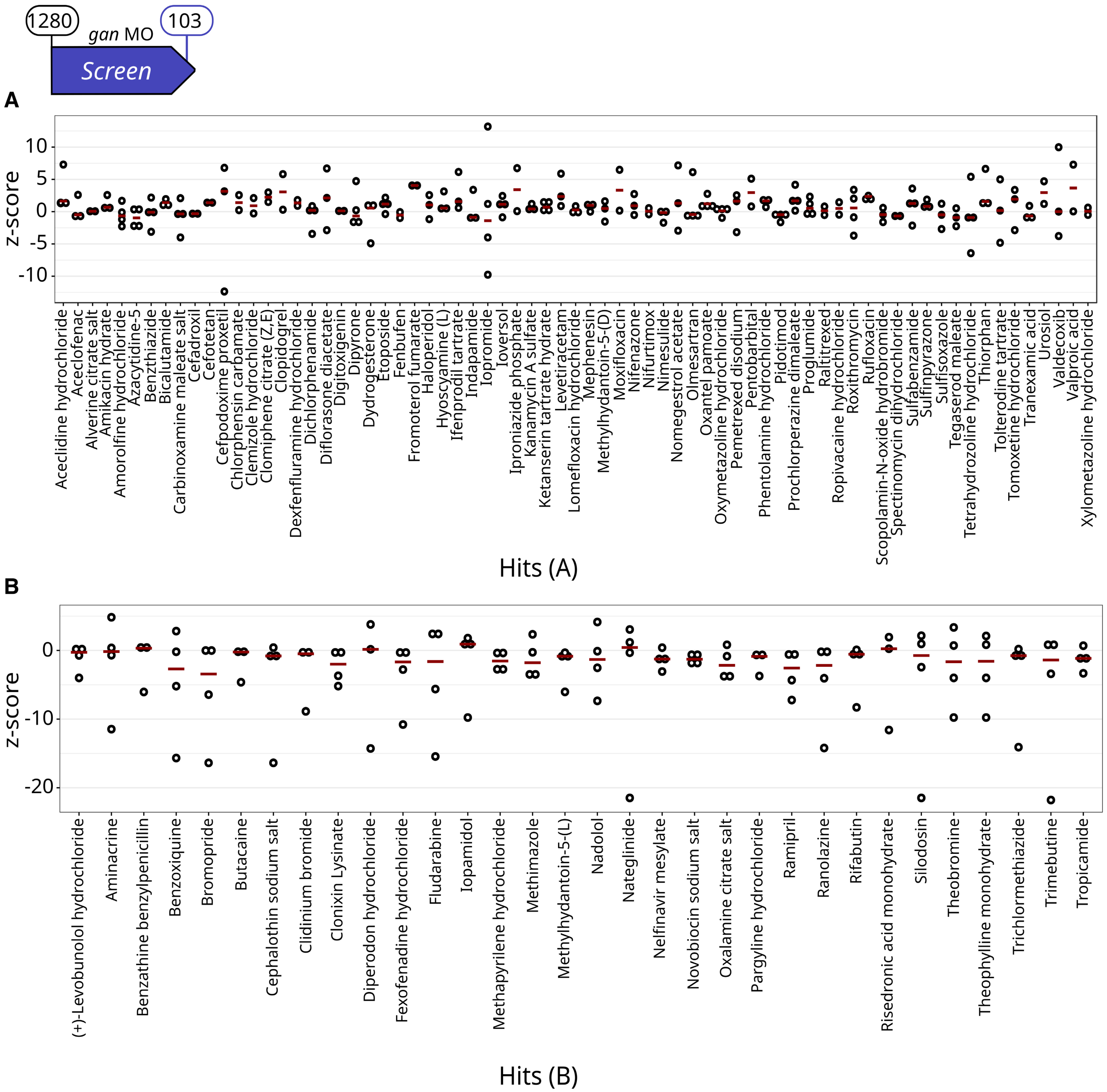

Figure 3. Primary Screen identifies 103 compounds restoring locomotion defects in the *gan* MO-injected zebrafish.

A, B Single z-scores, comparing the total distance traveled by quadruplicate *gan* fish over 1-h period are plotted for the 71 A Hits (A) and the 32 B Hits (B).

Data information: For each compound, circles represent individual larvae ($n = 4$ per drug) and red lines show the median z-scores (see Fig EV2 for results on the whole library).

Development of a novel quantitative imaging methodology refines Hits restoring the stability of the neuromuscular junction

To increase the stringency of the selection of our Hits, we designed an additional filtering method (Filter II), based on the cellular deficits identified in the *gan* zebrafish models (Fig 1). To test whether the selected compounds exert their positive effect through the stabilization of the axonal length and NMJ, we designed a cellular-based method to quantify these parameters in noninjected WT, nontreated, or treated *gan* morphants at 48 hpf. This was achieved with the development of a novel high-content pipeline for image acquisition and analysis, using the Opera Phenix™ High Content Screening System confocal and the Harmony software (methodology published in Lescouzères *et al*, 2022). Briefly, pMN axons and

 © 2023 The Authors

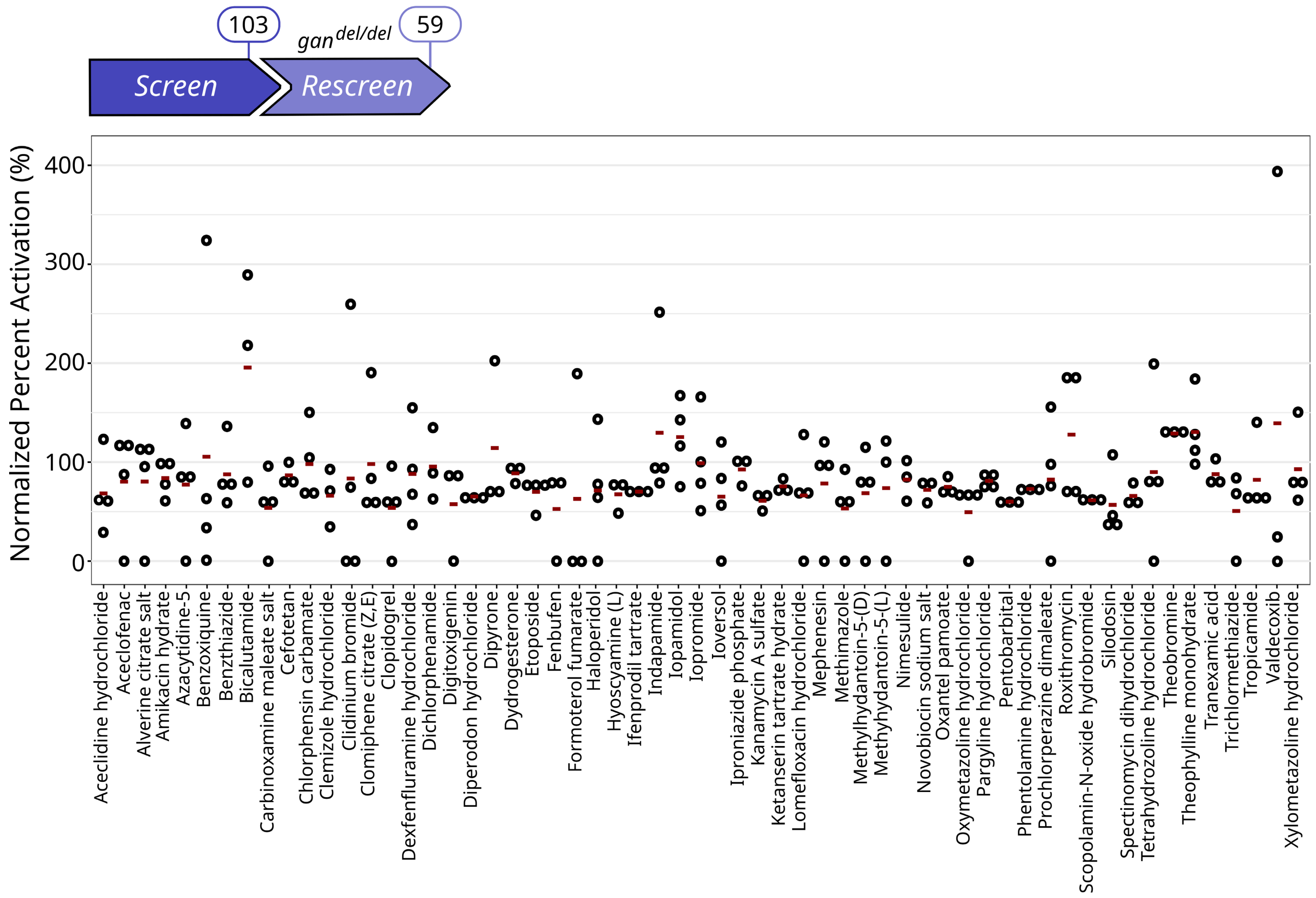

Figure 4. Rescreen identifies 59 compounds restoring locomotion defects in the *gan*$^{del/del}$ model.

The 103 Hits identified in the *gan* MO-injected fish were further tested in the *gan* CRISPR zebrafish line at 5 dpf. Due to the great imbalance toward positive results (all 103 compounds are Hits), the statistical analysis used a normalization of Percent Activation (%). Readout signals were calibrated and normalized to a 0–100% effect scale, in which 0% represents no effect and 100% corresponds to the locomotion activity of WT fish (See details in Appendix Table S1). Here, the cutoff for the restoration of motility was assigned to > 50% and identifies 59 Hits. Data information: For each drug, circles represent individual larvae ($n = 4$) and red lines show the medians for the Normalized Percent Activation.

neuromuscular synapses of 48 hpf-old embryos were labeled with synaptotagmin marker (znp1, axons) and alpha-bungarotoxin (αBTX, postsynaptic AChRs), and embryos were individually placed in 96-well plates in a lateral position (Fig 6A). To detect the neuromuscular system in the entire animal, we created an automatized protocol to (i) first automatically locate single zebrafish larvae in the well, (ii) create a global image (see details in Materials and Methods section and Lescouzères *et al*, 2022) at low magnification (5×); and (iii) acquire selected regions with segmentation at 20× (see picture in Fig 6A).The first regionalization filter (1) was developed to enable the automatic detection and quantification of single AChRs clusters (Fig 6B, left panel). The second regionalization filter (2) was generated for the evaluation of the percentage of co-localization area between these presynaptic NMJ components and axonal region (Fig 6B, right panel). Next, we established a method to automatically demarcate the dorsal spinal cord as a specific region of interest (Fig 6C), which allowed us to quantify axonal length by individualizing each axon (3, right panel). Thanks to the combination of the three filters, we succeeded in quantifying the effect of our Hits in restoring NMJ synapses (Fig 6D1′–2′) and axonal length (Fig 6D3′) in the *gan* zebrafish.

Robustly, our analysis shows that overall, the 16 favorite Hits can restore NMJ structure at the three cellular levels in *gan* morphants: They improve AChR clustering (Fig 7A), rescue axonal outgrowth (Fig 7B), and increase the co-localization of axonal terminals with AChR clusters (Fig 7C). Additionally, we integrated the Unknow Hits from the System biology analysis and also showed a significant improvement of postsynaptic AChR clustering number and percentage of co-localization area between the axonal and postsynaptic NMJ components in the *gan* morphants. Raw data for all tested chemicals are provided in Appendix Tables S1–S4. Taken together, the restoration of the AChR clustering and area of co-localization with axonal projection is a hallmark of our Hits (Favorite and Unknown) in rescuing synaptic contacts in the *gan* morphants. While these data provide the statistical significance of entire groups (Favorites, Unknown versus noninjected, and MO-injected embryos), we evaluated the specific scores of individual Hits, to rank their beneficial value. Toward this aim, we integrated

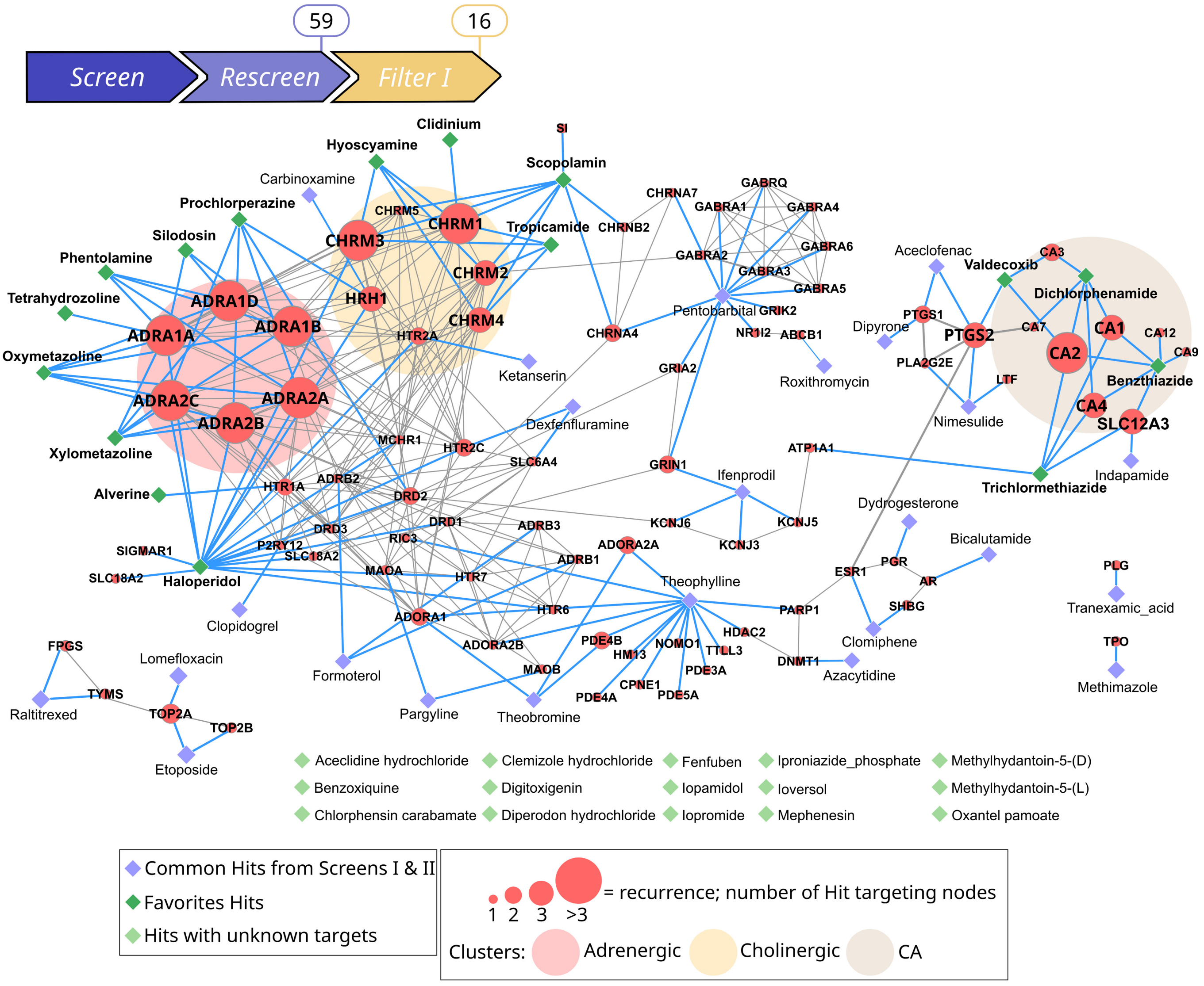

Figure 5. Filter I: Computational network analysis of the Hit targets identifies three functional groups and high recurrence, and refines candidates to 16 Hits.

Drug–Target list was constructed with known Drug–Target Interaction data extracted from DrugBank for the 59 candidate Hits. Subsequently, gene network of the 89 targets and interactions of the corresponding proteins were obtained from the STRING database (non-interacting protein are removed here). Diamond-shaped nodes correspond to drugs compounds as annotated in the DrugBank database and red nodes correspond to their targets (gene names presented here). Isolated diamonds (in light green) correspond to the 15 drugs with unknown targets. Edges corresponding to Target–Target interactions from STRING are indicated as solid gray lines, and Drug–Target interactions from DrugBank in blue lines. Recurrence analysis pinpoints a clustering of Hits into three functional groups with high recurrence, as depicted with ellipses: Adrenergic (light pink), Cholinergic (light yellow), CA (light brown). Target node size is scaled according to the number of drugs targeting the node and reflects recurrence. Recurrence of > 3 refines favorite drugs to 16 Hits (dark green diamond), represented in the three functional groups.

the results of the three parameters for each Hit (Fig 7D and illustrations for the drug Aceclidine Hydrochloride in Fig 7E) and calculated its associated z-score compared with noninjected WT embryos. This analysis, depicted in a 3-D scatterplot (the detailed coordinates of Fig 7D are provided in Appendix Table S5) reveals that only five drugs are able to rescue the common parameters regarding NMJ (parameters (1) and (2) in Fig 7). These five drugs are divided into the three functional categories described above: two Adrenergic agents (15—Oxymetazoline Hydrochloride, 16—Phentolamine hydrochloride), one CA agent (19—Trichlormethiazide), and two Hits with Unknown target (1—Aceclidine Hydrochloride, 7—Digitoxigenin). Interestingly, the Aceclidine Hydrochloride, previously characterized as Unknown Hits according to the DrugBank database, turns out to be a well-described muscarinic AChR agonist (Ehlert *et al*, 1996). To analyze in greater details the activity of our five selected Hits, we examined their effect on the presynaptic compartment and conducted a dose-dependent treatment. First, we used the Synaptic Vesicle glycoprotein 2 (SV2) marker to show that its labeling intensity is significantly decreased in *gan* morphants compared with noninjected WT embryos at 48 hpf, and not rescued by the five Final Hits (Fig EV4A and C). Interestingly, the staining intensity of postsynaptic AChR receptors was found to be similar

 © 2023 The Authors

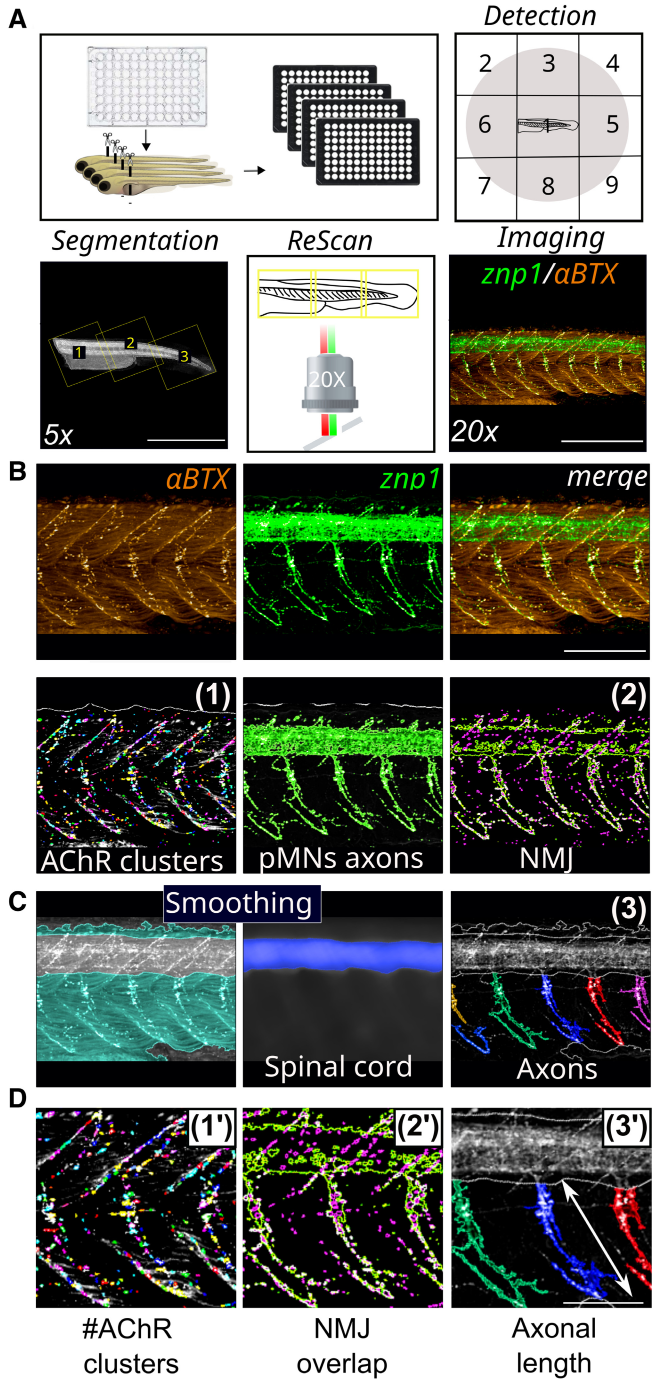

Figure 6. Filter II: Novel methodological analysis of automated imaging-screening.

- A Schematic overview of 48-h-old embryos for automated detection in 96 well-plates, ROI segmentation and Rescan imaging. Representative images of NMJ staining (znp1: green; αBTX: α-bungarotoxin: red) within the spinal cord of control larvae.
- B Representative images of the detection filters segmenting AChR clusters from α-bungarotoxin staining (1), pMNs axon area from znp1 staining and NMJ overlapping compounds (2) within the spinal cord of control larvae.
- C Representative image of the smoothing filter to extract the dorsal spinal cord staining as the specific region of interest to individualize axons (3).
- D Enlarged pictures of the parameters (1′–3′) for the quantification of NMJ and pMN axons.

Data information: Scale bar represents a length of 1 mm (A, 5×), 500 μm (A, 20×), 200 μm (B), 100 μm (D).

between noninjected and MO-injected embryos (Fig EV4B). On the contrary, the area of the postsynaptic clusters is significantly increased in the *gan* morphants (Fig EV4D). If drugs seem to be beneficial in rescuing their size, it does not reach the levels of noninjected WT values. Finally, as the initial screening was performed at a drug concentration of 10 μM, we conducted a dose–response analysis of the five selected Hits from 0.1 to 50 μM, using the motility assay (Fig EV5). With a general toxicity at 30 and 50 μM, the drugs are not significantly potent at low doses but present a dose-dependent effect for Phentolamine hydrochloride and Trichlormethiazide. Notably, we showed that all drugs exhibit maximal effect at 10 μM (and occasionally 5 μM), hence confirming the validity of our screening and filtering methodology at this concentration.

The benefits of Hits on locomotion are due to on-target effects at NMJ receptors and are also potent upon treatment at symptomatic stage

To determine whether the beneficial effects of Hits are due to their direct action on G-coupled receptors or to other (un)known mechanisms of action, we studied their efficiency following a pretreatment with receptor antagonists. Thus, we pretreated zebrafish embryos with the selective α1 adrenergic antagonist (Alfuzosin) or cholinergic antagonist (Curare), which both block synaptic transmission at NMJs and significantly decrease the total distance traveled by wild-type embryos (Fig 8A). Following the pretreatment with alpha-blocker, the addition of two adrenergic Hits (Oxymetazoline hydrochloride and Xylometazoline hydrochloride) failed to restore locomotion, indicating that the effects of our Hits are probably mediated by α1 receptors (Fig 8A and B). Interestingly, we observed a duality in the beneficial effect of cholinergic Hits after treatment with Curare. Aceclidine Hydrochloride remains effective in the presence of the antagonist, while Tropicamide tends to lose its effect. These results suggest that the effect of Tropicamide may be dependent on nicotinic AChR activity while Aceclidine Hydrochloride may have cholinergic-independent effect on locomotion (Fig 8B).

In this study, we showed that the five selected Hits induce robust recovery when administrated at early stage of development (8–48 hpf). To evaluate their efficacy upon treatment at symptomatic stage, we exposed *gan* MO-injected animals to drugs at 48 hpf, when both behavioral and cellular deficits are already present (Fig EV6A and Movie EV2). Analysis of increasing doses revealed that four Hits show efficacy in restoring the spontaneous locomotion of larvae at 5 and 10 μM, hence indicating clinical relevance of our pharmacological approach for future development for the GAN disease.

Discussion

Zebrafish is a model of choice for studying the nervous system at a physiological level and for phenotype-based *in vivo* drug

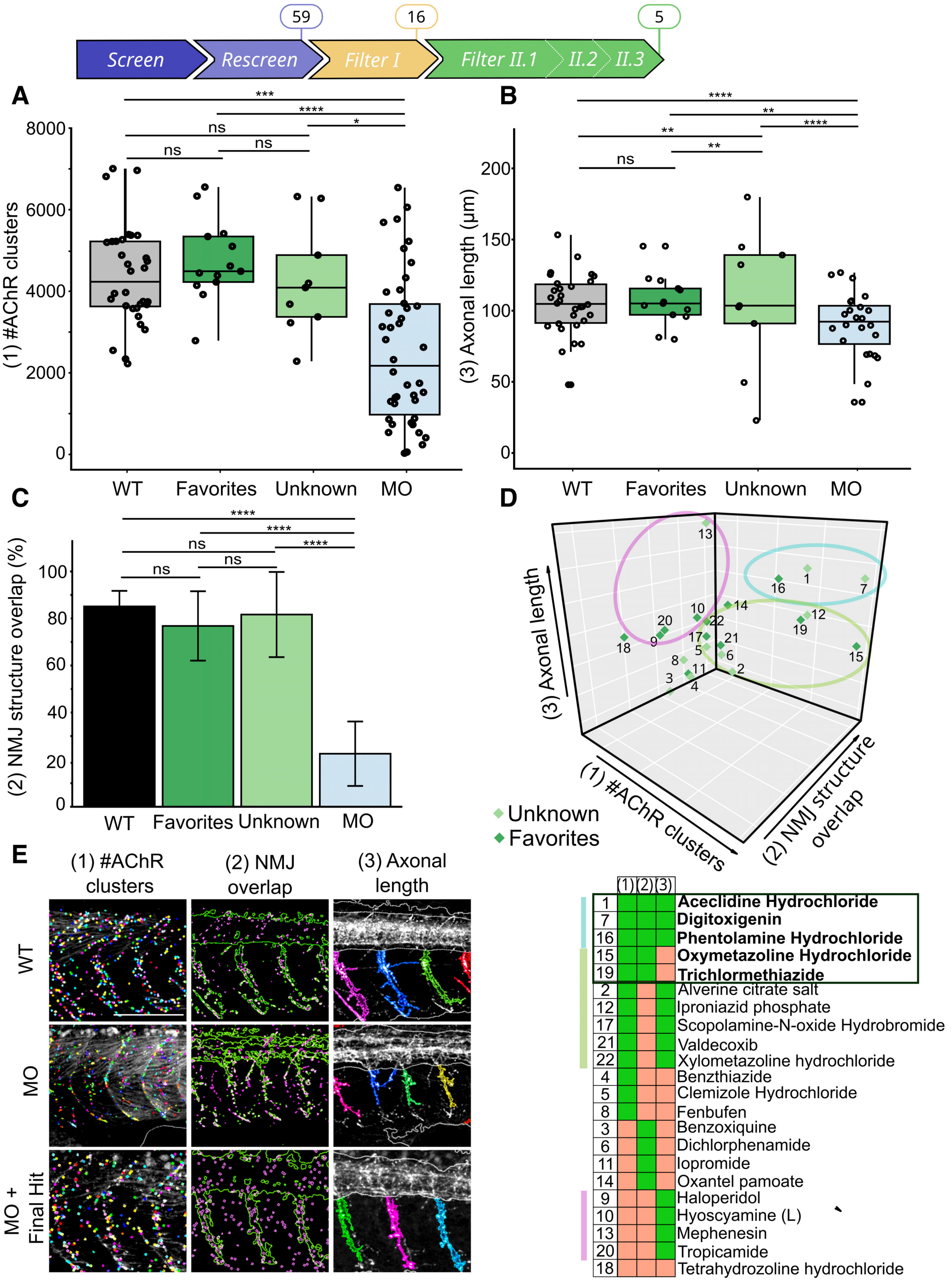

Figure 7. Filter II: The automated imaging-analysis identifies five Hits regulating NMJ development in the *gan* zebrafish.

A–C Boxplots showing individual and mean values of the number of postsynaptic AChR clusters (A) and axonal length (B) and bar plot showing NMJ structure overlap (C) for four groups: noninjected WT (black), MO-injected embryos (blue), MO-injected embryos treated with Favorites Hits (dark green) or Unknown Hits (light green). The central bands of the boxplots represent the median, the boxes of the boxplots represent the interquartile range (between the first and third quartile), and the whiskers represent the minimum and maximum values.

D 3-D scatter plot representing the z-scoring analysis for the three parameters (x (1) = #AChR clusters, y (2) = NMJ structure overlap, z (3) = Axonal length). The 3-D representation and the associated Hit-map identify subgroups of compounds with different penetrance of recovery of cellular deficits in the *gan* zebrafish, among which three Hits restore all parameters and two additional restore two parameters including NMJ common to all Hits.

E Representative images of the three parameters examined for noninjected WT embryos (WT), *gan* MO-injected embryos (MO), and *gan* MO-injected embryos treated with Aceclidine Hydrochloride, which restore all three parameters. Data information: Scale bar represents a length of 200 µm.

Data information: Each dot represents individual values for WT and MO, and mean values of quadruplicate treated larvae with single Hits (Favorites, Unknown) (A–C). In the absence of normality of distribution of the data, a nonparametric Kruskal–Wallis test is applied; medians with range are represented; $n = 34$ (WT), $n = 44$ (MO), $n = 13$ (Favorites), $n = 9$ (Unknown); $^{*}P \leq 0.05$; $^{**}P \leq 0.01$, $^{***}P \leq 0.001$, and $^{****}P \leq 0.0001$.

 © 2023 The Authors

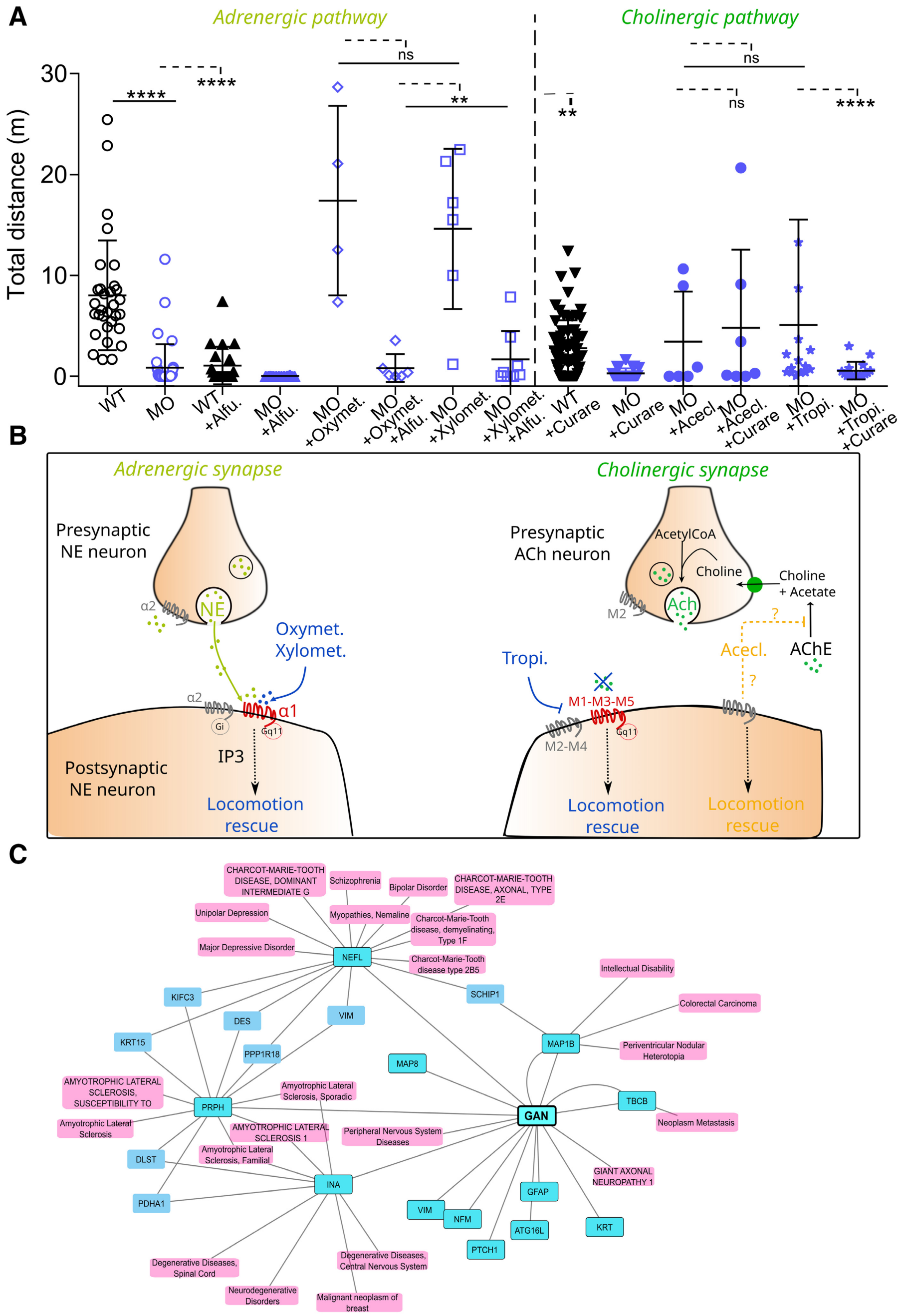

Figure 8. Specificity of the Hits for on-target effects at the NMJ.

A Box plots representing the effect of adrenergic (left, Oxymet., Oxymetazoline Hydrochloride; Xylomet., Xylometazoline Hydrochloride) and cholinergic (right, Acecl., Aceclidine Hydrochloride; Tropi., Tropicamide) Hits, in the presence of pathway-specific blockers (Alfu., Alfuzosin, Curare) added as pretreatment. Each dot represents individual values for non-injected WT embryos, MO-injected larvae and MO-injected larvae treated with the Hits ± blockers. In the absence of normality of distribution of the data, a nonparametric Kruskal–Wallis with Dunn's multiple comparison test is applied; means with ± standard deviation are represented; $n = 31$ (WT), $n = 37$ (MO), $n = 21$ (WT + Alfu), $n = 16$ (MO + Alfu), $n = 4$ (MO + Oxymet.), $n = 6$ (MO + Oxymet. + Alfu.), $n = 6$ (MO + Xylomet.), $n = 8$ (MO + Xylomet. + Alfu.), $n = 69$ (WT + Curare), $n = 23$ (MO + Curare), $n = 6$ (MO + Acecl.), $n = 7$ (MO + Acecl. + Curare), $n = 15$ (MO + Tropi.), $n = 16$ (MO + Tropi. + Curare). $*P \leq 0.05$; $**P \leq 0.01$, $***P \leq 0.001$ and $****P \leq 0.0001$ with WT values.

B Proposed mechanisms of action of the adrenergic (left) and cholinergic (right) Hits on associated synapses. G-coupled receptors are represented at the pre- or postsynaptic membrane according to their localization. Hits whose locomotion rescue action is expected to be dependent on direct action on receptors are shown in blue. Conversely, Hit whose action is assumed to be independent of its role as an agonist is shown in orange. NE, Norepinephrin; ACh, Acetylcholine; AChE, Acetylcholine-esterase.

C Interaction's network between GAN and human diseases. Gigaxonin's substrates (dark blue) and associated diseases (light pink) were manually added to the initial list. The gene-disease-associated interactions with (KRT15, VIM, DES) are not displayed because too numerous, but they seem principally associated with cancer. The network of diseases closest to GAN mainly includes NMD. We distinguish several groups of neuromuscular pathologies, including numerous CMT forms, motor neuron pathology (ALS) and myopathies.

© 2023 The Authors

discovery (Patton *et al*, 2021). This vertebrate species is particularly suitable for screening strategies aimed at re-profiling compounds used in clinical trials and therefore to identify novel modifiers of disease-related phenotypes. A major benefit of drug repurposing lies in the rapid transitioning from preclinical models to patients, as the toxicity and tolerability profiles of the relevant compounds have been documented (FDA-approved drugs; Pushpakom *et al*, 2019). Thus, the use of zebrafish has been particularly effective in the repositioning of molecules in different biomedical fields (see MacRae & Peterson, 2015 for review).

Combining zebrafish model and pharmacological screening constitutes a fast and unbiased way to identify therapeutic molecules, regardless of the known mechanisms underlying disease. This represents a considerable advantage for the GAN pathology, as the contribution of the known molecular targets of gigaxonin (Intermediate Filaments (Mahammad *et al*, 2013; Bomont, 2016) and the autophagic ATG16L1 protein (Scrivo *et al*, 2019)) to neuronal impairment and neurodegeneration is yet to be determined (Lescouzères & Bomont, 2020). More recently, our group revealed that gigaxonin controls the turnover of the Ptch receptor (Arribat *et al*, 2019), to positively modulate Sonic Hedgehog pathway activity, one of the key developmental machinery sustaining neuron and muscle fates in vertebrates (Jessell, 2000; Te Kronnie & Reggiani, 2002). In *gan* zebrafish, Shh activity is reduced and leads to impaired motoneuron stability and an abolishment of neuromuscular junction formation with a typical denervation profile, which are combined to abolish locomotion in gigaxonin-depleted animals. While this indicates that a strategy-enhancing Shh activity may have beneficial effects in GAN, the goal of the present study was to develop an unbiased approach toward drug discovery, independently of the Shh-mediated effects in our *gan* zebrafish model.

Thus, the present study aimed at identifying in our *gan* zebrafish model small molecules able to counteract the loss of motility underlying the GAN disease and compensate for the associated cellular deficits. With a repurposing approach, we screened the Prestwick library containing 95% of FDA-approved drugs and created an innovative pipeline of multilevel methodologies to quantify, score, and identify the mode of action of drug candidates. Thus, we combined *in vivo* behavioral-based readout tests in two independent *gan* zebrafish models (Screens & Rescreen), computational approach using System Biology (Filter I), and a novel automated imaging-based analysis (Filter II 1–3).

Our multilevel pipeline for drug discovery in zebrafish presents the asset of introducing different scales of analysis, from behavior to automated and high-content imaging-based methodology, which can be applied to other neuromuscular diseases (Lescouzères *et al*, 2022). Indeed, most screenings in zebrafish have been performed using a unique functional parameter, and very few studies have integrated computational methods of large-scale data in the discovery of new medical targets (Walker *et al*, 2012; Rennekamp & Peterson, 2015). So far, screenings in zebrafish primarily covered either (i) high-content analysis to evidence rescue of locomotor parameters (Kokel *et al*, 2010; Rihel *et al*, 2010; Laggner *et al*, 2012), or (ii) image-based assays of a small panel of selected Hits (Lemmens *et al*, 2007; Kawahara *et al*, 2011; Van Hoecke *et al*, 2012; Oprişoreanu *et al*, 2021). Overall, our stepwise approach allowed the identification of five compounds that restore *gan* deficits from behavior to cellular scales, and from which we could predict their biological target(s) and mode of action.

Noteworthy, all Hits targets are meaningful with the impaired motor neuron organization, abolished neuromuscular junctions, and locomotion loss described in *gan* zebrafish. Indeed, the five selected Hits, efficient in rescuing loss of locomotion and denervation profile, belong to three pharmacological families (Cholinergic, Adrenergic, and Carbonic Anhydrase agent) known to play a key role in NMJ (Wright *et al*, 2009; Bertone *et al*, 2017; Du *et al*, 2017; Li *et al*, 2018; Bukharaeva *et al*, 2021). Among the most efficient drug candidates, we encounter one Cholinergic agent (Aceclidine Hydrochloride), two Adrenergic agents (Phentolamine hydrochloride, Oxymetazoline Hydrochloride), one CA agent (Trichlormethiazide), and one unknown Hit (Digitoxigenin). Importantly, all these Hits restore the hallmark of neuromuscular synapses by (i) increasing the area of co-localization of axonal nerve terminals and AChR clusters in *gan* morphants, (ii) improving AChR clustering, and for some (iii) rescuing axonal pathfinding.

Interestingly, synapse-stabilizing compounds have also been identified in zebrafish models for other NMDs, such as spinal muscular atrophy (SMA; Oprişoreanu *et al*, 2021) and amyotrophic lateral sclerosis (ALS; Lemmens *et al*, 2007; Van Hoecke *et al*, 2012; McGown *et al*, 2016; Patten *et al*, 2017; Bose *et al*, 2019). While most of the studies monitor axonal length and NMJ overlap, only very few laboratories specifically investigate the clustering of AChR at the pre- and postsynapse. Among those, an elegant work performed on the chodl zebrafish model of SMA offered an extensive characterization of the recovery of the NMJ upon drug administration, with a large spectrum of parameters including the area and labeling-intensity of pre- and postsynaptic AChRs (Oprişoreanu *et al*, 2021). We conducted similar analysis to reveal two additional alterations of the NMJ in the *gan* zebrafish model (Fig EV4): a decrease in the mean labeling-intensity of presynaptic receptors and an increased area of the postsynaptic receptors. Interestingly, other zebrafish models also revealed enlargement of AChR microclusters: col19a1 mutation (Panzer *et al*, 2005), constitutive activation of the MuSK kinase (Mazhar & Herbst, 2012), and deletion (Wang *et al*, 2008) and knockdown (Sun *et al*, 2015) of choline acetyltransferase (ChAT). Surprisingly, the favorite Hits were not able to rescue neither the labeling-intensity of presynapse, nor the area of the postsynaptic AChRs. This indicates that while the drugs are not able to reverse all the alterations of the synaptic compartments, their beneficial action on axonal innervation and AChR clustering numbers is sufficient to restore motility in treated *gan* animals.

The mechanisms of improved NMJ neurotransmission by cholinergic and adrenergic agents may be numerous. The fact that Xylometazoline hydrochloride and Oxymetazoline Hydrochloride lose their effect on locomotion following pretreatment with adrenoblockers indicates a possible direct role for α1 agonists (Haenisch *et al*, 2010). Conversely, the effect of cholinergic Hits seems to be more ambivalent. Indeed, Tropicamide, a muscarinic ACh receptor (mAChR) antagonist, loses its positive effect on locomotion after pretreatment with competitive cholinergic antagonism, suggesting a possible direct role for cholinergic receptor. On the contrary, given that pretreatment with cholinergic blocker treatment led to only partial rescue of the motility by Aceclidine Hydrochloride, it is conceivable that additional pathways, independent of postsynaptic cholinergic receptors, are also involved (Fig 8B).

 © 2023 The Authors

Although there is no agreement about whether all mAChR subtypes are present in the NMJ (Garcia *et al*, 2005; Wright *et al*, 2009), their role in ACh release during development and adulthood is well known. Here, we identified a specific role of mAChR antagonists (Alverine, Scopolamine, Fig 7D) in rescuing postsynaptic AChR clustering and neuromuscular synaptic contact. Scopolamine is mostly known as antidepressant agent and can influence synaptic activity and function by increasing synaptogenesis in prefrontal cortex (Voleti *et al*, 2013). Interestingly, several studies conducted on mammals and amphibians have showed that muscarinic cholinoreceptors, particularly the M1 and M2 subtypes also modulate synaptic transmission in neuromuscular junctions (Re, 1999; Tsentsevitsky *et al*, 2017).

Our results show that Aceclidine Hydrochloride is one of the most effective Hit, restoring the three neuromuscular parameters studied, independently of its cholinergic agonist activity (Fig 8A and B). Aceclidine Hydrochloride is a well-known parasympathomimetic that stimulates muscarinic receptor but also potentiates cholinergic activity through its action as cholinesterase inhibitor (AChEI). Thus, it is largely conceivable that the effect of this Hit on the restoration of locomotion is due, directly to the potentiation of ACh effect due to a decrease in the rate of AChE-catalyzed hydrolysis of the neurotransmitter and its increased availability at NMJ enhancing neuromuscular transmission (Fig 8B). Interestingly, some AChEI are currently used in therapy to ameliorate neuromuscular disorders caused by deficiency in synaptic AChRs, such congenital myasthenic syndromes (CMS; Finsterer, 2019) or Myasthenia Gravis (MG; Maggi & Mantegazza, 2011). The AChEI pyridostigmine is also under investigation (Phase II clinical trial Identifier: NCT02941328) for spinal muscular atrophy (Stam *et al*, 2018). This cholinergic agent has already shown positive effect on ACh availability at NMJ, enhancing neuromuscular transmission and improving muscular strength in X-linked myotubular myopathy zebrafish models and patients (Robb *et al*, 2011), with clinical features common to congenital myasthenic syndromes.

Remarkably, a subgroup of Cholinergic agents has been identified to specifically restore axonal pathfinding. They exert a sympathomimetic effect that contributes to axonal pathfinding and navigation by modulating the rate and direction of axonal growth (Zheng *et al*, 1994).

On the contrary, the mechanisms underlying improved neurotransmission by adrenergic agents may be numerous and may include postsynaptic expansion of end plates, growth of presynaptic nerve terminals or restoration of normal levels of neurotransmitter release through their agonists and antagonist activity. Alpha-adrenoreceptor agonist are mostly known as neuroprotective treatment in glaucoma (Arthur & Cantor, 2011), but also known to play an important role in central neurotransmission, synaptic plasticity, and cognition (Perez, 2020). Our results (Fig 8B) led us to focus especially on the α1 receptor, abundant in the smooth muscles. There are diverse neuromuscular therapeutic applications for these adrenergic agents. Oxymetazoline Hydrochloride was shown to ameliorate pathologies in the LGMD2I zebrafish model of limb-girdle muscular dystrophy (Serafini *et al*, 2018). Unexpectedly, adrenergic agents showed relevant regulation of ACh release from the presynaptic nerve terminals and postsynaptic sensitivity (Bukharaeva *et al*, 2021) and could be combined with cholinergic agents for better action. Thus, the adrenergic agonist salbutamol, in association with AChEI, enhances neuromuscular junction synaptic structure in genetic myasthenia mouse models and improves muscle strength and fatiguability in patients (Vanhaesebrouck *et al*, 2019). It may help the emergence of sympathomimetics for treating neurodegenerative diseases accompanied by synaptic defects such as GAN, through an increased efficiency in the transmission of synaptic excitation.

Interestingly, we also identified Carbonic Anhydrase Inhibitors (CAIs; with Trichloromethiazide in the top five Hits). There are part of classic pharmacological agents clinically used for the management of glaucoma and retinal degeneration (Masini *et al*, 2013), but novel applications were recently reported in diabetes, cancer, epilepsy, AD, and cardiovascular disease (Supuran, 2008). A phase II clinical trial (Identifier: NCT02466074) is ongoing to evaluate how the CAI Acetazolamide affects the way in which newly formed Multiple sclerosis lesions evolve and whether tissue repair is improved. Above all, CAIs are known to regulate acetylcholine receptor endocytosis and have been recently identified as a potential therapeutic approach for myasthenia gravis (Du *et al*, 2017). In the literature, some studies demonstrated that CAIs had also effective AChE inhibition properties (Aslan *et al*, 2019) and could be used in the same way as AChEI cited above.

Thus, the three different pharmacological families of the identified Hits share a common action in promoting NMJ maintenance at the synaptic compartment. Unveiling a specific role of the NMJ in the pathogenesis of GAN in zebrafish, we suggest to carefully examine its architecture in patients. Moreover, the development of novel rodent models exhibiting severe motor deficits will be crucial to confirm NMJ alterations in disease and may offer the opportunity to perform preclinical studies to assess for the therapeutic benefit of our selected Hits in GAN rodent models, at pre- and postsymptomatic stages. Regarding the translatability of the Hits in human, our results show that the drugs also show efficacy when applied at symptomatic stages during development (Fig EV6 and Movie EV2). To further confirm the clinical value of our Hits, it would be important to characterize the motor phenotype of the *gan* KO zebrafish model during adulthood and evaluate the potency of the selected Hits in restoring locomotion upon delivery at this later symptomatic stage. Several methods of administration have been evaluated in zebrafish and include inhalation, injection, or oral administration (Pugach *et al*, 2009; Kinkel *et al*, 2010; Collymore *et al*, 2013).

In conclusion, we developed a novel multilevel pipeline for drug discovery in zebrafish that can be applied to other neuromuscular diseases. This strategy led to the identification of small molecules whose mode of actions are highly relevant for the GAN pathology, by promoting NMJ stability and improving neuroprotection. This study represents the first pharmacological approach for GAN, which will mark the starting point of further preclinical studies that may be translated to human in the near future. Beyond GAN, the identified Hits may offer interesting perspectives for numerous diseases (Fig 8C), including NMD, neurological diseases, and myopathies.

Materials and Methods

Zebrafish husbandry

Zebrafish (*Danio rerio*, Oregon AB) were maintained at 28.5°C on a 14:10 h light: dark and developmental stages were defined by hours (h) or days (d) postfertilization (hpf and dpf).

© 2023 The Authors

Morpholino knockdown

The *gan* antisense Morpholino Oligonucleotides (MOs, Gene Tools; Arribat *et al*, 2019) were designed to target an exon splice donor site (exon 2–3; 5′AGAGTGATCTACAGAAGGAAACAGT) causing splicing defects of the gan mRNA. 1 nl volume of *gan* MO ex2-3 was injected into embryos at one- to two-cell stage at a concentration of 0.25 mM and according to standard protocols.

CRISPR *gan* zebrafish

The $gan^{del/del}$ zebrafish line was generated (Arribat *et al*, 2019) using the genome-editing technology CRISPR (AMAGEN, Gif-sur-Yvette, France). This method generated the $gan^{del/+}$ line, in which the deletion encompasses the entire gan gene: between the middle of exon 1 and downstream the stop codon in exon 11. The resulting Open Reading Frame is restricted in exon1 and contains several premature STOP codons, with only 43 amino acids produced. F3 Transgenic *gan* zebrafish were crossed to obtain fertilized $gan^{del/del}$ eggs for the study.

Genotyping of the *gan* KO line

Genotyping was performed as following: Genomic DNA was extracted from tail pieces collected from anesthetized embryos. Lysis was achieved with 10 mM Tris–HCl pH 8, 2 mM EDTA pH 8, 0.2% Triton 100×, proteinase K, followed by boiling for 10′ at 96°C. Genomic DNA containing deleted sites was PCR-amplified using primers with the following sequences: forward primer: 5′-AATTACAACCCACCAAAG-3′; reverse primer: 5′-GTCGAGGCTTCAGTGTCAT-3′; and separated on 2% agarose gel.

Pilot study

A pilot study was performed to establish the screening conditions as presented in Fig EV1. First, different dechorionation methods were tested: mechanical dechorionation with forceps or enzyme-supported dechorionation with pronase. After identifying pronase as the best solution at this early age, different duration (from 2 to 10 min) and different concentrations (0.1 to 2 mg/ml) were tested. 1 mg/ml pronase treatment for 7 min was identified as the most effective agent for the homogeneous dechorionation at early developmental stage. This treatment significantly reduced interindividual variability and permitted to standardize the response to drug treatments. Next, the optimal number of fish treated with drug in single well (1 to 5 fish/well in 96-well plates) was determined using the z’ factor, and then experimentally tested by comparing the development of the animals (morphology and motility readouts). This identified four fish/well as the best condition ensuring both statistical power and the absence of negative effect on development. Finally, we determined the appropriate concentration of DMSO that can be safely used as the adjuvant of the drugs. Concentrations ranging 0.1 to 2% of DMSO were tested and allowed to select 1% as a concentration not disturbing the general morphology and motility of the embryos.

Drugs/small molecules library

The Prestwick Chemical Library (Illkirch, France), containing 1,280 molecules among which 95% of FDA-approved drug was chosen to provide a high degree of chemical and pharmacological diversity, and to ensure bioavailability and safety in humans for repositioning purpose. The library was provided at 10 mM concentration in DMSO, in 16 96-well plates, each containing 80 compounds. The original plates were diluted at 1:10 to create an intermediate stock solution in daughter-plates at 1 mM with 100% dimethyl sulfoxide (DMSO).

Drug treatment

At 6 hpf, embryos were treated with 1 mg/ml pronase for 7 min under agitation and subsequently washed three times with E3 medium. Residual chorion debris were removed, and the dechorionated embryos were returned to the incubator for 30 min at 28.5°C to rest before proceeding to the Screen. Eight hours postfertilization, dechorionated embryos were manually arrayed into 96-well plates (Nunclon, Nunc™, ThermoFisher; four embryos per well) containing 100 µl E3 medium (5 mM NaCl; 0.17 mM KCl; 0.33 mM $CaCl_2$; 0.33 mM $MgSO_4$; 10^{-5}% Methylene Blue), using a 200-µl wide-bore pipette tip. Plates containing stock solutions of drugs (1 mM) were thawed and diluted in H_2O to a 2× concentrated solution of 20 µM 2% DMSO using a liquid handling robot (FreedomEVO200, Tecan). No mix at this step was performed to avoid aspirating embryos, and addition of drugs was performed at 3 mm from the well bottom to avoid any contact with embryos. Therefore, the final concentration of drugs was 10 µM with 1% DMSO in a total volume of 200 µl per well. As internal controls, noninjected WT and MO-injected WT larvae were included in columns 1 and 12 of each assay plate (as indicated in Fig EV1) and treated with H_2O containing 1% DMSO. Plates were incubated in an automated incubator (Cytomat 6001 C450, ThermoFisher) at 28.5°C and on a 14:10 h light: dark cycle until 48 hpf, when drugs were mechanically washed five times (FreedomEVO200, Tecan) with 200 µl of E3 medium. These washes were performed by a suction of 150 µl and redistribution of 150 µl medium with two slow mixes (10 µl/s) and a soaking for 5 min without agitation. Embryos from each well were then analyzed at different time points according to the following methods. The same protocol was applied for fish treated at a symptomatic stage (48 hpf, Fig EV6). In this case, the drug baths (at increasing concentration from 5 to 30 µM) were renewed every day until 5 dpf.

Touch-response assay

The touch-response test was performed at 48 hpf. Dechorionated embryos were subjected to a slight mechanical stimulation, and the induced motility was recorded by a video camera. Representative tracking from movies was obtained with the ImageJ software.

Miniaturized locomotion assay

Five-day-old treated embryos were redistributed in four 96-well assay plates (Nunclon, Nunc™, ThermoFisher) with a single fish per well, following the same plate design than in the “treatment-plate.” The 64 “reading-plates” were used to carry out the locomotion assay. The spontaneous motility of individual larvae was recorded for 1 h in the dark using the Zebrabox system (Viewpoint Life Sciences, Lyon, France), and the total distance was quantified using the tracking mode of ZebraLab software.

 © 2023 The Authors

Data analysis (Screen & Rescreen)

The observer was blinded to the treatment. First, locomotion data were filtered out from recorded videos to exclude empty wells and wells containing embryos for which movement corresponds to a false-positive or false-negative output. Second, a quantitative scoring of movement was applied to the data using R programming language (R Core Team, 2019) and robustness and reproducibility of the test was evidenced by the calculation of the Z′-factor (Zhang *et al*, 1999) using the following equation:

$$Z' - factor = 1 - \frac{3(\sigma_p + \sigma_n)}{|\mu_p - \mu_n|}$$

where μ_p and σ_p are the median and median absolute deviation values of the positive controls (or alternately, the wild-type samples) and μ_n and σ_n are those of the negative controls (or alternately, the *gan* samples).

Screen

All 1,280 drugs from Prestwick Chemical Library were screened in quadruplicate, and each assay plate and the total distance were normalized with internal controls (noninjected WT larvae). As a median-based normalization was applied (Malo *et al*, 2006), plate's effects were corrected by the median value across wells that are annotated as noninjected WT controls. The effect on each individual treated fish was measured by calculating the Z-score as compared to noninjected WT controls using the following equation:

$$z - score = \frac{x'ki - median(c'i)}{mad(c'i)}$$

where x' is the normalized values, c' is the normalized values of WT controls, k-th the well, i-th the plate.

Rescreen

The 103 Hits coming out of the first screening were retested on $gan^{del/del}$ KO line. The normalization method also known as "Normalized Percentage Activation" (NPA) was used. This method was chosen here because it is the most appropriate if many sample values are giving an effect (enrichment of positive drugs in comparison to screen I) and high variability. Here, the measure relies on calculating a well result by dividing the difference between sample measurements and the average of negative controls through the difference between positive and negative controls.

$$x' = \frac{\mu_{ni} - Xki}{\mu_{pi} - \mu_{ni}} * 100$$

where X is the raw value, μ_n is the median of the negative control, μ_p is the median of the positive control, k-th well and i-th plate.

Filter I: System biology analysis

To recover the protein targets associated with each drug, we used DrugBank (version 5.1.8), a freely available web resource containing detailed drug-target interactions for FDA-approved drugs ([DATABASE] DrugBank; Wishart *et al*, 2018). We identified 93 targets for the 59 common Hits. We next seek the functional interactions between these 93 targets in the STRING v.11 (Szklarczyk *et al*, 2019) database, selecting interactions from all sources and keeping only high confidence interaction (score > 0.7). This created a network of 89 nodes and 427 edges. We integrated and visualized the drug–target and target–target interaction with Cytoscape v.3.6 (Shannon, 2003). Target node sizes were scaled according to the number of drugs targeting the node.

Filter II: Imaging analysis

Immunohistochemistry of zebrafish embryos

Zebrafish were treated with 75 μM 1-Phenyl-2-thiourea (PTU, Sigma) from 24 hpf to prevent pigmentation. After drug-wash, they were anesthetized at appropriate developmental stages with 0.0168% tricaine (MS-222, Sigma-E10521-50G), fixed in 4% PFA for 4 h at RT, and permeabilized in 1× PBS-1%TritonX-100 for 2 h on an orbital shaker. Subsequently, embryos were incubated in blocking buffer (1% DMSO, 1% normal donkey serum, 1% BSA, and 0.7% TritonX-100, PBS) for 1 h at RT and incubated in primary antibodies overnight at 4°C. Primary antibodies are from the following sources: mouse IgG2a antisynaptotagmin (1:100, Znp-1, DSHB, RRID:AB 2315626), anti-α-bungarotoxin (1:50, B35451, Invitrogen), and antisynaptic vesicle glycoprotein 2 (1:200, SV2, DSHB, AB 2315387). Following 0.1% TritonX-100: PBS washes, embryos were incubated in secondary antibodies (Alexa 488, 1:500, Jackson Labs 200-542-211) overnight at 4°C and subsequently washed in PBS prior to imaging.

High-content image acquisition

Note that the automated imaging protocol used here has been described in detail Lescouzères *et al* (2022). Single fluorescent zebrafish larvae were manually cut at the anterior part of the yolk and placed in single wells of a black F-bottom 96-well assay plates (μClear, Greiner Bio-one) in a lateral position to ensure better recognition of the body shape of each larva. High-content imaging was performed using the imaging technology of Opera Phenix™ "High Content Screening System confocal" (PerkinElmer). Images were first acquired with a Pres-Scan mode in wide-field mode at 5× magnification to locate the zebrafish in well with on-the-fly image analysis. To automatically find zebrafish larvae positions in wells, a prescan at 5× magnification was used to cover the entire well surface (nine fields per well), only on the red channel (561 nm), with a signal distributed throughout the embryo. Using the on-the-fly image analysis, a global image of the whole well was created, and the "Find Image Region" module of the Harmony® High-content analysis software (v4.9, PerkinElmer) was used to set the appropriate intensity threshold to detect the fish. The "Determine Well Layout" module was then used to define a rescan magnification, of 20× with an overlap of 6% between fields, covering the entire object. Then, a second automated acquisition (Re-Scan) of this specific position was made in confocal mode at 20× magnification (z-stack 90 μm), allowing imaging of the whole larva and a complete image analysis. Well areas containing whole zebrafish larvae were automatically imaged in confocal mode at 20× magnification on green (znp-1) and red (α-bungarotoxin) channels. A z-

© 2023 The Authors

stack of 90 μm (5 μm interval) was applied, creating an on-the-fly image analysis to obtain a global image with Maximum Intensity Projections (MIP). From AChR-channel global image, the "Find Image Region" and "Select Region" modules allowed to detect fish body and subtract 7 pixels around to restrict the analysis to the region of interest (Fig 6A). "Find Image Region" module was then applied on the green channel global image to detect and measure size and area of the Axonal region, that is, spine and axons (Fig 6B). The "Find spots" module (method D) was applied with a specific intensity threshold to locate AChR clusters on the red channel global image (Fig 6B and D(1)). For AChR quantification, AChR number, area, and intensity were measured using the "Calculate Position Properties – Cross population" module. The coefficient of co-localization was obtained using the same module in a fraction of the myotome to quantify NMJ (Fig 6B and D(2)). Then, analysis and quantification of output parameters (#AChR clusters, NMJ structure overlap and Axonal length) were obtained with Harmony® High-content analysis software (v4.9, PerkinElmer). On the same images batch, confocal rescan at 20× magnification, another automated analysis was conducted. After creating a global image with Maximum Intensity Projections for the two channels, a smoothing with a median filter of 20 px was applied on the green channel global image (Fig 6C) to bring out the densest region of the global image. Using the "Find Surrounding Region" module, the spinal cord area was subtracted from the fish body area. A new region of interest was then created using the "Modify Population" module, corresponding to axonal region without the spinal cord. This specific region was used to measure axonal length, width, area, and the ratio of body length to mean axonal length (Fig 6D(3)).

Fluorescence intensity analysis

The ImageJ contour mask tool was used to create a ROI around three somites for each image analyzed. After subtracting the background, the ROI mask was applied and then the measure tool was used to obtain the mean gray value (Fig EV4A and B).

Pharmacological treatment with curare and alfuzosin

Zebrafish embryos were manually arrayed into 96-well-plates as described above. We conducted preliminary dose response for Curare (1, 3, 5, 10 mM) and Alfuzosin (1, 3, 5, 10 mM). Concentrations of 3 mM Alfuzosin and 5 mM Curare resulted in strong effects and limited toxicity and were selected for further experiments. Zebrafish embryos were pretreated with adrenergic blocker (Alfuzosin, Prestwick Chemical Library) or cholinergic blocker (Curare, Sigma). After 2 h of treatment, adrenergic or cholinergic Hits identified in our screen (Oxymetazoline Hydrochloride, Xylometazoline Hydrochloride, Aceclidine Hydrochloride, and Tropicamide) were respectively added to the fish water at a 10 μM concentration. Plates were incubated in an automated incubator until 48 hpf, when drugs were automatically washed five times with E3 medium. Embryos from each well were then analyzed at using Zebrabox at 5 dpf as described in the "Miniaturized locomotion assay" section.

Statistics

The statistical significance of the differences between experimental groups was determined by the R programming language (R Core Team, 2019). The assessment of the normality of the distribution of the data was determined with the Shapiro–Wilk test, to apply either a parametric or nonparametric test. We used the Kruskal–Wallis test (Hollander *et al*, 2014), a nonparametric test to compare the difference between experimental groups for analysis of projection lengths, area AChR cluster, NMJ junction and overlap. When differences in variance between groups were significant according to the Kruskal–Wallis test, we performed *post hoc* tests (i.e., pairwise comparisons using Mann–Whitney test) to identify the groups for which the differences were significant. For pairwise comparisons, significance values were adjusted with Holm correction for multiple testsv (Holm, 1979). The differences between experimental groups are deemed significant for $*P \leq 0.05$; $**P \leq 0.01$, $***P \leq 0.001$, and $****P \leq 0.0001$.

Study approval

Experiments on zebrafish were conducted prior 5-day postfertilization, which corresponds to the nonautonomous stage of the animals and does not require specific authorization accordingly to the Directive 2010/63/EU. We obtained the approval of the ethics committee and the French ministry (reference N°036) for the creation of the *gan* zebrafish line.

The paper explained

Problem

Giant axonal neuropathy (GAN) is an early onset and severe neurodegenerative disorder. Fatale in young adults, the disease first touches the sensory and motor system, leading to the loss of sensibility and ambulation in teens. To date, there is no cure for GAN, and the mouse models exhibit a too mild phenotype to represent a valuable tool to test the efficacy of any therapeutic approach. We generated the first robust models for GAN in zebrafish, which reproduce the loss of motility in patients (Arribat *et al*, 2019), hence representing the first hope for a phenotypic-driven therapy.

Results

In our study, we conducted a pharmacological screening on our *gan* zebrafish models with a repurposing strategy of small therapeutic molecules. Thus, we developed a stepwise approach integrating behavioral, computational, and a novel high-content imaging-based cellular analysis. This multilevel screening pipeline allowed us to identify five Hits acting at the neuromuscular junction, restoring motility through a beneficial action on the postsynaptic compartment.

Impact

This study identifies the first therapeutic molecules for GAN, able to restore the motility and the related cellular defects in the robust *gan* zebrafish models. Importantly, the Hits are beneficial when applied both at pre- and postsymptomatic stages, hence offering promising translational development for patients. Furthermore, our novel high-content imaging methodology represents a useful automated procedure for mechanistic studies and drug screening for other neuromuscular conditions. Altogether, we developed a multilevel pipeline and identified therapeutic candidates for GAN that can be both impactful to NMD, neurological diseases, and myopathies.

© 2023 The Authors

Data availability

This study includes no data deposited in external repositories.

Expanded View for this article is available online.

Acknowledgements

We thank the Aquatic Model Platform of the CRBM institute (Univ. Montpellier, CNRS, Montpellier) and B. Delaval for the coordination of housing and support, P. Richard, M. Plays for zebrafish husbandry. We acknowledge the SFR Biosciences (UMS3444/CNRS, ENS, University of Lyon) zebrafish facility, specifically L. Bernard and R. Renard for their technical assistance. We are also grateful to the IGF Institute (Montpellier) and especially C. Joplin and A. Faucherre for the provision of the Zebrabox. We acknowledge the imaging facility MRI, member of the national infrastructure France-BioImaging supported by the French National Research Agency (ANR-10-INBS-04, "Investments for the Future"). We thank J. L. Bessereau and M. Jospin for discussion on pharmacology and for providing curare. This work was funded by l'Institut National de la Santé et de la Recherche Médicale (INSERM) to PB, and the Fondation pour la Recherche Médicale to LL (FRM, fellowship PLP20170939065). PB is supported by grants from the ATIP-Avenir program (INSERM), The French Muscular Dystrophy Association (AFM-Téléthon) (grant 17654), and the Fondation Maladies Rares (FMR). We are grateful to H. Barzilai-Tutsch (INMG-MeLiS) for critical reading and comments on the manuscript.

Author contributions

Léa Lescouzères: Data curation; software; formal analysis; investigation; methodology; writing – original draft; writing – review and editing. **Cédric Hassen-Khodja:** Resources; software; formal analysis. **Anaïs Baudot:** Software. **Benoît Bordignon:** Resources; data curation; software; formal analysis; methodology. **Pascale Bomont:** Conceptualization; resources; data curation; supervision; funding acquisition; validation; investigation; writing – original draft; project administration; writing – review and editing.

Disclosure and competing interests statement

The authors declare that they have no conflict of interest.

References

[DATABASE] DrugBank https://go.drugbank.com/

Arribat Y, Mysiak KS, Lescouzères L, Boizot A, Ruiz M, Rossel M, Bomont P (2019) Sonic hedgehog repression underlies gigaxonin mutation–induced motor deficits in giant axonal neuropathy. *J Clin Invest* 129: 5312–5326

Arthur S, Cantor LB (2011) Update on the role of alpha-agonists in glaucoma management. *Exp Eye Res* 93: 271–283

Asbury AK, Gale MK, Cox SC, Baringer JR, Berg BO (1972) Giant axonal neuropathy—a unique case with segmental neurofilamentous masses. *Acta Neuropathol* 20: 237–247

Aslan HE, Demir Y, Özaslan MS, Türkan F, Beydemir Ş, Küfrevioglu Öl (2019) The behavior of some chalcones on acetylcholinesterase and carbonic anhydrase activity. *Drug Chem Toxicol* 42: 634–640

Berg BO, Rosenberg SH, Asbury AK (1972) Giant axonal neuropathy. *Pediatrics* 49: 894–899

Bertone NI, Groisman AI, Mazzone GL, Cano R, Tabares L, Uchitel OD (2017) Carbonic anhydrase inhibitor acetazolamide shifts synaptic vesicle recycling to a fast mode at the mouse neuromuscular junction. *Synapse* 71: e22009

Best JD, Alderton WK (2008) Zebrafish: an in vivo model for the study of neurological diseases. *Neuropsychiatr Dis Treat* 4: 567–576

Boizot A, Talmat-Amar Y, Morrogh D, Kuntz NL, Halbert C, Chabrol B, Houlden H, Stojkovic T, Schulman BA, Rautenstrauss B *et al* (2014) The instability of the BTB-KELCH protein Gigaxonin causes Giant axonal neuropathy and constitutes a new penetrant and specific diagnostic test. *Acta Neuropathol Commun* 2: 47

Bomont P (2016) Degradation of the intermediate filament family by Gigaxonin. *Methods Enzymol* 569: 215–231

Bomont (2019) GAN (gigaxonin) E3 ligase and ATG16L1: master and commander of autophagosome production. *Autophagy* 15: 1650–1652

Bomont P, Koenig M (2003) Intermediate filament aggregation in fibroblasts of giant axonal neuropathy patients is aggravated in non dividing cells and by microtubule destabilization. *Hum Mol Genet* 12: 813–822

Bomont P, Cavalier L, Blondeau F, Hamida CB, Belal S, Tazir M, Demir E, Topaloglu H, Korinthenberg R, Tüysüz B *et al* (2000) The gene encoding gigaxonin, a new member of the cytoskeletal BTB/kelch repeat family, is mutated in giant axonal neuropathy. *Nat Genet* 26: 370–374

Bose P, Tremblay E, Maios C, Narasimhan V, Armstrong GAB, Liao M, Parker JA, Robitaille R, Wen XY, Barden C *et al* (2019) The novel small molecule TRVA242 stabilizes neuromuscular junction defects in multiple animal models of amyotrophic lateral sclerosis. *Neurotherapeutics* 16: 1149–1166

Bukharaeva E, Khuzakhmetova V, Dmitrieva S, Tsentsevitsky A (2021) Adrenoceptors modulate cholinergic synaptic transmission at the neuromuscular junction. *Int J Mol Sci* 22: 4611

Cavalier L, BenHamida C, Amouri R, Belal S, Bomont P, Lagarde N, Gressin L, Callen D, Demir E, Topaloglu H *et al* (2000) Giant axonal neuropathy locus refinement to a < 590 kb critical interval. *Eur J Hum Genet* 8: 527–534

Cleveland DW, Yamanaka K, Bomont P (2009) Gigaxonin controls vimentin organization through a tubulin chaperone-independent pathway. *Hum Mol Genet* 18: 1384–1394

Collymore C, Rasmussen S, Tolwani RJ (2013) Gavaging adult zebrafish. *J Vis Exp* 50691

Dequen F, Bomont P, Gowing G, Cleveland DW, Julien J-P (2008) Modest loss of peripheral axons, muscle atrophy and formation of brain inclusions in mice with targeted deletion of gigaxonin exon 1. *J Neurochem* 107: 253–264

Du A, Huang S, Zhao X, Feng K, Zhang S, Huang J, Miao X, Baggi F, Ostrom RS, Zhang Y *et al* (2017) Suppression of CHRN endocytosis by carbonic anhydrase CAR3 in the pathogenesis of myasthenia gravis. *Autophagy* 13: 1981–1994

Ehlert FJ, Griffin MT, Glidden PF (1996) The interaction of the enantiomers of aceclidine with subtypes of the muscarinic receptor. *J Pharmacol Exp Ther* 279: 1335–1344

El-Brolosy MA, Kontarakis Z, Rossi A, Kuenne C, Günther S, Fukuda N, Kikhi K, Boezio GLM, Takacs CM, Lai S-L *et al* (2019) Genetic compensation triggered by mutant mRNA degradation. *Nature* 568: 193–197

Finsterer J (2019) Congenital myasthenic syndromes. *Orphanet J Rare Dis* 14: 57

Ganay T, Boizot A, Burrer R, Chauvin J, Bomont P (2011) Sensory-motor deficits and neurofilament disorganization in gigaxonin-null mice. *Mol Neurodegener* 6: 25

Garcia N, Santafé MM, Salon I, Lanuza MA, Tomàs J (2005) Expression of muscarinic acetylcholine receptors (M1-, M2-, M3- and M4-type) in the neuromuscular junction of the newborn and adult rat. *Histol Histopathol* 20: 733–743

© 2023 The Authors

Giacomotto J, Ségalat L (2010) High-throughput screening and small animal models, where are we?: high-throughput screening and small animal models. *Br J Pharmacol* 160: 204–216

Haenisch B, Walstab J, Herberhold S, Bootz F, Tschaikin M, Ramseger R, Bönisch H (2010) Alpha-adrenoceptor agonistic activity of oxymetazoline and xylometazoline. *Fundam Clin Pharmacol* 24: 729–739

Hollander M, Wolfe DA, Chicken E (2014) *Nonparametric statistical methods*, 3rd edn. Hoboken, NJ: John Wiley & Sons, Inc

Holm S (1979) A simple sequentially rejective multiple test procedure. *Scand J Stat* 6: 65–70

Jessell TM (2000) Neuronal specification in the spinal cord: inductive signals and transcriptional codes. *Nat Rev Genet* 1: 20–29

Juneja M, Burns J, Saporta MA, Timmerman V (2019) Challenges in modelling the Charcot-Marie-tooth neuropathies for therapy development. *J Neurol Neurosurg Psychiatry* 90: 58–67

Kawahara G, Karpf JA, Myers JA, Alexander MS, Guyon JR, Kunkel LM (2011) Drug screening in a zebrafish model of Duchenne muscular dystrophy. *Proc Natl Acad Sci USA* 108: 5331–5336

Kinkel MD, Eames SC, Philipson LH, Prince VE (2010) Intraperitoneal injection into adult zebrafish. *J Vis Exp* 2126

Kokel D, Bryan J, Laggner C, White R, Cheung CYJ, Mateus R, Healey D, Kim S, Werdich AA, Haggarty SJ *et al* (2010) Rapid behavior-based identification of neuroactive small molecules in the zebrafish. *Nat Chem Biol* 6: 231–237

Kuhlenbäumer G, Timmerman V, Bomont P (2020) Giant axonal neuropathy. In *GeneReviews®*, Adam MP, Ardinger HH, Pagon RA, Wallace SE, Bean LJ, Stephens K, Amemiya A (eds). Seattle, WA: University of Washington

Laggner C, Kokel D, Setola V, Tolia A, Lin H, Irwin JJ, Keiser MJ, Cheung CYJ, Minor DL, Roth BL *et al* (2012) Chemical informatics and target identification in a zebrafish phenotypic screen. *Nat Chem Biol* 8: 144–146

Laurá M, Pipis M, Rossor AM, Reilly MM (2019) Charcot-Marie-tooth disease and related disorders: an evolving landscape. *Curr Opin Neurol* 32: 641–650

Lemmens R, Van Hoecke A, Hersmus N, Geelen V, D'Hollander I, Thijs V, Van Den Bosch L, Carmeliet P, Robberecht W (2007) Overexpression of mutant superoxide dismutase 1 causes a motor axonopathy in the zebrafish. *Hum Mol Genet* 16: 2359–2365

Lescouzères L, Bomont P (2020) E3 ubiquitin ligases in neurological diseases: focus on Gigaxonin and autophagy. *Front Physiol* 11: 1022

Lescouzères L, Bordignon B, Bomont P (2022) Development of a high-throughput tailored imaging method in zebrafish to understand and treat neuromuscular diseases. *Front Mol Neurosci* 15: 956582

Li L, Xiong W-C, Mei L (2018) Neuromuscular junction formation, aging, and disorders. *Annu Rev Physiol* 80: 159–188

Ma Z, Zhu P, Shi H, Guo L, Zhang Q, Chen Y, Chen S, Zhang Z, Peng J, Chen J (2019) PTC-bearing mRNA elicits a genetic compensation response via Upf3a and COMPASS components. *Nature* 568: 259–263

MacRae CA, Peterson RT (2015) Zebrafish as tools for drug discovery. *Nat Rev Drug Discov* 14: 721–731

Maggi L, Mantegazza R (2011) Treatment of myasthenia gravis: focus on pyridostigmine. *Clin Drug Investig* 31: 691–701

Mahammad S, Murthy SNP, Didonna A, Grin B, Israeli E, Perrot R, Bomont P, Julien J-P, Kuczmarski E, Opal P *et al* (2013) Giant axonal neuropathy–associated gigaxonin mutations impair intermediate filament protein degradation. *J Clin Invest* 123: 1964–1975

Malo N, Hanley JA, Cerquozzi S, Pelletier J, Nadon R (2006) Statistical practice in high-throughput screening data analysis. *Nat Biotechnol* 24: 167–175

Masini E, Carta F, Scozzafava A, Supuran CT (2013) Antiglaucoma carbonic anhydrase inhibitors: a patent review. *Expert Opin Ther Pat* 23: 705–716

Mazhar S, Herbst R (2012) The formation of complex acetylcholine receptor clusters requires MuSK kinase activity and structural information from the MuSK extracellular domain. *Mol Cell Neurosci* 49: 475–486

McGown A, Shaw DPJ, Ramesh T (2016) ZNStress: a high-throughput drug screening protocol for identification of compounds modulating neuronal stress in the transgenic mutant sod1G93R zebrafish model of amyotrophic lateral sclerosis. *Mol Neurodegener* 11: 56

Michel C, Collins C (2020) Pediatric neuromuscular disorders. *Pediatr Clin North Am* 67: 45–57

Morrison BM, Griffin JW (2009) Neuromuscular diseases. In *Cerebrospinal Fluid in Clinical Practice*, pp 121–126. Baltimore: Elsevier

Oprişoreanu A-M, Smith HL, Krix S, Chaytow H, Carragher NO, Gillingwater TH, Becker CG, Becker T (2021) Automated in vivo drug screen in zebrafish identifies synapse-stabilising drugs with relevance to spinal muscular atrophy. *Dis Model Mech* 14: dmm047761

Panzer JA, Gibbs SM, Dosch R, Wagner D, Mullins MC, Granato M, Balice-Gordon RJ (2005) Neuromuscular synaptogenesis in wild-type and mutant zebrafish. *Dev Biol* 285: 340–357

Pappalardo A, Pitto L, Fiorillo C, Alice Donati M, Bruno C, Santorelli FM (2013) Neuromuscular disorders in zebrafish: state of the art and future perspectives. *Neuromolecular Med* 15: 405–419

Patten SA, Aggad D, Martinez J, Tremblay E, Petrillo J, Armstrong GA, La Fontaine A, Maios C, Liao M, Ciura S *et al* (2017) Neuroleptics as therapeutic compounds stabilizing neuromuscular transmission in amyotrophic lateral sclerosis. *JCI Insight* 2: e97152

Patton EE, Zon LI, Langenau DM (2021) Zebrafish disease models in drug discovery: from preclinical modelling to clinical trials. *Nat Rev Drug Discov* 20: 611–628

Perez DM (2020) α1-adrenergic receptors in neurotransmission, synaptic plasticity, and cognition. *Front Pharmacol* 11: 581098

Peterson RT, Link BA, Dowling JE, Schreiber SL (2000) Small molecule developmental screens reveal the logic and timing of vertebrate development. *Proc Natl Acad Sci USA* 97: 12965–12969

Pugach EK, Li P, White R, Zon L (2009) Retro-orbital injection in adult zebrafish. *J Vis Exp* 1645

Pushpakom S, Iorio F, Eyers PA, Escott KJ, Hopper S, Wells A, Doig A, Guilliams T, Latimer J, McNamee C *et al* (2019) Drug repurposing: progress, challenges and recommendations. *Nat Rev Drug Discov* 18: 41–58

R Core Team (2019) *R: a language and environment for statistical computing*. Vienna: R Foundation for Statistical Computing

Ravi B, Antonellis A, Sumner CJ, Lieberman AP (2019) Genetic approaches to the treatment of inherited neuromuscular diseases. *Hum Mol Genet* 28: R55–R64

Re L (1999) Modulation of acetylcholine release by presynaptic muscarinic autoreceptors. *Acta Physiol Pharmacol Ther Latinoam* 49: 215–223

Rennekamp AJ, Peterson RT (2015) 15 years of zebrafish chemical screening. *Curr Opin Chem Biol* 24: 58–70

Rihel J, Prober DA, Arvanites A, Lam K, Zimmerman S, Jang S, Haggarty SJ, Kokel D, Rubin LL, Peterson RT *et al* (2010) Zebrafish behavioral profiling links drugs to biological targets and rest/wake regulation. *Science* 327: 348–351

Robb SA, Sewry CA, Dowling JJ, Feng L, Cullup T, Lillis S, Abbs S, Lees MM, Laporte J, Manzur AY *et al* (2011) Impaired neuromuscular transmission and response to acetylcholinesterase inhibitors in centronuclear myopathies. *Neuromuscul Disord* 21: 379–386

Schenone M, Dančík V, Wagner BK, Clemons PA (2013) Target identification and mechanism of action in chemical biology and drug discovery. *Nat Chem Biol* 9: 232–240

 © 2023 The Authors

Scrivo A, Codogno P, Bomont P (2019) Gigaxonin E3 ligase governs ATG16L1 turnover to control autophagosome production. *Nat Commun* 10: 780

Serafini PR, Feyder MJ, Hightower RM, Garcia-Perez D, Vieira NM, Lek A, Gibbs DE, Moukha-Chafiq O, Augelli-Szafran CE, Kawahara G *et al* (2018) A limb-girdle muscular dystrophy 2I model of muscular dystrophy identifies corrective drug compounds for dystroglycanopathies. *JCI Insight* 3: e120493

Shannon P (2003) Cytoscape: a software environment for integrated models of biomolecular interaction networks. *Genome Res* 13: 2498–2504

Stam M, Wadman RI, Wijngaarde CA, Bartels B, Asselman F-L, Otto LAM, Goedee HS, Habets LE, de Groot JF, Schoenmakers MAGC *et al* (2018) Protocol for a phase II, monocentre, double-blind, placebo-controlled, cross-over trial to assess efficacy of pyridostigmine in patients with spinal muscular atrophy types 2-4 (SPACE trial). *BMJ Open* 8: e019932

Sun J, Pan CQ, Chew TW, Liang F, Burmeister M, Low BC (2015) BNIP-H recruits the cholinergic machinery to neurite terminals to promote acetylcholine signaling and neuritogenesis. *Dev Cell* 34: 555–568

Supuran CT (2008) Carbonic anhydrases: novel therapeutic applications for inhibitors and activators. *Nat Rev Drug Discov* 7: 168–181

Szklarczyk D, Gable AL, Lyon D, Junge A, Wyder S, Huerta-Cepas J, Simonovic M, Doncheva NT, Morris JH, Bork P *et al* (2019) STRING v11: protein–protein association networks with increased coverage, supporting functional discovery in genome-wide experimental datasets. *Nucleic Acids Res* 47: D607–D613

Te Kronnie G, Reggiani C (2002) Skeletal muscle fibre type specification during embryonic development. *J Muscle Res Cell Motil* 23: 65–69

Tsentsevitsky AN, Kovyazina IV, Nurullin LF, Nikolsky EE (2017) Muscarinic cholinoreceptors (M1-, M2-, M3- and M4-type) modulate the acetylcholine secretion in the frog neuromuscular junction. *Neurosci Lett* 649: 62–69

Van Hoecke A, Schoonaert L, Lemmens R, Timmers M, Staats KA, Laird AS, Peeters E, Philips T, Goris A, Dubois B *et al* (2012) EPHA4 is a disease modifier of amyotrophic lateral sclerosis in animal models and in humans. *Nat Med* 18: 1418–1422

Vanhaesebrouck AE, Webster R, Maxwell S, Rodriguez Cruz PM, Cossins J, Wickens J, Liu W-W, Cetin H, Cheung J, Ramjattan H *et al* (2019) β2-adrenergic receptor agonists ameliorate the adverse effect of long-term pyridostigmine on neuromuscular junction structure. *Brain* 142: 3713–3727

Voleti B, Navarria A, Liu R-J, Banasr M, Li N, Terwilliger R, Sanacora G, Eid T, Aghajanian G, Duman RS (2013) Scopolamine rapidly increases mammalian target of rapamycin complex 1 signaling, synaptogenesis, and antidepressant behavioral responses. *Biol Psychiatry* 74: 742–749

Walker SL, Ariga J, Mathias JR, Coothankandaswamy V, Xie X, Distel M, Köster RW, Parsons MJ, Bhalla KN, Saxena MT *et al* (2012) Automated reporter quantification In vivo: high-throughput screening method for reporter-based assays in zebrafish. *PLoS ONE* 7: e29916

Wang M, Wen H, Brehm P (2008) Function of neuromuscular synapses in the zebrafish choline-acetyltransferase mutant bajan. *J Neurophysiol* 100: 1995–2004

Wishart DS, Feunang YD, Guo AC, Lo EJ, Marcu A, Grant JR, Sajed T, Johnson D, Li C, Sayeeda Z *et al* (2018) DrugBank 5.0: a major update to the DrugBank database for 2018. *Nucleic Acids Res* 46: D1074–D1082

Wright MC, Potluri S, Wang X, Dentcheva E, Gautam D, Tessler A, Wess J, Rich MM, Son Y-J (2009) Distinct muscarinic acetylcholine receptor subtypes contribute to stability and growth, but not compensatory plasticity, of neuromuscular synapses. *J Neurosci* 29: 14942–14955

Zhang J-H, Chung TDY, Oldenburg KR (1999) A simple statistical parameter for use in evaluation and validation of high throughput screening assays. *J Biomol Screen* 4: 67–73

Zheng JQ, Felder M, Connor JA, Poo M (1994) Turning of nerve growth cones induced by neurotransmitters. *Nature* 368: 140–144

License: This is an open access article under the terms of the Creative Commons Attribution License, which permits use, distribution and reproduction in any medium, provided the original work is properly cited.

Expanded View Figures

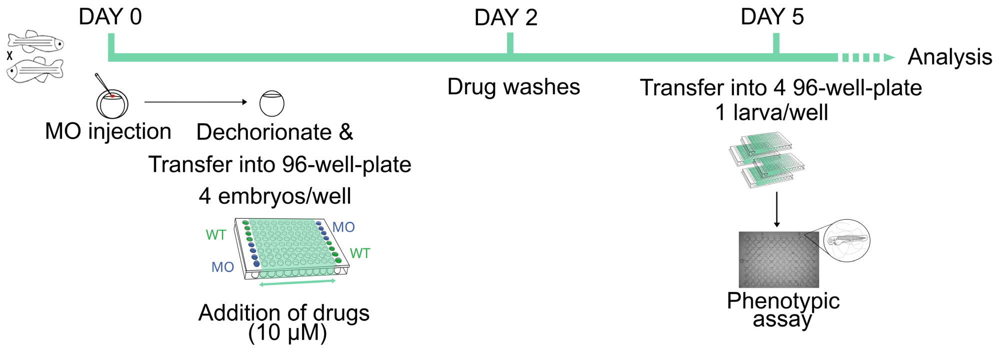

Figure EV1. Schematic representation of the timeline and workflow of the drug screening in the *gan* zebrafish model.

At 0 hpf, WT eggs are injected with *gan* morpholino (MO injection). At 6 hpf, noninjected WT and MO-injected eggs are dechorionated and distributed in 96-well plates (4 eggs/well) and plates are incubated at 8 hpf in fish water with/without drugs at 10 μM concentration. At 2 dpf, drugs are washed. At 5 dpf, larvae are transferred in quadruplicate plates (1 larva/well) and processed to the motility assay.

Figure EV2. Exhaustive representation of the z-scores obtained in the Screen for the 1,280 compounds.

For each drug, individual z-scores of quadruplicate fish are plotted and assessed for the total distance traveled at 5 dpf by *gan* MO-injected larvae for 1 h following treatment with compound. Dotted lines show the mean z-score for untreated *gan* MO-injected larvae (blue) and non-injected WT larvae (red). Toxic drugs are represented in gray (without associated z-score), non-Hits in red, B Hits in orange and A Hits in green.

© 2023 The Authors

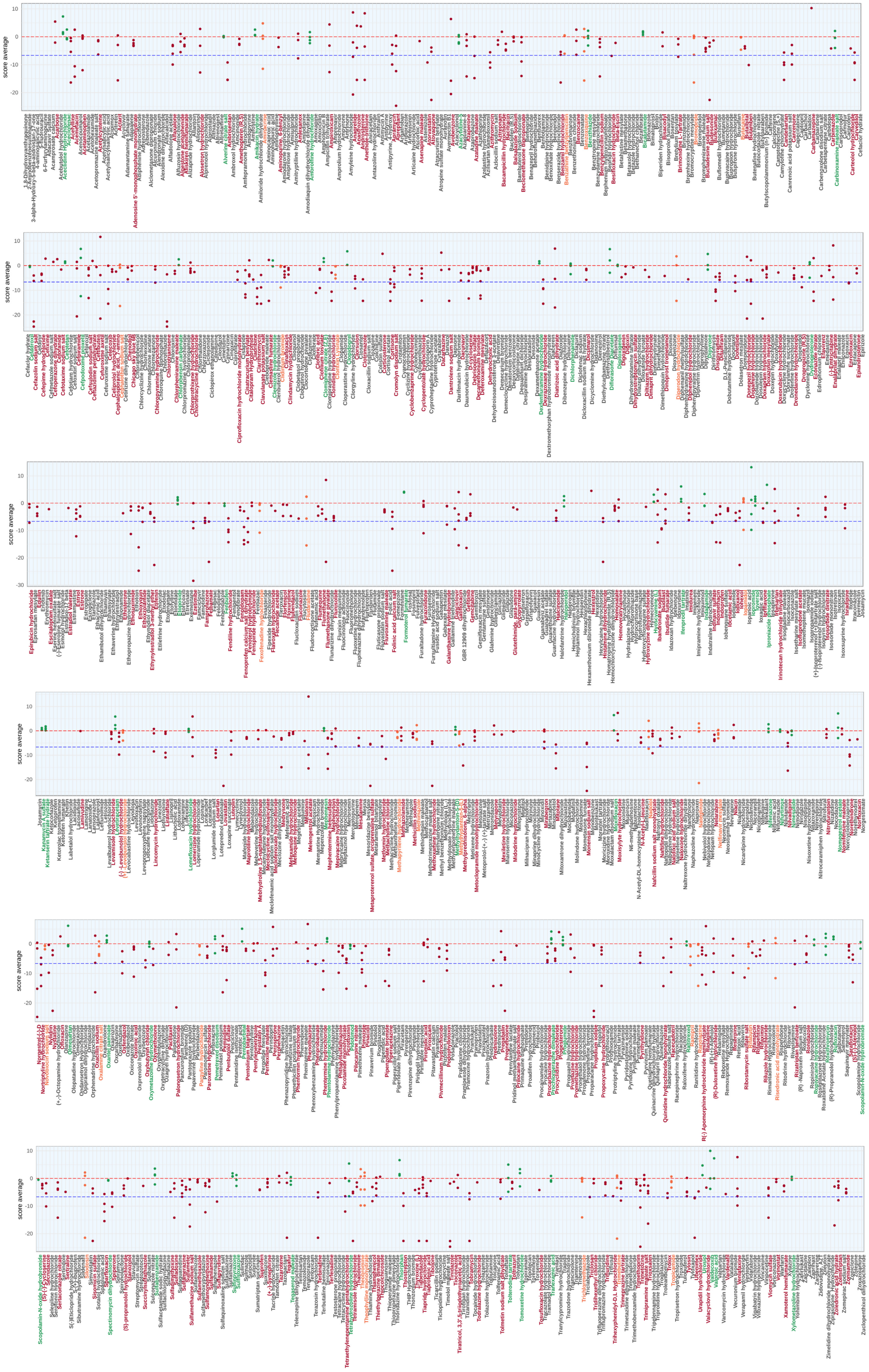

Figure EV2.

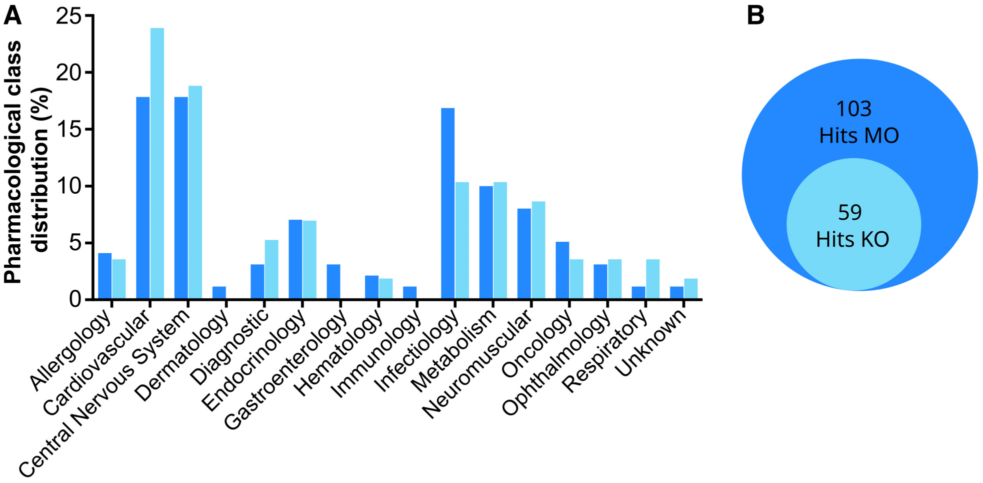

Figure EV3. Pharmacological class of the Hits.

A, B Bar plot (A) showing the distribution of the therapeutic classes (%) of the 59 Hits common to the *gan* morphants and the *gan* KO line (B), according to the Prestwick Chemical Library annotations.

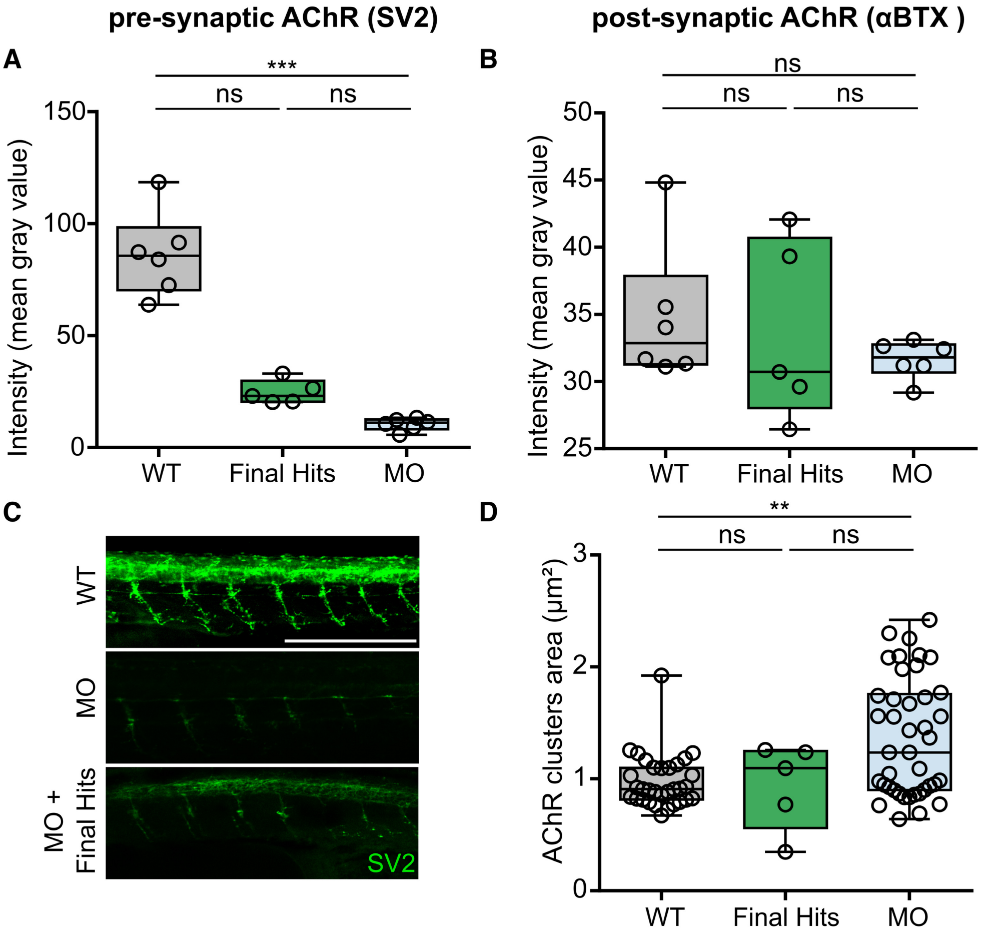

Figure EV4.

 © 2023 The Authors

Figure EV4. Additional AChR-related phenotypes in the *gan* zebrafish, which are not rescued by the five Final Hits.

A–D Boxplots showing individual and mean values of the labeling intensity of presynaptic (stained with Synaptic Vesicle glycoprotein 2 (SV2)) (A) and postsynaptic (stained with α-bungarotoxin (αBTX)) (B) AChR clusters and area of post-synaptic AChR (D) for three groups: noninjected WT (black), MO-injected embryos (blue), MO-injected embryos treated with the five Final Hits (dark green), as identified in Fig 7. (C) Representative images of SV2 intensity labeling for noninjected WT embryos (WT), *gan* MO-injected embryos (MO) and *gan* MO-injected embryos treated with Phentolamine Hydrochloride. Analysis performed at 48 hpf. Scale bar represents a length of 500 μm. Each dot represents individual values for WT (*n* = 6 (A, B), *n* = 34 (D)) and MO (*n* = 6 (A, B), *n* = 43 (D)), and mean values of quadruplicate treated larvae (*n* = 5 (A, B, D)) with single Hits (A, B, D). The central bands of the boxplots represent the median, the boxes of the boxplots represent the interquartile range (between the first and third quartile), and the whiskers represent the minimum and maximum values.

In the absence of normality of distribution of the data, a nonparametric Kruskal–Wallis test is applied; medians with range are represented; $^{**}P \leq 0.01$, $^{***}P \leq 0.001$.

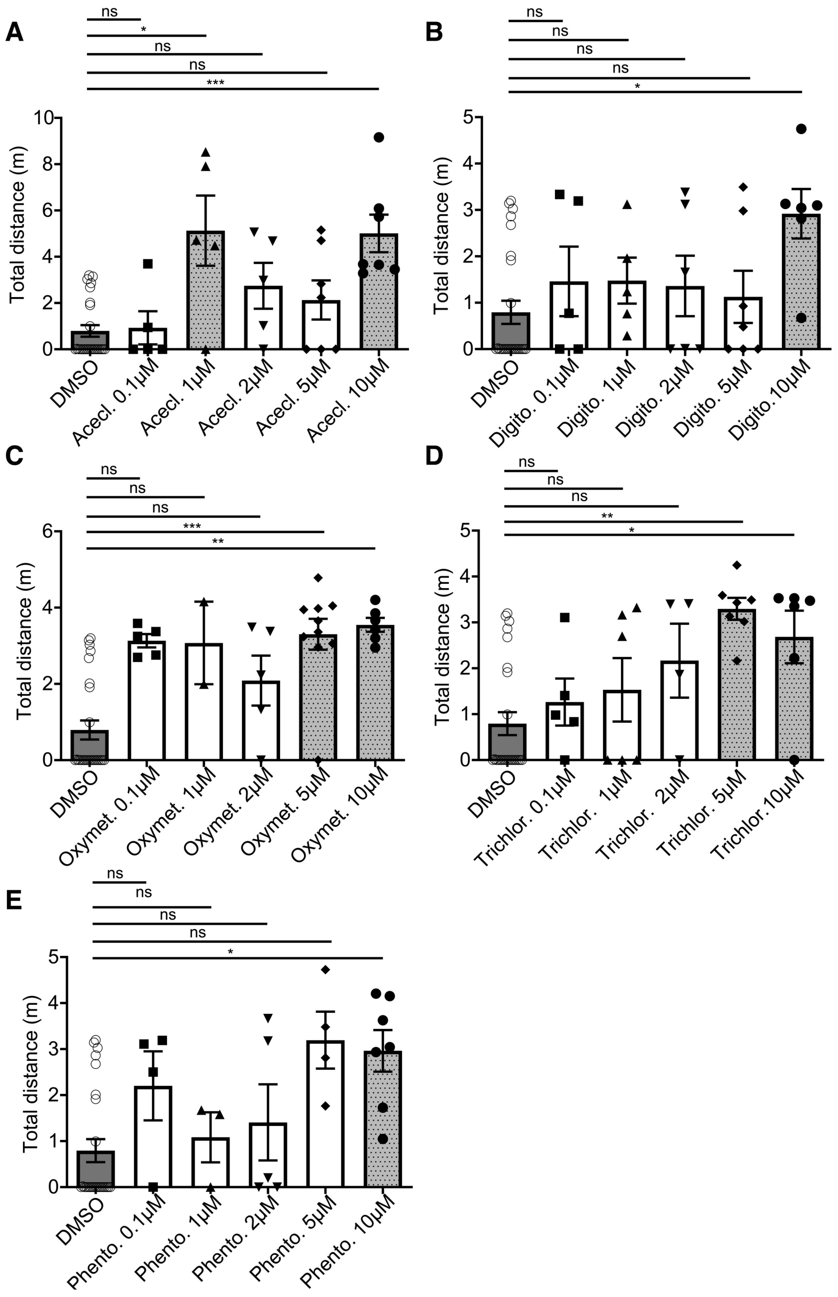

Figure EV5. Dose–response effect of the five favorite Hits on the restoration of motility.

A–E Treatment with Aceclidine Hydrochloride (Acecl.) (A), Digitoxigenin (Digito.) (B), Oxymetazoline Hydrochloride (Oxymet.) (C), Trichlormethiazide (Trichlor.) (D) and Phentolamine Hydrochloride (Phento.) (E) was performed from 0.1 to 50 μM.

Data information: Data are not provided for the higher doses (30 and 50 μM) due to toxicity. Results show the distance traveled at 5 dpf, during 1 h by *gan* MO-injected larvae, treated with either DMSO or Hits from 0.1 to 10 μM.
Each dot represents individual values, (A) *n* = 25 (MO + DMSO), *n* = 5 (MO + Acecl. 0.1 μM), *n* = 5 (MO + Acecl. 1 μM), *n* = 5 (MO + Acecl. 2 μM), *n* = 7 (MO + Acecl. 5 μM), *n* = 7 (MO + Acecl. 10 μM); (B) *n* = 25 (MO + DMSO), *n* = 5 (MO + Digito. 0.1 μM), *n* = 5 (MO + Digito. 1 μM), *n* = 6 (MO + Digito. 2 μM), *n* = 7 (MO + Digito. 5 μM), *n* = 5 (MO + Digito. 10 μM); (C) *n* = 25 (MO + DMSO), *n* = 5 (MO + Oxymet. 0.1 μM), *n* = 2 (MO + Oxymet. 1 μM), *n* = 5 (MO + Oxymet. 2 μM), *n* = 10 (MO + Oxymet. 5 μM), *n* = 6 (MO + Oxymet. 10 μM); (D) *n* = 25 (MO + DMSO), *n* = 5 (MO + Trichlor. 0.1 μM), *n* = 6 (MO + Trichlor. 1 μM), *n* = 4 (MO + Trichlor. 2 μM), *n* = 6 (MO + Trichlor. 5 μM), *n* = 6 (MO + Trichlor. 10 μM); (E) *n* = 25 (MO + DMSO), *n* = 4 (MO + Phento. 0.1 μM), *n* = 3 (MO + Phento. 1 μM), *n* = 5 (MO + Phento. 2 μM), *n* = 4 (MO + Phento. 5 μM), *n* = 7 (MO + Phento. 10 μM). In the absence of normality of distribution of the data, a nonparametric Kruskal–Wallis test is applied; means ± SEM are represented. $^{*}P \leq 0.05$; $^{**}P \leq 0.01$, $^{***}P \leq 0.001$ and $^{****}P \leq 0.0001$.

© 2023 The Authors

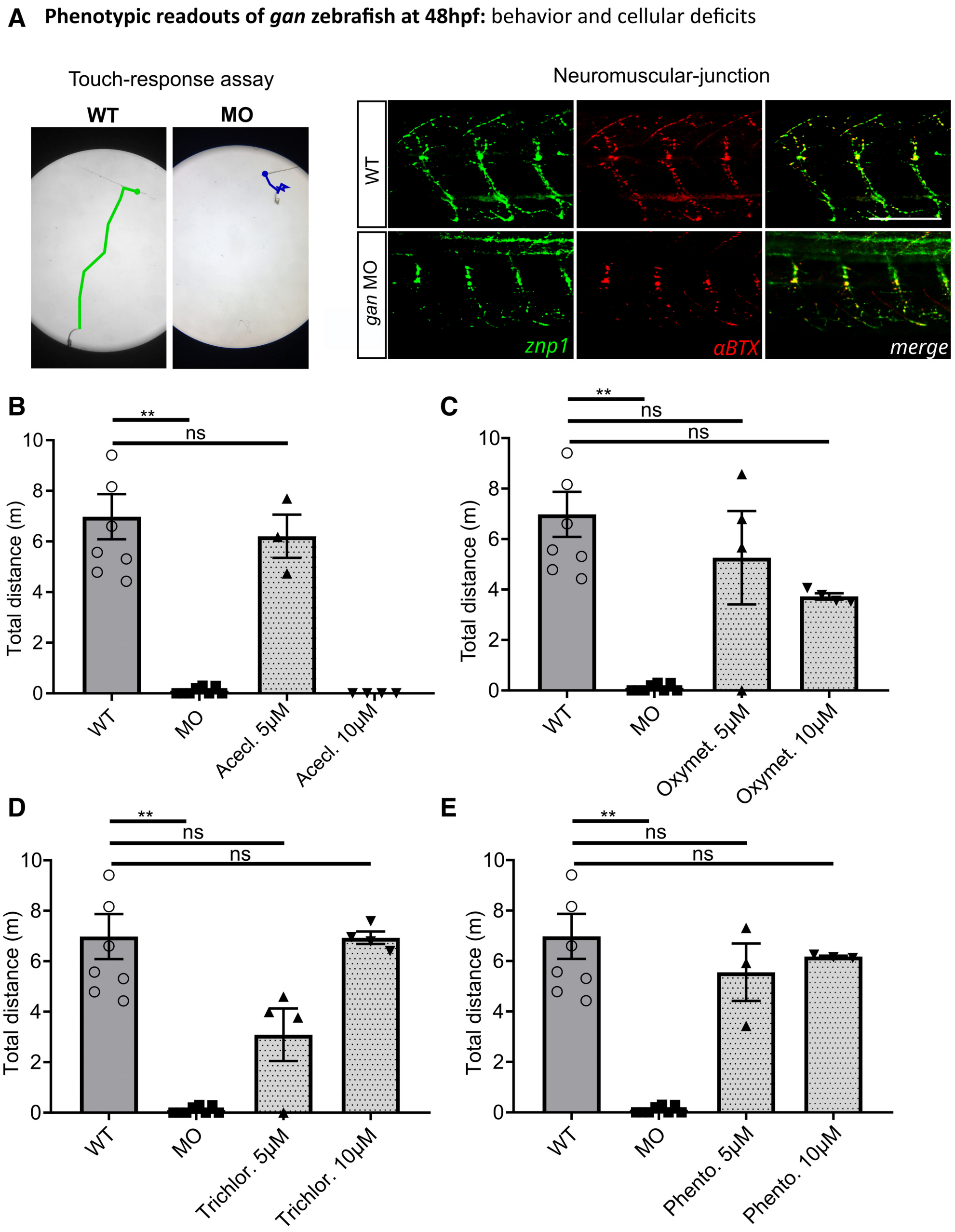

Figure EV6. Beneficial effect of the Hits upon treatment at symptomatic stage.

A Phenotype of *gan* MO-injected embryos at 48 hpf, with deficits in touch-responsiveness and impairment of axons and neuromuscular junctions (znp1: green; αBTX: α-bungarotoxin: red). Scale bar represents a length of 100 μm.

B–E Restoration of motility of 5-day-old *gan* larvae, when treated from 48 hpf with Aceclidine Hydrochloride (Acecl.) (B), Oxymetazoline Hydrochloride (Oxymet.) (C), Trichlormethiazide (Trichlor.) (D) and Phentolamine Hydrochloride (Phento.) (E). Hits were applied from 5 to 30 μM concentrations, with daily bath changes; data are not provided for the higher doses (20 and 30 μM) due to toxicity. Results show the distance traveled during 1 h by *gan* MO-injected larvae, treated with either DMSO or Hits at 5 and 10 μM. In the absence of normality of distribution of the data, a nonparametric Kruskal–Wallis test is applied; means ± SEM are represented. $^{**}P \leq 0.01$.

 © 2023 The Authors

Appendix:

A multi-level screening pipeline in zebrafish identifies therapeutic drugs for GAN

Running Title: Therapeutic drugs for GAN

Léa Lescouzères[1], Cédric Hassen-Khodja[2], Anaïs Baudot[3], Benoît Bordignon[2] and Pascale Bomont[1*].

[1] ERC team, NeuroMyoGene Insitute-PGNM, Inserm U1315, CNRS UMR5261, University of Lyon 1, Lyon, France.[2]Montpellier Ressources Imagerie, BioCampus, CNRS, INSERM, Université de Montpellier, Montpellier, France.[3]Aix Marseille Univ, INSERM, MMG, Marseille Medical Genetics, CNRS, Marseille, France.

*Correspondence and lead contact: pascale.bomont@inserm.fr

Appendix Table S1: Exhaustive list of data generated in the Rescreen in the gan KO line. Single raw values of the total distance travelled by quadruplicate *gan* KO fish over 1h period are presented in the columns named 1-4. The Normal Percentage Activation (NPA, or "Norm 1-4") are shown for each fish. The average NPA values, used to construct the dot plots in **Fig 4** are shown in the last column of the table.

**103 Hits**	**1**	**2**	**3**	**4**	**Norm 1**	**Norm 2**	**Norm 3**	**Norm 4**	**Norm Average**
Haloperidol	5,988.6	0.0	4,713.0	17,994.1	78.82	0.00	65.41	145.51	72.44
Tranexamic acid	7,971.0	6,009.7	5,766.1	0.0	104.92	82.60	80.03		89.18
Alverine citrate salt	8,909.0	8,158.5	6,985.9	0.0	117.26	112.13	96.96	0.00	81.59
Aceclofenac	6,749.6	8,476.0	8,705.9	0.0	88.84	116.49	120.83	0.00	81.54
Iproniazide phosphate	7,383.8	7,836.8	5,562.6	0.0	97.19	107.71	77.21		94.03
Mephenesin	7,179.8	8,897.6	7,343.7	0.0	94.50	122.29	101.93	0.00	79.68
Pargyline hydrochloride	6,832.9	5,879.7	5,180.5	10,729.8	89.94	80.81	71.90	86.76	82.35
Nimesulide	4,679.5	6,275.7	7,425.1	0.0	61.59	86.25	103.06		83.63
Fenbufen	5.7	5,683.2	5,939.8	0.0	0.08	78.11	82.44		53.54
Indapamide	6,089.4	6,847.2	6,992.1	31,575.0	80.15	94.11	97.05	255.32	131.66
Xylometazoline hydrochloride	4,759.1	5,576.7	6,123.7	18,891.0	62.64	76.64	84.99	152.76	94.26
Oxymetazoline hydrochloride	4,936.8	5,145.0	4,713.5	0.0	64.98	70.71	65.42	0.00	50.28

Nifenazone	5,210.2	5,173.9	3,144.8	0.0	68.58	71.11	43.65	0.00	45.83
Clemizole hydrochloride	7,153.4	2,566.8	5,192.1	0.0	94.16	35.28	72.06		67.17
Tropicamide	4,690.8	4,956.2	4,441.9	17,607.9	61.74	68.12	61.65	142.38	83.47
Phentolamine hydrochloride	5,728.3	4,945.1	5,693.2	0.0	75.40	67.96	79.02		74.13
Scopolamin-N-oxide hydrobromide	4,678.4	4,483.6	4,631.4	0.0	61.58	61.62	64.28		62.49
Hyoscyamine (L)	3,738.4	5,558.7	5,772.4	0.0	49.21	76.40	80.12		68.57
Chlorphensin carbamate	8,057.4	4,979.3	5,125.8	18,863.2	106.05	68.43	71.14	152.53	99.54
Lomefloxacin hydrochloride	5,441.4	9,445.7	4,919.0	0.0	71.62	129.82	68.27	0.00	67.43
Proglumide	5,802.3	4,339.6	3,879.6	0.0	76.37	59.64	53.85	0.00	47.47
Sulfinpyrazone	4,473.6	6,097.3	3,606.3	0.0	58.88	83.80	50.05	0.00	48.18
Diperodon hydrochloride	5,220.3	5,191.7	4,235.4	0.0	68.71	71.35	58.79		66.28
Ifenprodil tartrate	5,018.4	5,193.4	5,524.6	0.0	66.05	71.38	76.68		71.37
Sulfisoxazole	0.0	4.7	0.0	0.0					
Methapyrilene hydrochloride	4.9	3,988.9	6,700.9	0.0	0.06	54.82	93.01		49.30
Ketanserin tartrate hydrate	5,095.3	6,155.9	5,659.0	0.0	67.07	84.60	78.54		76.74
Kanamycin A sulfate	5,107.1	3,747.5	4,841.0	0.0	67.22	51.50	67.19		61.97

Amikacin hydrate	7,151.7	4,497.3	5,693.3	13,093.9	94.13	61.81	79.02	105.88	85.21
Etoposide	5,498.1	5,948.4	3,390.5	10,305.6	72.37	81.75	47.06	83.33	71.13
Clomiphene citrate (Z, E)	6,448.0	4,801.9	3,922.9	23,903.9	84.87	66.00	54.45	193.29	99.65
Oxantel pamoate	6,588.7	5,627.9	4,658.3	0.0	86.72	77.35	64.66		76.24
Prochlorperazine dimaleate	5,872.8	7,227.2	2.9	19,549.1	77.30	99.33	0.04	158.08	83.69
Cefadroxil	0.0	0.0	0.0	0.0					
Digitoxigenin	7,076.8	6.6	5,913.9	0.0	93.15	0.09	82.08		58.44
Cefotetan	6,053.1	7,374.1	5,981.9	0.0	79.67	101.35	83.03		88.02
Ropivacaine hydrochloride	2.8	0.0	3,293.1	0.0	0.04	0.00	45.71		15.25
Pentobarbital	4,425.3	4,564.0	4,469.8	0.0	58.25	62.73	62.04		61.00
Diflorasone Diacetate	0.0	0.0	0.0	7,835.0	0.00	0.00	0.00	63.36	15.84
Thiorphan	0.3	5.7	0.6	6,763.2	0.00	0.08	0.01	54.69	13.69
Tomoxetine hydrochloride	0.0	2,716.0	5,940.3	4,939.7	0.00	37.33	82.45	39.94	39.93
Aceclidine Hydrochloride	5,138.4	4,056.7	2,126.1	15,457.0	67.63	55.75	29.51	124.99	69.47
Levetiracetam	3,948.4	0.0	4,467.6	0.0	51.97	0.00	62.01	0.00	28.49
Dexfenfluramine hydrochloride	7,161.2	4,991.8	2,707.4	19,453.2	94.26	68.61	37.58	157.30	89.44
Dydrogesterone	6,047.5	7,055.5	6,739.7	0.0	79.60	96.97	93.54		90.04
Tetrahydrozoline hydrochloride	6,268.8	14,717.7	5,823.4	0.0	82.51	202.27	80.83	0.00	91.40

Bromopride	0.6	3,976.4	4,751.8	0.0	0.01	54.65	65.95		40.20
Sulfabenzamide	0.0	209.7	0.0	0.0	0.00	2.88	0.00		0.96
Dipyrone	5,248.8	14,951.4	5,301.0	0.0	69.09	205.49	73.58		116.05
Cephalothin sodium salt	0.7	0.0	7,061.4	0.0	0.01	0.00	98.01	0.00	24.50
Methimazole	5,132.7	6,847.9	3,921.5	0.0	67.56	94.11	54.43	0.00	54.03
Carbinoxamine maleate salt	4,784.3	7,092.6	4,206.4	0.0	62.97	97.48	58.38	0.00	54.71
Spectinomycin dihydrochloride	5,069.2	5,830.2	3,864.3	0.0	66.72	80.13	53.64		66.83
Dichlorphenamide	10,405.2	4,640.6	6,513.8	0.0	136.96	63.78	90.41		97.05
Nadolol	66.0	7.9	0.0	23,337.8	0.87	0.11	0.00	188.72	47.42
Clidinium bromide	1.5	5,523.2	0.0	32,582.0	0.02	75.91	0.00	263.47	84.85
Benzthiazide	4,551.4	6,044.2	5,392.9	17,098.2	59.91	83.07	74.85	138.26	89.02
Trichlormethiazide	0.0	5,032.3	6,145.0	0.0	0.00	69.16	85.29		51.48
Oxalamine citrate salt	0.6	0.0	7,117.1	0.0	0.01	0.00	98.78		32.93
Butacaine	0.0	0.0	0.0	0.0	0.00	0.00			0.00
Novobiocin sodium salt	5,746.5	6,125.9	4,306.5	0.0	75.64	84.19	59.77		73.20
Roxithromycin	5,591.8	13,465.5	4,987.2	23,642.2	73.60	185.06	69.22	191.18	129.77
(+) - Levobunolol hydrochloride	22.1	14.7	0.0	14,121.7	0.29	0.20	0.00	114.19	28.67
Methylhydantoin-5-(L)	0.0	7,390.8	0.0	15,242.0	0.00	101.58		123.25	74.94
Methylhydantoin-5-(D)	6,512.5	0.0	5,506.2	14,442.8	85.72	0.00	76.42	116.79	69.73

Azacytidine-5	6.4	6,321.3	6,191.9	17,450.2	0.08	86.88	85.94	141.11	78.50
Iopamidol	5,801.5	8,598.6	10,439.9	21,002.1	76.36	118.18	144.90	169.83	127.32
Iopromide	3,934.9	7,413.4	5,755.7	20,827.5	51.79	101.89	79.89	168.42	100.50
Theophylline monohydrate	7,558.7	9,458.0	8,188.5	23,097.6	99.49	129.99	113.65	186.77	132.48
Theobromine	10,564.2	9,286.6	9,078.4	0.0	139.05	127.63	126.00		130.90
Bicalutamide	16,813.9	21,359.7	5,838.7	0.0	221.31	293.56	81.04		198.64
Ioversol	4.6	8,888.9	4,140.5	10,491.5	0.06	122.17	57.47	84.84	66.13
Urosiol	0.0	0.0	9.7	0.0	-0.23	-8.36	-0.14		-2.91
Nifurtimox	0.0	0.0	0.0	0.0	-0.23	-8.36	-0.21		-2.93
Benzathine benzylpenicillin	0.0	67.7	0.0	0.0	-0.23	-7.60	-0.21		-2.68
Nomegestrol acetate	0.0	0.0	2,420.8	0.0	-0.23	-8.36	18.31		3.24
Ranolazine	9.8	0.0	0.0	0.0	-0.12	-8.36	-0.21		-2.90
Fexofenadine hydrochloride	0.0	133.7	0.0	0.0	-0.23	-6.86	-0.21		-2.44
Clonixin Lysinate	0.0	0.0	4,466.4	0.0	-0.23	-8.36	33.97		8.46
Ramipril	27.2	0.0	0.0	0.0	0.07	-8.36	-0.21		-2.83
Rifabutin	0.5	8,855.4	0.0	0.0	-0.22	90.55	-0.21		30.04
Valproic acid	3,715.0	5,922.5	5,472.4	0.0	40.48	57.79	41.66		46.65
Fludarabine	31.0	0.0	0.0	4,587.2	0.11	-8.36	-0.21	62.40	13.49

Olmesartan	2,621.8	0.0	0.0	0.0	28.50	-8.36	-0.21		6.64
Moxifloxacin	6,601.4	39.0	0.0	0.0	72.11	-7.92	-0.21		21.32
Formoterol fumarate	0.0	17,960.9	0.9	0.0	-0.23	192.25	-0.21		63.94
Rufloxacin	1,386.1	4,121.4	0.0	0.0	14.96	37.68	-0.21		17.47
Nelfinavir mesylate	0.9	0.0	1.4	0.0	-0.22	-8.36	-0.20		-2.93
Silodosin	2,933.1	10,515.3	5,664.5	3,434.7	31.91	109.09	43.13	46.73	57.72
Trimebutine	0.0	0.8	0.4	4,991.7	-0.23	-8.35	-0.21	67.91	14.78
Amorolfine hydrochloride	0.0	972.0	0.0	0.0	-0.23	2.50	-0.21		0.69
Tegaserod maleate	24.8	48.1	0.0	0.0	0.04	-7.82	-0.21		-2.66
Nateglinide	3,044.4	14,171.1	899.6	9.2	33.13	149.92	6.67	0.12	47.46
Aminacrine	45.1	0.0	10.9	0.0	0.27	-8.36	-0.13		-2.74
Pidotimod	8.8	0.0	0.0	1,912.2	-0.13	-8.36	-0.21	26.01	4.33
Clopidogrel	5,250.9	6,482.3	0.0	7,169.3	57.31	64.04	-0.21	97.53	54.67
Benzoxiquine	111.1	30,199.0	4,506.3	4,714.8	0.99	328.94	34.27	64.14	107.09
Cefpodoxime proxetil	0.0	680.1	0.0	0.0	-0.23	-0.76	-0.21		-0.40
Valdecoxib	0.5	36,524.7	3,280.6	0.0	-0.22	399.60	24.89		141.42
Tolterodine tartrate	18.8	0.0	0.0	0.0	-0.02	-8.36	-0.21		-2.86
Risedronic acid monohydrate	1.1	0.0	1.4	0.0	-0.22	-8.36	-0.20		-2.93
Pemetrexed disodium	6.3	0.0	0.0	0.0	-0.16	-8.36	-0.21		-2.91

Raltitrexed	0.0	0.0	0.0	0.0	-0.23	-8.36	-0.21		-2.93
WT + DMSO 1%	9,015.3	10,204.5	9,325.0	13,898.7	118.66	140.25	129.43	112.39	125.18
WT + DMSO 1%	6,623.5	7,267.8	7,884.4	18,306.6	87.18	99.89	109.43	148.03	111.13
WT + DMSO 1%	8,521.0	10,540.4	7,204.8	20,046.2	112.16	144.86	100.00	162.10	129.78
WT + DMSO 1%	8,385.8	7,101.5	7,209.4	0.0	110.38	97.60	100.06		102.68
WT + DMSO 1%	7,366.0	7,046.1	0.0	12,176.1	96.95	96.84		98.46	97.42
WT + DMSO 1%	7,086.5	7,109.1	7,027.9	10,490.5	93.27	97.70	97.54	84.83	93.34
WT + DMSO 1%	7,487.9	7,424.7	6,507.4	10,989.6	98.56	102.04	90.32	88.87	94.95
WT + DMSO 1%	7,707.0	7,284.4	6,870.1	12,366.6	101.44	100.11	95.35	100.00	99.23
WT + DMSO 1%	9,162.0	3,642.9	8,104.0	7,295.2	100.17	32.33	61.80	99.24	73.39
WT + DMSO 1%	10,119.1	13,234.9	18,478.5	7,406.2	110.65	139.47	141.19	100.76	123.02
WT + DMSO 1%	9,131.8	6,167.9	17,109.2	9,277.2	99.83	60.53	130.72	126.21	104.32
WT + DMSO 1%	4,962.9	16,359.5	9,081.6	0.0	54.15	174.37	69.28	0.00	74.45
KO + DMSO 1%	8,193.4	0.0	0.0	0.0	107.84	0.00	0.00	0.00	26.96
KO + DMSO 1%	16.9	14.7	0.0	0.0	0.22	0.20	0.00	0.00	0.11
KO + DMSO 1%	7,224.2	0.0	0.0	0.0	95.09	0.00	0.00	0.00	23.77
KO + DMSO 1%	9,405.4	0.0	5,859.7	13,422.3	123.80	0.00	81.33	108.54	78.42
KO + DMSO 1%	6,042.9	6,149.4	0.0	0.0	79.54	84.52	0.00	0.00	41.01
KO + DMSO 1%	6,715.6	94.8	0.0	0.0	88.39	1.30	0.00	0.00	22.42
KO + DMSO 1%	5,834.3	0.0	8,588.8	0.0	76.79	0.00	119.21	0.00	49.00

KO + DMSO 1%	8,383.1	0.0	0.0	0.0	110.34	0.00	0.00	0.00	27.59
KO + DMSO 1%	41.6	0.0	0.0	0.0	0.23	-8.36	-0.21	0.00	-2.09
KO + DMSO 1%	0.0	748.3	28.0	299.0	-0.23	0.00	0.00	4.07	0.96
KO + DMSO 1%	529.9	0.0	5,190.8	0.3	5.58	-8.36	39.51	0.00	9.18
KO + DMSO 1%	0.0	0.0	0.0	0.0	-0.23	-8.36	-0.21	0.00	-2.20
KO + DMSO 1%	0.0	1,071.4	0.0	4.6	-0.23	3.61	-0.21	0.06	0.81

Appendix Table S2. Summary of the data generated for each Hit with the Opera Phenix - # AChR clusters. Single associated z-scores for # AChR clusters, normalized with WT values are presented in columns named 1-4 for the gan MO quadruplicate, and the associated median scores and p-value are presented in the following columns.

Chemical name	1	2	3	4	score median	p-value
Alverine citrate salt	1.88				1.88	0.242
Benzthiazide	0.23				0.23	1.000
Dichlorphenamide	-0.77	-0.03	-0.03		-0.03	0.556
Haloperidol	-0.38	-0.05	1.47		-0.05	0.595
Hyoscyamine (L)	-0.08				-0.08	1.000
Oxymetazoline hydrochloride	2.08				2.08	0.242
Phentolamine hydrochloride	2.27	-0.16			1.06	0.392
Scopolamin-N-oxide hydrobromide	0.70	-0.31			0.19	0.802
Tetrahydrozoline hydrochloride	-0.42	-2.17			-1.29	0.200
Trichlormethiazide	-1.11	2.67			0.78	0.856
Tropicamide	-0.97	0.96			-0.01	0.913
Valdecoxib	0.99	-0.21	1.20	1.99	1.09	0.073
Xylometazoline hydrochloride	-0.40	0.68			0.14	0.856
Aceclidine Hydrochloride	1.52	1.81	1.56	-2.55	1.54	0.269
Benzoxiquine	-2.30	-0.96	-3.67	-0.05	-1.63	0.024
Clemizole hydrochloride	0.42	-3.05	1.16		0.42	1.000
Digitoxigenin	2.74	0.92			1.83	0.071
Fenbufen	0.91	-1.35	0.05		0.05	0.846
Iopromide	-2.80	-1.01	1.11		-1.01	0.411
Iproniazide phosphate	1.87				1.87	0.242
Mephenesin	-1.32	1.26			-0.03	1.000
Oxantel pamoate	-0.82	1.44	-2.93		-0.82	0.446

Appendix Table S3. **Summary of the data generated for each Hit with the Opera Phenix - Axonal length.** Single associated z-scores for axonal length, normalized with WT values are presented in columns named 1-4 for the *gan* MO quadruplicate, and the associated median scores and p-value are presented in the following columns.

Chemical name	1	2	3	4	score median	p-value
Alverine citrate salt	3.63	-2.15			0.74	0.163
Benzthiazide	-0.95				-0.95	0.005
Dichlorphenamide	-1.01				-1.01	0.086
Haloperidol	0.37	-0.91	0.85		0.37	0.669
Hyoscyamine (L)	0.50				0.50	0.222
Oxymetazoline hydrochloride	-0.28				-0.28	0.637
Phentolamine hydrochloride	0.85	2.69			1.77	0.004
Scopolamin-N-oxide hydrobromide	-0.54	0.65			0.06	0.825
Tetrahydrozoline hydrochloride	0.89	-1.56			-0.33	0.073
Trichlormethiazide	-2.49	2.24			-0.12	0.010
Tropicamide	0.11	-2.34	1.30	0.92	0.52	0.462
Valdecoxib	0.18	1.66	0.56		0.56	0.001
Xylometazoline hydrochloride	0.93	-0.01			0.46	0.641
Aceclidine Hydrochloride	0.71	2.16	2.35		2.16	0.000
Benzoxiquine	-3.44				-3.44	0.003
Clemizole hydrochloride	-0.04	-1.53	1.43		-0.04	0.864
Digitoxigenin	2.09	0.92			1.50	0.000
Fenbufen	0.79	-1.87			-0.54	0.305
Iopromide	-2.87	-1.73			-2.30	0.001
Iproniazide phosphate	2.21	0.22			1.21	0.026
Mephenesin	3.23				3.23	0.000
Oxantel pamoate	-1.03	1.02			-0.01	0.957

Appendix Table S4. **Summary of the data generated for each Hit with the Opera Phenix - NMJ structure overlap.** Single associated z-scores for NMJ structure overlap, normalized with WT values are presented in columns named 1-4 for the *gan* MO quadruplicate, and the associated median scores and p-value are presented in the following columns.

Chemical name	1	2	3	4	score median	p-value
Alverine citrate salt	-2.30	-2.52			-2.41	0.017
Benzthiazide	-1.13				-1.13	0.267
Dichlorphenamide	0.35	-1.59	0.28		0.28	0.859
Haloperidol	-1.57	-1.77	-0.61		-1.57	0.047
Hyoscyamine (L)	-0.47				-0.47	0.533
Oxymetazoline hydrochloride	1.56				1.56	0.400
Phentolamine hydrochloride	0.36	0.47			0.41	0.600
Scopolamin-N-oxide hydrobromide	-0.98	-0.15			-0.57	0.267
Tetrahydrozoline hydrochloride	-1.08	-1.49			-1.28	0.067
Trichlormethiazide	2.71	1.50			2.11	0.067
Tropicamide	-0.84	-2.17			-1.50	0.067
Valdecoxib	-1.47	-2.33	0.43		-1.47	0.197
Xylometazoline hydrochloride	-0.69	-0.25			-0.47	0.417
Aceclidine Hydrochloride	0.36	-0.71	2.32	0.48	0.42	0.574
Benzoxiquine	1.20	-0.35	-2.70	1.57	0.43	0.878
Clemizole hydrochloride	-0.89	-2.39	0.21		-0.89	0.197
Digitoxigenin	3.99	0.91			2.45	0.150
Fenbufen	0.26	-1.57	-1.08		-1.08	0.197
Iopromide	0.39	-0.70	5.61		0.39	0.591
Iproniazide phosphate	-0.26				-0.26	0.800
Mephenesin	0.49	-0.90			-0.20	0.700
Oxantel pamoate	-1.09	1.75	10.99		1.75	0.432

Appendix Table S5. Summary of the z-scores coordinates (x (1) = #AchR clusters, y (2) = NMJ structure overlap, z (3) = Axonal length) for each selected Hit presented in 3-D scatter plot (**Fig 7D**). Their classification, identifier and most known Therapeutic class are provided.

Identifier	X = (1) #AchR clusters	Y = (2) NMJ structure overlap	Z = (3) Axonal length	Molecules	CAS number	Therapeutic class
1	1.54	0.42	2.16	Aceclidine Hydrochloride	6109-70-2	Ophthalmology
16	1.06	0.41	1.77	Phentolamine hydrochloride	73-05-2	Cardiovascular
7	1.83	2.45	1.50	Digitoxigenin	143-62-4	Cardiovascular
15	2.08	1.56	-0.28	Oxymetazoline hydrochloride	2315-02-8	Respiratory
19	0.78	2.11	-0.12	Trichlormethiazide	133-67-5	Cardiovascular
2	1.88	-2.41	0.74	Alverine citrate salt	5560-59-8	Neuromuscular
12	1.87	-0.26	1.21	Iproniazide phosphate	305-33-9	Cardiovascular
17	0.19	-0.57	0.06	Scopolamin-N-oxide hydrobromide	6106-81-6	Neuromuscular
21	1.09	-1.47	0.56	Valdecoxib	181695-72-7	Metabolism
22	0.14	-0.47	0.46	Xylometazoline hydrochloride	1218-35-5	Cardiovascular
9	-0.05	-1.57	0.37	Haloperidol	52-86-8	CNS
10	-0.08	-0.47	0.50	Hyoscyamine (L)	101-31-5	CNS
13	-0.03	-0.20	3.23	Mephenesin	59-47-2	CNS
20	-0.01	-1.50	0.52	Tropicamide	1508-75-4	Neuromuscular
3	-1.63	0.43	-3.44	Benzoxiquine	86-75-9	Infectiology
6	-0.03	0.28	-1.01	Dichlorphenamide	120-97-8	Ophthalmology

11	-1.01	0.39	-2.30	Iopromide	73334-07-3	Diagnostic
14	-0.82	1.75	-0.01	Oxantel pamoate	68813-55-8	Infectiology
4	0.23	-1.13	-0.95	Benzthiazide	91-33-8	Cardiovascular
5	0.42	-0.89	-0.04	Clemizole hydrochloride	1163-36-6	Allergology
8	0.05	-1.08	-0.54	Fenbufen	36330-85-5	CNS
18	-1.29	-1.28	-0.33	Tetrahydrozoline hydrochloride	522-48-5	Cardiovascular
